# Supplementary material for: Impact of essential workers in the context of social distancing for epidemic control
Source: PLoS One. 2021 Aug 4;16(8):e0255680. doi: 10.1371/journal.pone.0255680 (PMC8336873; doi:10.1371/journal.pone.0255680)
Supplement: S1 Appendix — (PDF) [file pone.0255680.s001.pdf]

Impact_of_Essential_Workers_Supplement

June 1, 2021

0.1 Impact of essential workers in the context of social distancing for epidemic control

The following notebook contains all code and scripts necessary to reproduce the results and figures from the main paper. In addition, there are explanations of the methods and supplementary figures to support the main text. Cells should be run sequentially.

0.1.1 S1. Model Code

This code implements and parameterizes our model. We use it later to generate our main and supplementary figures.

[1]:
```
# Libraries
# The code was initially inspired by the following sources:
# https://www.kaggle.com/pstarszyk/seir-model-with-extensions/
↪comments#Define-Extended-Model
# https://www.kaggle.com/anjum48/seir-hcd-model/data#SEIR-HCD-Model
# http://gabgoh.github.io/COVID/index.html
# https://neherlab.org/covid19/about

import pandas as pd
import numpy as np
import matplotlib as mpl
import matplotlib.pyplot as plt
import os
from scipy.integrate import solve_ivp
from scipy.optimize import minimize
from sklearn.metrics import mean_squared_log_error, mean_squared_error
from collections import namedtuple
from collections import defaultdict as ddict
from copy import deepcopy
import warnings
from datetime import datetime, timedelta
warnings.filterwarnings("ignore")
import seaborn as sns
import matplotlib.pylab as plt
from seaborn import heatmap
from pyDOE import *
```

```
[2]: # This is a function used to control the font size in generating the figures
def update_font_size(font_size = 30,rcParams = mpl.rcParams):
    rcParams.update({'font.size': font_size})
    plt.rc('font', size=font_size)          # controls default text sizes
    plt.rc('axes', titlesize=font_size)     # fontsize of the axes title
    plt.rc('axes', labelsize=font_size*1.1)     # fontsize of the x and y labels
    plt.rc('xtick', labelsize=font_size)    # fontsize of the tick labels
    plt.rc('ytick', labelsize=font_size)    # fontsize of the tick labels
    plt.rc('legend', fontsize=font_size)    # legend fontsize
    plt.rc('figure', titlesize=font_size)
```

We consider three separate SEIR models of essential workers: public-facing, non-public-facing, and healthcare workers. For simplicity, in the code these are referred to as 'Cashier', 'USPS', and 'Healthcare' models, respectively. Moreover, the 'No Structure' model refers to a model where there are no essential workers. This code block defines the names of these models.

```
[3]: # Make model labels a global so we can access anywhere
model_labels = {'Cashier'         :'Public Facing',
                'USPS'        :'Non-Public Facing',
                'Healthcare'  :'Healthcare',
                'No Structure':'No EWs',
                'Healthcare_Sigmoid'  :'Healthcare'}

# Plotting colors for each model
model_colors = {'No Structure':'k',
                'Cashier':[0/255,158/255,115/255,1],
                'Healthcare':[204/255,121/255,167/255,1],
                'USPS':[230/255,159/255,0/255,1],
                'Healthcare_Sigmoid':[204/255,121/255,167/255,1]}
```

The following code block contains the differential equations used in our compartmentalized SEIR model. We model the dynamics of the epidemic by considering different classes (shown below) and these equations describe the rate of movement between classes as a function of time. Following recent work, we expand on a standard SEIR epidemiological framework, by including additional comaprtments for infectious individuals and a hospitalized class that contains additional compartments for individuals requiring critical care.

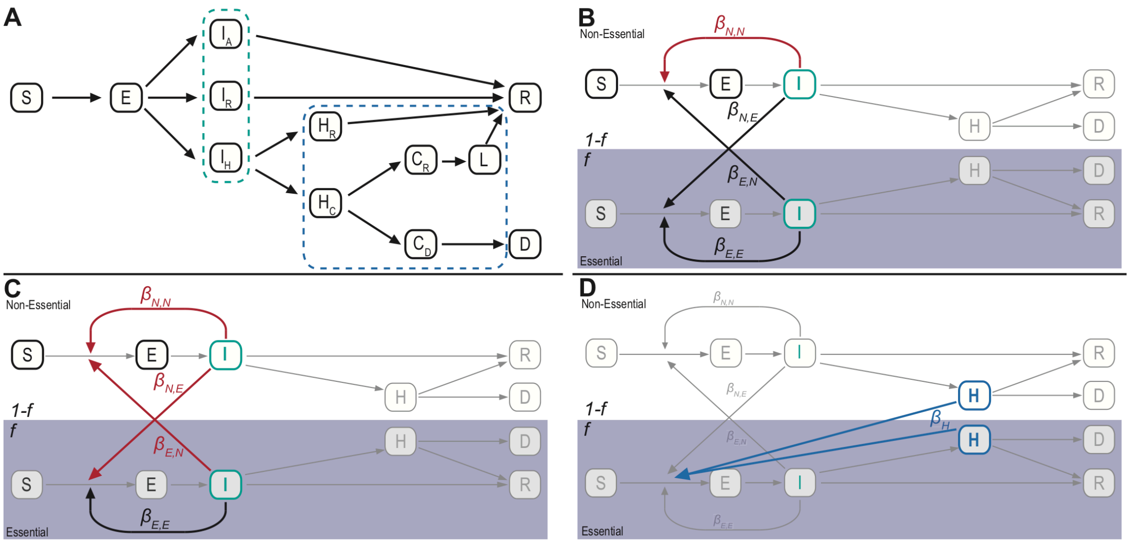

Here, 'S' is susceptible, 'E' is exposed, 'I' is infected, 'H' is hospitalized, 'D' is dead, and 'R' is recovered. Within the infected class, individuals can be asymptomatic 'I_A' and destined to recover; symptomatic 'I_R' but destined to recover; or symptomatic 'I_H' and destined to be hospitalized. Within the hospital, individuals either go to recovery 'H_R' or go to critical care 'H_C'. For those in critical care, individuals either die 'C_D' or go on to the recovered class 'C_R', with an additional time spent in the hospital 'L'. Above each compartment in blue are time parameters τ that describe how long individuals spend in each. Above arrows in red are parameters P that describe the probability an individual moves to a different class.

[4]:
```
# Susceptible equation
def dS_dt(y_i, p, N_i, I, Beta_t, H, Beta_hosp, groupID):
    if groupID == 1 and p.model == 'Healthcare_Sigmoid':
        return - y_i.S * (sum(Beta_t * I) * (1./N_i) + Beta_hosp)
    else:
        return -(1./N_i) * y_i.S * ( sum(Beta_t * I) + Beta_hosp * H)

# Exposed equation
def dE_dt(y_i, p, N_i, I, Beta_t, H, Beta_hosp, groupID):
    if groupID == 1 and p.model == 'Healthcare_Sigmoid':
        incoming = y_i.S * (sum(Beta_t * I) * (1./N_i) + Beta_hosp)
    else:
        incoming = (1./N_i) * y_i.S * ( sum(Beta_t * I) + Beta_hosp * H )
    return incoming - (y_i.E / p.t_inc)

# Infected, asymptomatic, and will recover equation
def dIA_dt(y_i, p,groupID):
    if groupID == 1:
        p_IA = p.p_IA * p.change_in_p_IA
    else:
        p_IA = p.p_IA
    return (y_i.E / p.t_inc) * p_IA - y_i.IA / p.t_AR

# Infected, symptomatic, and will recover equation
```

```python
def dIR_dt(y_i, p,groupID):
    if groupID == 1:
        p_IA = p.p_IA * p.change_in_p_IA
        p_IH = p.p_IH * p.change_in_p_IH
    else:
        p_IA = p.p_IA
        p_IH = p.p_IH
    return (y_i.E / p.t_inc)*(1-p_IA)*(1-p_IH) - y_i.IR / p.t_IR

# Infected and will require hospitalization equation
def dIH_dt(y_i, p,groupID):
    if groupID == 1:
        p_IA = p.p_IA*p.change_in_p_IA
        p_IH = p.p_IH*p.change_in_p_IH
    else:
        p_IA = p.p_IA
        p_IH = p.p_IH
    return (y_i.E / p.t_inc)*(1-p_IA)*p_IH  - y_i.IH / p.t_IH

# Recovered equation
def dR_dt(y_i, p,groupID):
    return  y_i.IR / p.t_IR + y_i.IA / p.t_AR + y_i.HR / p.t_HR + y_i.L / p.t_LR

# Hospitalized and will recover equation
def dHR_dt(y_i, p,groupID):
    return  y_i.IH / p.t_IH * (1 - p.p_HC) - y_i.HR / p.t_HR

# Hospitalized and will require critical care equation
def dHC_dt(y_i, p,groupID):
    return  y_i.IH / p.t_IH * p.p_HC - y_i.HC / p.t_HC

# Critical care and will recover equation
def dCR_dt(y_i, p,groupID):
    return  y_i.HC / p.t_HC * (1 - p.p_CD) - (y_i.CR / p.t_CL)

# Critical and will die equation
def dCD_dt(y_i, p, groupID):
    return y_i.HC / p.t_HC * p.p_CD - y_i.CD / p.t_CD

# Post-Critical care but still hospitalized equation
def dL_dt(y_i, p,groupID):
    return y_i.CR / p.t_CL - y_i.L / p.t_LR

# Dead equation
def dD_dt(y_i, p,groupID):
    return y_i.CD / p.t_CD
```

[5]:
```python
# Parameters: Using a namedtuple allows you to access parameters by name␣
↪instead of index (e.g. args.N)
parameters = namedtuple('parameters',["R_0","N","C",
                                                    ␣
↪"social_distancing_time","theta","model","proportion_essential","rho",
                                                    ␣
↪"max_days","num_groups","compartment_names",
                                                    ␣
↪"t_inc","t_AR","t_IR","t_IH","t_HR","t_HC","t_CD","t_CL","t_LR",
                                                     "p_IA","p_IH","p_HC","p_CD",
                                                    ␣
↪"change_in_p_IH","change_in_p_IA","adjust_beta_hosp"])

def create_parameters(args_default,set_parameters = {}):
    args_dict = args_default._asdict()
    for k,v in set_parameters.items():
        args_dict[k] = v
    new_args = parameters(**args_dict)
    return new_args


# Define the compartments and link names with formulas
compartment_names = ["S","E","IA","IR","IH","HR","HC","CR","CD","L","R","D"]
compartment_formulas =␣
↪[dS_dt,dE_dt,dIA_dt,dIR_dt,dIH_dt,dHR_dt,dHC_dt,dCR_dt,dCD_dt,dL_dt,dR_dt,dD_dt]
compartments = namedtuple("compartments",compartment_names)

# Initially assume patients in critical care are infectious
include_critical_care = True
```

[6]:
```python
# This function returns an array of differential equations that is used for the␣
↪ODE solver in the following section
def SEIR_HCD_model(t, y_vals, parameters):

    # Convert to matrix
    # Each row is a group
    # Each column corresponds to a given class within each group
    # Columns indexed according to position in name array: compartment_names =␣
↪["S", "E", "I", "R", "H", "C", "D"]
    y_matrix = np.reshape(y_vals,[parameters.num_groups,len(compartment_names)])

    output = np.zeros(np.shape(y_matrix))

    # Iterate over each group
    # Nonessential = 0
    # Essential = 1
```

```python
    for group_idx in range(parameters.num_groups):

        Beta_t_i = get_betas(t,parameters,group_idx)

        # Components for group i
        compartments_group_i = compartments(*y_matrix[group_idx,:])

        # Total population within group i
        N_i = sum(y_matrix[group_idx,:])

        # Get the infected population for each group for all 3 compartments
        I = sum(np.transpose(y_matrix[:,2:5]))

        # Get the hospitalized population for each group
        if include_critical_care:
            H = sum(sum(np.transpose(y_matrix[:,5:10])))
        else:
            H = sum(sum(np.transpose(y_matrix[:,5:7])))

        # If using a healthcare model and considering the healthcare group,
        # we need to consider infections stemming from hospitals
        if 'Healthcare' in parameters.model and group_idx == 1:

            # Determine the beta or fraction of workers infected
            beta_hosp = get_hosp_beta(t,parameters,H)

        else:

            beta_hosp = 0

        # Susceptibles and Exposed DiffEq use special parameters I and Beta_t_i)
        S_out_i = dS_dt(y_i = compartments_group_i,
                        p   = parameters,
                        N_i = N_i,
                        I   = I,
                        Beta_t = Beta_t_i,
                        H   = H,
                        Beta_hosp = beta_hosp,
                        groupID = group_idx)

        output[group_idx,0] = S_out_i

        E_out_i = dE_dt(compartments_group_i, parameters, N_i, I, Beta_t_i, H, beta_hosp, group_idx)
        output[group_idx,1] = E_out_i
```

```
        # The remainder of the differentiail equations
        for index in np.arange(2,len(compartment_names)):
            output[group_idx,index] =␣
↪compartment_formulas[index](compartments_group_i, parameters,group_idx)

    # Convert the matrix back into a linear array for the ODE solver
    output_array = np.reshape(output,[1,parameters.
↪num_groups*len(compartment_names)])[0]

    return output_array
```

In the model, we use β to represent the number of contacts an individual has per day. Social distancing and other shelter in place (SIP) measures reduce β by a parameter by θ. We define θ as the remaining proportion of individual to individual transmission after social distancing, where $\theta = 1$ corresponds to no social distancing and $\theta = 0$ is complete isolation. In the various models of essential workers we consider, θ is applied to different β's to reduce interactions according to how they are defined in the main text. These functions are used to get the β terms under the specified model.

[7]:
```
# Return the matrix of thetas that determine how to adjust betas given lockdown
def get_effect_of_social_distancing(p,t=0):
    if p.model == 'No Structure' or (p.rho > 0 and (p.model == 'USPS' or p.
↪model == 'Healthcare' or p.model == 'Healthcare_Sigmoid')):
        social_distancing_effect = np.array([p.theta]*4)

    elif p.model == 'Cashier':
        social_distancing_effect = np.array([p.theta]+[1]*3)

    elif p.model == 'USPS' or p.model == 'Healthcare' or p.model ==␣
↪'Healthcare_Sigmoid':

        social_distancing_effect = np.array([p.theta]*3 + [1])

    else:
        raise ValueError('Model should be either Cashier,USPS,or No Structure. '
                         + p.model + ' is not an implemented model')

    social_distancing_effect = np.reshape(social_distancing_effect,[p.
↪num_groups,p.num_groups])

    return social_distancing_effect

# Return the beta for within hospital interactions
def get_hosp_beta(t,p,H):
    if p.model == 'Healthcare_Sigmoid':
        return get_sigmoid_beta(t,p,H)
    else:
```

```
        # Default: assume the within beta hospital interaction is the same as␣
↪the beta between I compartments and S
        return get_infectious_beta(p)*p.adjust_beta_hosp

def get_sigmoid_beta(t,p,H):

    exponent_parameter = 1
    halfway_point      = 1/4
    max_infection_rate = get_infectious_beta(p)*p.adjust_beta_hosp*1.5*H_max/p.N

    halfway_exponent    = halfway_point**exponent_parameter
    h_exponent = (H/H_max)**exponent_parameter
    return ( max_infection_rate * ( h_exponent ) / (␣
↪h_exponent+halfway_exponent ) )

def get_infectious_beta(p,model = ''):

    # llow user to set model, but otherwise use parameter model
    if not model:
        model = p.model

    # Fraction of essential workers
    f     = p.proportion_essential

    # Average time spent in I compartments
    avg_time_infectious = (p.p_IA * p.t_AR + (1 - p.p_IA) * ((1-p.p_IH) * p.
↪t_IR + p.p_IH * p.t_IH))

    # Average time spent in the hospital
    if model == 'Healthcare' and include_critical_care == True:
        avg_time_critical = (1 - p.p_CD) * (p.t_CL + p.t_LR) + p.p_CD * (p.t_CD)
        avg_time_hospital = (1-p.p_HC) * p.t_HR + p.p_HC * (p.t_HC␣
↪+avg_time_critical)
        avg_time_healthcare = (1 - p.p_IA) * p.p_IH * avg_time_hospital

    elif model == 'Healthcare' and include_critical_care == False:
        avg_time_hospital = (1-p.p_HC) * p.t_HR
        avg_time_healthcare = (1 - p.p_IA) * p.p_IH * avg_time_hospital

    else:
        # ignore if not a healthcare model
        avg_time_healthcare = 0

    # Get the mixing matrix or set to proportional mixing if not provided
    C = p.C
    if C == []:
        C = np.array([[1-f,1-f],[f,f]])
```

```
    # Vector to describe the distribution of people
    people_array = np.array([1-f,f])

    # The weighted sum across mixings
    beta_coefficient = avg_time_infectious*(sum(np.matmul(C,people_array)) + p.
 ↪rho * f)

    hosp_coefficient   = avg_time_healthcare
    beta = p.R_0/(beta_coefficient + hosp_coefficient * p.adjust_beta_hosp *␣
 ↪keep_R0_constant)

    return beta

# This allows for greater within-group mixing than under proportional mixing
def get_extra_rho_effect(t,p,groupID):
    if groupID != 1:
        return 0

    if t > p.social_distancing_time and p.model == 'No Structure':
            return p.rho * (p.theta)
    return p.rho

def get_betas(t,p,groupID,model = ''):

    if not model:
        model = p.model
    social_distancing_effect = get_effect_of_social_distancing(p,t)
    if t>p.social_distancing_time:
        social_distancing_effect = social_distancing_effect[:,groupID]
    else:
        social_distancing_effect = social_distancing_effect[:,groupID] * 0 + 1

    # beta is R_0 * mean infectious time
    # This is the same beta across all infectious compartments and groups
    nointervention_beta = get_infectious_beta(p,model)

    # This gives you the betas including the mixing between groups (specific to␣
 ↪group i)

    betas = list(nointervention_beta*social_distancing_effect*p.C[groupID,:])

    extra_rho = get_extra_rho_effect(t,p,groupID)
    betas[1] += extra_rho * nointervention_beta

    return betas
```

The parameters we use for the model come from estimates reported in Table 1.

Parameter	Variable Name	Value
R_0	R_0	3
τ_E	t_inc	3
τ_{IA}	t_AR	5
τ_{IR}	t_IR	5
τ_{IH}	t_IH	5
τ_{HR}	t_HR	8
τ_{HC}	t_HC	6
τ_{CR}	t_CL	7
τ_L	t_LR	3
τ_{CD}	t_CD	10
P_{EIA}	p_IA	1/3
P_{EIH}	p_IH	0.044
P_{IHC}	p_HC	0.3
P_{CD}	p_CD	0.5

Note that P_{EIH} is listed as 0.044 in the table above, but 0.066 in the parameters below. This is because in the code, this value is conditional on not being asymptomatic.

[8]:
```
#These are the parameters used in the models. These are listed in Table 1 of␣
↪the main text.
keep_R0_constant = False
args_default = parameters(R_0 = 3,                              # R_0: used to␣
↪calculate betas
                          N   = 8e6,                                  # total␣
↪population size
                          C   = [],                                  # Mixing␣
↪matrix

                          social_distancing_time = 53,    # When does social␣
↪distancing start
                          theta = 1,                          # How effective is␣
↪social distancing
                          model = 'Cashier',                         # Who␣
↪social distances?
                          proportion_essential = 0.1, # What fraction of the␣
↪population is essential workers?
                          rho = 1,
```

```
                          max_days = 1000,                   # bookkeeping of␣
↪how many days simulated
                          num_groups = 2,               # bookkeeping of how␣
↪many groups
                          compartment_names = compartment_names, # bookkeeping␣
↪of how many compartments

                          t_inc =  3,   # !time from E to I: exposed to infected
                          t_AR =   5,   # !time from IA to R: infected and␣
↪asymptomatic to recovered
                          t_IR =  5,   # !time from IR to R: infected,␣
↪symptomatic and will recover to recovered
                          t_IH =  5,   # !time from IH to H: infected and will␣
↪need hospitalization to hospitalization
                          t_HR =  8,   # !time from HR to R: hospitalized and␣
↪will recover to recovered
                          t_HC =   6,   # !time from HC to C: hospitalization␣
↪to critical care
                          t_CD =   10,# time from CD to D: critical care and␣
↪will die to death
                          t_CL =   7,   # time from CR to L: critical care and␣
↪will recover to post-critical care hospitalization
                          t_LR =  3,   # time from L to R: post-critical care␣
↪hospitalization to recovered

                          p_IA = 1/3, # Proportions of infections that are␣
↪asymptomatic
                          p_IH = 0.066, # Proportion of symptomatic infections␣
↪that require hospitalizations (this corresponds to 4.4% of all infections␣
↪that require hospitalization)
                          p_HC = 0.3, # Proportion of hospitalizations that␣
↪require critical care
                          p_CD = 0.5, # Proportion of critical cases that are␣
↪fatal
                          change_in_p_IH = 1, # How much is the probability of␣
↪hopsitalization increased in OLD
                          change_in_p_IA = 1, # How much is the probability of␣
↪asymptomatic increased in OLD
                          adjust_beta_hosp = 1) # change the beta for within␣
↪hospital interactions relative to beta between I and S

adjust_beta_hosp = 11.35713571
```

The following code block contains some helper functions to return the counts of individuals within each compartment at the end of the modelling.

[9]:
```
# Returns number of cases, hospitalizations, and deaths given model solution,␣
↪number of groups, and compartment names
def get_compartment_counts(solution, num_groups,
                           compartment_names =␣
↪["S","E","IA","IR","IH","HR","HC","CR","CD","L","R","D"]):

    # convert solution into a matrix
    max_days = len(solution.y[0])
    solution_matrix = np.reshape(solution.
↪y,[num_groups,len(compartment_names),max_days])

    # sum across groups
    total_solution = np.zeros([len(compartment_names),max_days])
    for i in range(num_groups):
        total_solution += solution_matrix[i]

    # sum across relevant compartments
    cases = sum(total_solution[2:])
    deaths = total_solution[-1]
    hospitilized = sum(total_solution[5:10])

    return cases, hospitilized, deaths
    return 2.5
```

Below is the main function used to return the results from a given model of essential workers and set of parameters.

[10]:
```
#This function will run the model above, and return the solution and parameters␣
↪used in a tuple
def run_model(theta,
              t_lockdown=args_default.social_distancing_time,
              R_0 = args_default.R_0,
              model = 'No Structure',
              n_infected=20,
              n_exposed=0,
              prop_essential=0.05,
              max_days = 1000,
              rho = 0,
              change_in_p_IH = 1,
              change_in_p_IA = 1,
              num_groups = 2,
              doubling_time = None,
              adjust_beta_hosp = adjust_beta_hosp,
              set_parameters = {}):
```

```
    # Social interaction effects: C
    # C[i][j] = proportion of group I's contacts that are with group
    # rho allows deviations from proportionate mixing

    C = np.array([    (1 - prop_essential) + rho * prop_essential,
                      (1 - rho) * (1 - prop_essential),
                      (1 - rho) * (prop_essential),
                      1 - (1 - rho) * (1 - prop_essential) - rho])

    C = np.reshape(C,[2,2])

    set_parameters.update({'R_0': R_0,
                          'C':C,
                          'social_distancing_time':t_lockdown,
                          'theta':theta,
                          'model':model,
                          'proportion_essential':prop_essential,
                          'rho':rho,
                          'max_days':max_days,
                          'adjust_beta_hosp':adjust_beta_hosp
                          })
    args = create_parameters(args_default =␣
↪args_default,set_parameters=set_parameters)

    single_population     = np.zeros(len(compartment_names))
    single_population[1]  = n_exposed
    single_population[2]  = n_infected*args.p_IA
    single_population[3]  = n_infected*(1-args.p_IA)*(1-args.p_IH)
    single_population[4]  = n_infected*(1-args.p_IA)*(args.p_IH)
    single_population[0]  = args.N - sum(single_population)
    initial_state         = [(1 - prop_essential) * single_population,
                              prop_essential * single_population]
    # Convert initial state into 1-dimensional array
    initial_state = [params for group in initial_state for params in group]

    # Evaluate the model
    # Here, a stiff solver (Radau) not RK45 (default) is used
    sol = solve_ivp(lambda t,y: SEIR_HCD_model(t,y,args), [0, max_days],␣
↪initial_state, t_eval=np.arange(max_days),method = 'Radau')
    return (sol,args)
```

The following functions are used to return various results from the output of a model run, as well as to set up a grid for plotting time courses and showing figure legends:

[11]:
```
# Get the cumulative infection rate at some time
def get_infection_rate(solution, p, time = 365):
```

```
    # Turn the solution array into a matrix
    solution_matrix = np.reshape(solution.y,[p.num_groups,len(p.
 ↪compartment_names),p.max_days])

    # If no time specified, get cumulative infection at end of time
    if not time or time == -1:
        time = p.max_days-1

    # Get the number of infections, return it as a fraction of the populations
    infections = sum(sum(solution_matrix[:,1:,time]))
    return infections/p.N

# Return the metrics to show the added disease risk within the essential workers
def added_disease_risk(solution, p,method = 'g(t)',groupID = 1):

    solution_matrix = np.reshape(solution.y,[p.num_groups,len(p.
 ↪compartment_names),p.max_days])
    f = p.proportion_essential
    if method == 'Fraction Infected':
        if groupID == 1:
            N = (p.N*(p.proportion_essential))
        else:
            N = (p.N*(1-p.proportion_essential))
        return sum(solution_matrix[groupID,1:,-1])/N
    elif method == 'Nonessential Comparison':
        L = sum(solution_matrix[1,1:,-1])/sum(solution_matrix[0,1:,-1])*((1-f)/
 ↪f)
        return L
    elif method == 'g(t) additive':
        return sum(solution_matrix[1,1:,-1])/(sum(solution_matrix[1,1:
 ↪,-1])+sum(solution_matrix[0,1:,-1]))
    elif method == 'g(t) multiplicative':
        return sum(solution_matrix[1,1:,-1])/(sum(solution_matrix[1,1:
 ↪,-1])+sum(solution_matrix[0,1:,-1]))/f
    elif method == 'EisenA':
        return sum(solution_matrix[1,1:,-1])/(p.N*f)-sum(solution_matrix[0,1:
 ↪,-1])/(p.N*(1-f))

    elif method == 'No Structure Comparison':
        NS_sol,NS_p = run_model(theta=p.theta,
                                R_0=p.R_0,
                                t_lockdown=p.social_distancing_time,
                                model='No Structure',
                                n_infected=20,
                                n_exposed=0,
```

```
                                    prop_essential=p.proportion_essential,
                                    max_days = p.max_days,
                                    rho = 0,
                                    change_in_p_IH = p.change_in_p_IH,
                                    change_in_p_IA = p.change_in_p_IA,
                                    doubling_time = 3,
                                    adjust_beta_hosp = 1)

        NS_solution_matrix = np.reshape(NS_sol.y,[p.num_groups,len(p.
 ↪compartment_names),p.max_days])
     # Turn the solution array into a matrix

        return sum(solution_matrix[1,1:,-1])/sum(NS_solution_matrix[1,1:,-1])
    else:
        raise ValueError("Method not understood")

# Given a solution to the model and the corresponding parameters, get several␣
 ↪different metrics of model outcomes
def get_metrics(result_dict):

    # Create a nested dict, where the structure is {model:{theta:{f:([],[])}}}
    reversed_solved_dict = ddict(dict)
    for model,model_dict in result_dict.items():
        reversed_solved_dict[model] = ddict(dict)
        for f,f_dict in model_dict.items():
            for theta,(sol,args) in f_dict.items():
                reversed_solved_dict[model][theta][f] =␣
 ↪(get_infection_rate(solution=sol,p=args, time = 365),
                                                                      ␣
 ↪added_disease_risk(solution=sol,p = args,method = 'Fraction␣
 ↪Infected',groupID = 0),
                                                                      ␣
 ↪added_disease_risk(solution=sol,p = args,method = 'Fraction␣
 ↪Infected',groupID = 1))
    return reversed_solved_dict

# Assign row and column labels for the plots of the effective R over time
def assign_row_label_grid(ax,f, pad = 5, xy = (0,0.5),font_size = 30):

    ax.annotate('f = {}'.format(f)+'\n', xy=xy, xytext=(-ax.yaxis.labelpad -␣
 ↪pad, 0),
                xycoords=ax.yaxis.label, textcoords='offset points',
                size=font_size, ha='right', va='center',rotation=90)

# Label is the value of R_effective
def assign_column_label_grid(ax,value, pad = 5, xy = (0.5,1),font_size = 30):
```

```python
    text = r'$R_0 \theta = ${}'.format(round(value,2))
    ax.annotate(text, xy=xy, xytext=(0, pad),
                xycoords='axes fraction', textcoords='offset points',
                size=font_size, ha='center', va='baseline')

def get_R_effective(solution_matrix,p):

    # Set up an empty array for both effective R and the betas over time
    R_eff     = np.zeros(p.max_days+1)
    betas_eff = np.zeros([2,2,p.max_days+1])

    # Get the time values
    t     = np.arange(0,p.max_days)

    # Fraction of population an essential worker
    f     = p.proportion_essential

    # Get average time an infectious person is infectious
    avg_time_infectious = (p.p_IA * p.t_AR + (1 - p.p_IA) * ((1-p.p_IH) * p.
 ↪t_IR + p.p_IH * p.t_IH))

    # Find the effects of social distancing. Second variable is from a␣
 ↪different implementation
    social_distancing_effect = get_effect_of_social_distancing(p,None)

    # beta is R_0 * mean infectious time
    # This is the same beta across all infectious compartments and groups
    nointervention_beta = get_infectious_beta(p)
    # Get fraction of infected population that is essential over time

    I_t_0   = sum(solution_matrix[0,2:5,:])
    I_t_1   = sum(solution_matrix[1,2:5,:])
    S_t_0   = solution_matrix[0,0,:]/(p.N*(1-f))
    S_t_1   = solution_matrix[1,0,:]/(p.N*f)
    f_t_all = (I_t_1*f)/(I_t_0*(1-f)+I_t_1*f)
    for j in t:

        # Get fraction of infected that are essential at this time, j
        f_t  = f_t_all[j]

        # Calculate betas. This is elementwise multiplication not matrix␣
 ↪multiplication
        betas = nointervention_beta*(p.C*(1-social_distancing_effect*(j>p.
 ↪social_distancing_time)))
        betas_eff[:,:,j] = betas
```

```
        # Include extra effect from within group mixing
        betas_eff[1,1,j] += get_extra_rho_effect(j,p,1) * nointervention_beta

        # Calculate R effective by weighting betas by f(t) and the average␣
↪infectious time
        R_eff[j] = sum(np.matmul(betas_eff[:,:,j]*np.
↪array([S_t_0[j],S_t_1[j]]),np.array([1-f_t,f_t])))
        R_eff[j] = R_eff[j]*avg_time_infectious

        # If a healthcare model, include the within hospital infections
        if 'Healthcare' in p.model:
            H    = sum(np.transpose(solution_matrix[:,5:10,j]))
            f_th  = (H[1]*f)/(H[0]*(1-f)+H[1]*f)

            hosp_beta = get_hosp_beta(j,p,sum(H))
            avg_time_healthcare = (1 - p.p_IA) * p.p_IH * ((1 - p.p_HC) * p.
↪t_HR + p.p_HC * (p.t_HC + (1 - p.p_CD) * (p.t_CL + p.t_LR) + p.p_CD * p.
↪t_CD))
            R_eff[j] += avg_time_healthcare*hosp_beta

    return R_eff,betas_eff,f_t_all

def create_legend(fig,models: list,ls_styles: dict,linewidth = 2,legend_entries␣
↪= {},legend_keywords = {},font_size = 10):

    legend_entries['EWs'] = mpl.lines.Line2D([],[],color = [0.5,0.5,0.5],ls =␣
↪'--', linewidth = linewidth)
    legend_entries['nEWs'] = mpl.lines.Line2D([],[],color = [0.5,0.5,0.5],ls =␣
↪'-', linewidth = linewidth)

    for model in models:
        ls,color,zorder = ls_styles[model]
        legend_entries[model_labels[model]] = mpl.lines.Line2D([],[],color =␣
↪color,ls = ls, linewidth = linewidth)


    legend = fig.legend(legend_entries.values(),legend_entries.
↪keys(),**legend_keywords,fontsize = font_size)
    return legend

def plot_grid(theta_values,
              metric = 'All_Infection',
              models = ['Cashier','USPS','Healthcare','No Structure'],
              t_lockdown = args_default.social_distancing_time,
              f_values = [0.05],
              max_days = 300,
```

```
              R_0=args_default.R_0,
              I0=20,
              adjust_beta_hosp = adjust_beta_hosp,
              figsize = (7.5,3),
              model_dependent_rho = {'No Structure': 0,
                                     'Cashier': 0,
                                     'Healthcare': 0.5,
                                     'USPS': 0.5,
                                     'Healthcare_Sigmoid':  0},
              linewidth = 1,
              font_size = 9,
              figure_title = '',
              set_parameters = {},
              legend_loc = (0.807, 0.575)):

    update_font_size(font_size)
    fig, axes = plt.subplots(len(f_values),␣
↪len(theta_values),figsize=figsize,dpi = 300)

    # Plotting LS and color for each model
    ls_styles = {'No Structure':('-',model_colors['No Structure'],1),
                 'Cashier':('-',model_colors['Cashier'],100),
                 'Healthcare':('-',model_colors['Healthcare'],100),
                 'USPS':('-',model_colors['USPS'],100),
                 'Healthcare_Sigmoid':('-',model_colors['Healthcare'],100)}

    for i,f in enumerate(f_values):
        for j,theta in enumerate(theta_values):
            for model_index,model in enumerate(models):

                rho = model_dependent_rho[model]

                # run model and reshape output
                solution,p = run_model(theta = theta,
                                       R_0 = R_0,
                                       t_lockdown = t_lockdown,
                                       model = model,
                                       n_infected = I0,
                                       n_exposed  = I0,
                                       prop_essential = f,
                                       max_days = max_days,
                                       rho = rho,
                                       adjust_beta_hosp = adjust_beta_hosp*f,
                                       set_parameters = set_parameters)

                solution_matrix = np.reshape(solution.y,[p.num_groups,len(p.
↪compartment_names),p.max_days])
```

```
            # Get average cumulative infection rate across population
            avg_infection_rate = sum(sum(solution_matrix[:,1:,:]))/8e6

            # Get proportion of population in each compartment relative to␣
↪group size
            # Note, here N is hardcoded as 8e6 (approx. NYC)
            solution_matrix[0,:,:] = solution_matrix[0,:,:]/(8e6*(1-f))
            solution_matrix[1,:,:] = solution_matrix[1,:,:]/(8e6*f)

            # Get the effective R, betas, and g(t)
            R_effective, Betas, G = get_R_effective(solution_matrix,p)
            R_effective = R_effective[:-1]
            R_effective[:t_lockdown+1] = np.nan

            # Get color for each model but don't use LS
            ls, color, zorder = ls_styles[model]
            ls = '-'

            if len(f_values) > 1:
                if len(theta_values) > 1:
                    ax = axes[i][j]
                else:
                    ax = axes[i]
            else:
                if len(theta_values) > 1:
                    ax = axes[j]
                else:
                    ax = axes

            plt.sca(ax)

            if metric == 'Average_Infection':
                # Plot cumulative infection rate in population
                plt.plot(np.arange(0,max_days),avg_infection_rate,ls =␣
↪'-',color=color,label=model,linewidth = linewidth,zorder=zorder)
                plt.ylabel("Cumulative Infections")

            elif metric == 'Essential_Infection':
                plt.plot(np.arange(0,max_days),sum(solution_matrix[1,1:,:
↪]),ls = '--',color=color,linewidth = linewidth,alpha = 1,zorder=zorder)
                plt.ylabel("Cumulative Infections\nin Essential Workers")

            elif metric == 'Nonessential_Infection':
                plt.plot(np.arange(0,max_days),sum(solution_matrix[0,1:,:
↪]),ls = ':',color=color,linewidth = linewidth,alpha = 1,zorder=zorder)
```

```
                    plt.ylabel("Cumulative Infections\nin Non-Essential␣
↪Workers")

                elif metric == 'All_Infection':
                    plt.plot(np.arange(0,max_days),sum(solution_matrix[1,1:,:
↪]),ls = '--',color=color,linewidth = linewidth,alpha = 1,zorder=zorder)
                    plt.plot(np.arange(0,max_days),sum(solution_matrix[0,1:,:
↪]),ls = '-',color=color,linewidth = linewidth,alpha = 1,zorder=zorder)
                    plt.ylabel("Cumulative Infections")

                elif metric == 'R(t)':
                    plt.plot(np.arange(0,max_days),R_effective,color =␣
↪color,linewidth = linewidth,ls=ls,label=model,rho = 1,zorder=zorder)
                    plt.ylabel("R(t)")

                else: raise ValueError('Metric "{}" not understood'.
↪format(metric))


                plt.xlabel("Days")
                plt.xlim([0,max_days-100])

                if metric != 'R(t)':
                    plt.fill_betweenx(y=[1e-7,1],x1=0,x2=p.
↪social_distancing_time,color=[0.8,0.8,0.8,0.5])

                # Assign a column label
                if i == 0 and len(theta_values) > 1:
                    assign_column_label_grid(ax,R_0*(theta),font_size =␣
↪font_size)
                # Assign a row column
                if j == 0 and len(f_values) > 1:
                    assign_row_label_grid(ax,f,font_size = font_size)

    legend = create_legend(fig = fig,
                           ls_styles = ls_styles,
                           models = models,
                           linewidth = linewidth,
                           legend_keywords = {'loc':'upper left',
                                              'bbox_to_anchor':legend_loc,
                                              'framealpha': 1,
                                              'edgecolor':'k'},
                          font_size = 8)
    for ax in axes.flat:
        ax.tick_params(width = 1, length = 4)
        ax.tick_params(width = 1, length = 2.5,which='minor')
```

```
    plt.subplots_adjust(wspace=0.372,hspace=0,right=0.84,left=0)
    fig.suptitle(figure_title)
    plt.show()


# keeps track of supplementarty figure numbers
fn = 0
def figure_number():
    global fn
    fn += 1
    return fn
```

[ ]:

0.1.2 S2 - Cumulative infection rates among groups

Using the model, we can now look at the cumulative infection rates among essential and non-essential workers under various parameter values. The following code reproduces Figure 2 from the main text, and uses $f = 0.05$ and a lockdown time of 47 days after the start of the infection.

[12]:
```
# Make plot for Figure 2
desired_Reff = np.array([0.5,0.9,1.5])
R_0 = args_default.R_0

plt.rc('legend', fontsize=8)
plot_grid(theta_values = desired_Reff/R_0)

print(desired_Reff/R_0)
```

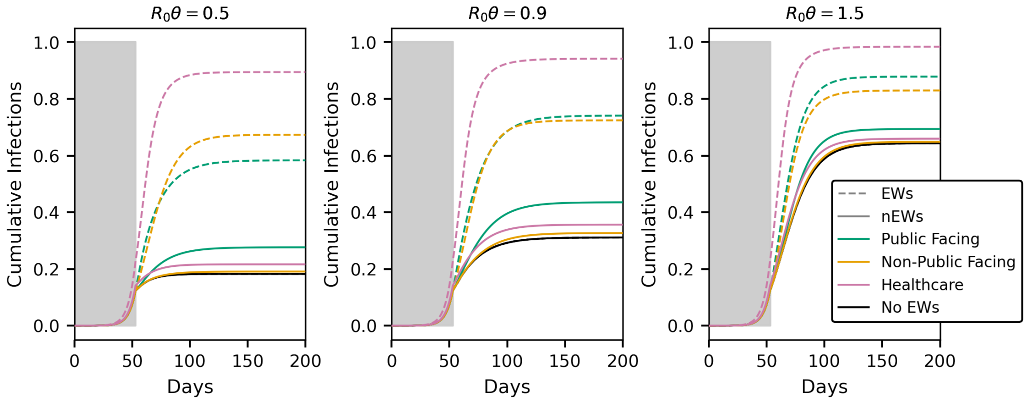


```
[0.16666667 0.3        0.5       ]
```

Alternative versions of figure 2 with different parameter values We reproduce figure 2 while varying individual parameters or relaxing model assumptions.

[13]:
```
# Make a similar plot to Figure 2, but change f
plt.rc('legend', fontsize=30)
plot_grid(theta_values = desired_Reff/R_0,
          f_values     = [0.10])
```

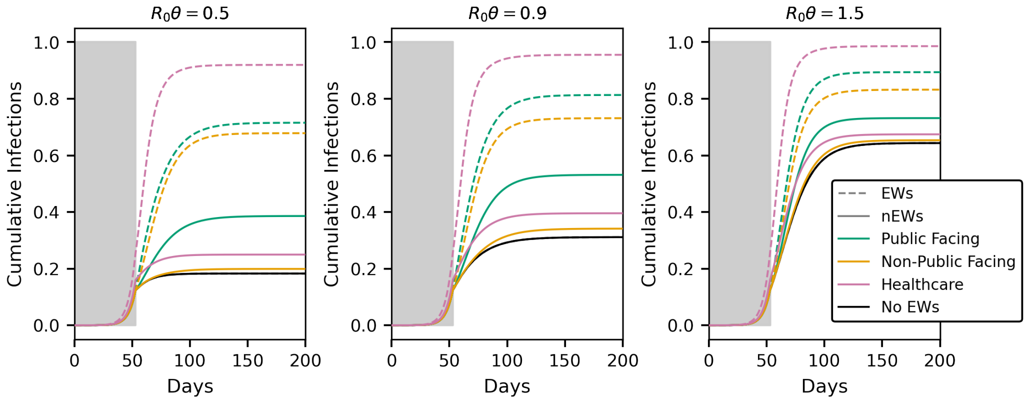

Figure S1. Cumulative infection rates among groups assuming $f = 0.1$. See Figure 2 for details.

[14]:
```
change_t_lockdown = -10
plot_grid(theta_values = desired_Reff/R_0,
              t_lockdown    = args_default.
 ↪social_distancing_time+change_t_lockdown)
```

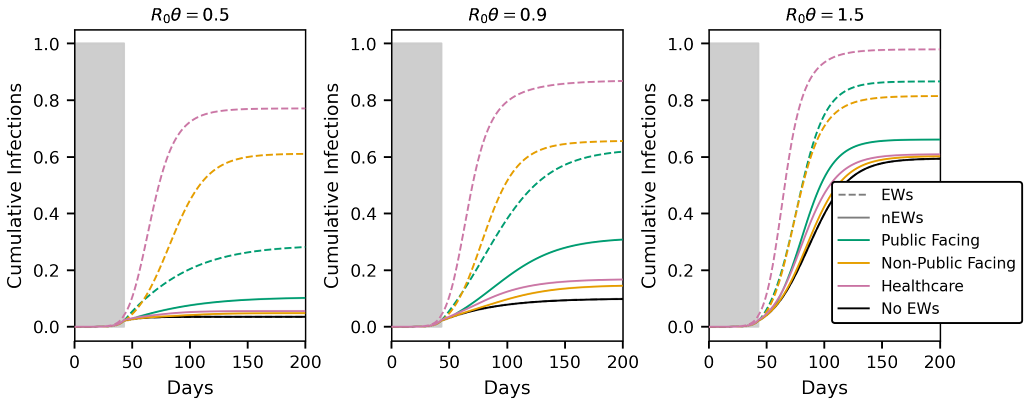

Figure S2. Cumulative infection rates among groups assuming lockdown 10 days earlier. See Figure 2 for details.

[15]:
```
n_infected = 10
plot_grid(theta_values = desired_Reff/R_0,
          I0           = n_infected)
```

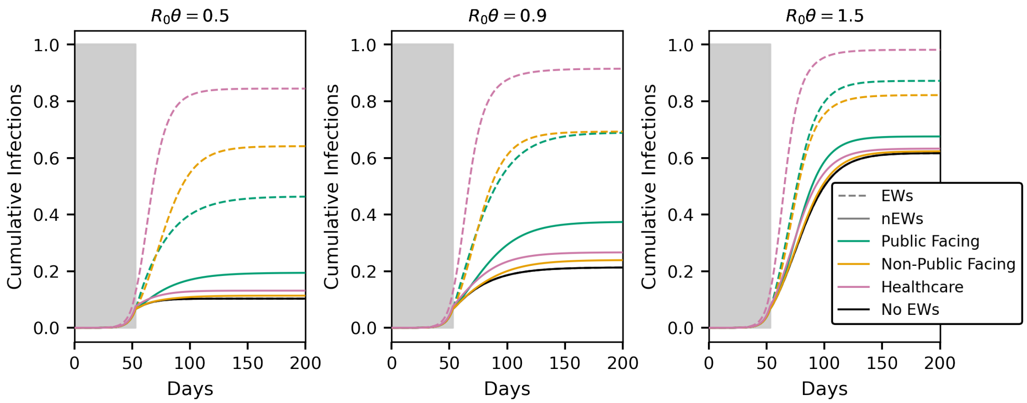

Figure S3. Cumulative infection rates among groups when halving the initial number of infected individuals ($I_0 = 10$). See Figure 2 for details.

[16]:
```
n_infected = 40
plot_grid(theta_values = desired_Reff/R_0,
          I0           = n_infected)
```

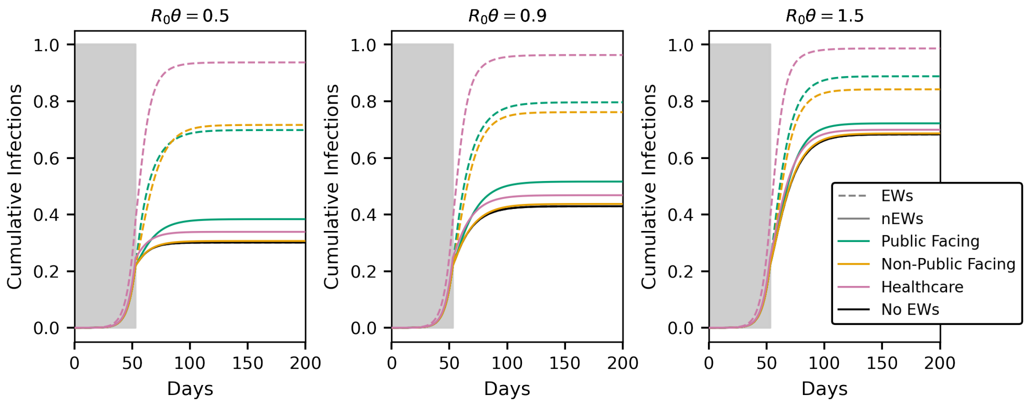

Figure S4. Cumulative infection rates among groups when doubling the initial number of infected individuals ($I_0 = 40$). See Figure 2 for details.

[17]:
```
R_0 = 2.5
plot_grid(theta_values = desired_Reff/R_0,
          R_0 = R_0,
          max_days = 500)
```

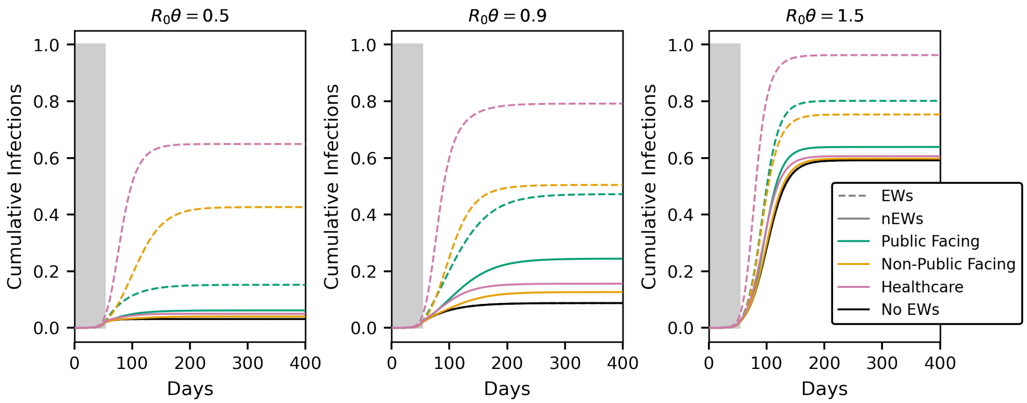

Figure S5. Cumulative infection rates among groups when reducing R_0 ($R_0 = 2.5$). See Figure 2 for details.

[18]:
```
R_0 = 3.5
plot_grid(theta_values = desired_Reff/R_0,
          R_0 = R_0,
          max_days = 500)
R_0 = args_default.R_0
```

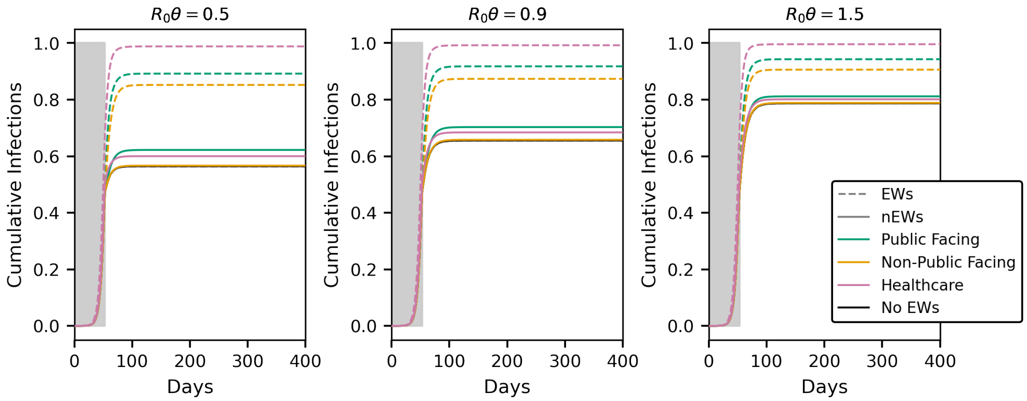

Figure S6. Cumulative infection rates among groups when reducing R_0 ($R_0 = 3$). See Figure 2 for details.

[19]:
```
change_in_incubation_time = -1
set_parameters = {'t_inc':3+change_in_incubation_time}
plot_grid(theta_values = desired_Reff/R_0,
          set_parameters = set_parameters)
```

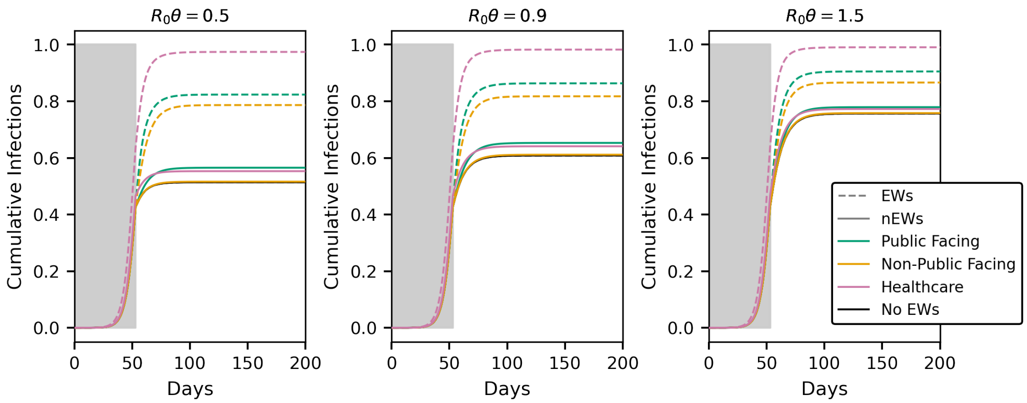

Figure S7. Cumulative infection rates among groups when decreasing the incubation time to two days.

[20]:
```
change_in_incubation_time = 1
set_parameters = {'t_inc':3+change_in_incubation_time}
plot_grid(theta_values = desired_Reff/R_0,
          set_parameters = set_parameters,
          max_days = 500)
```

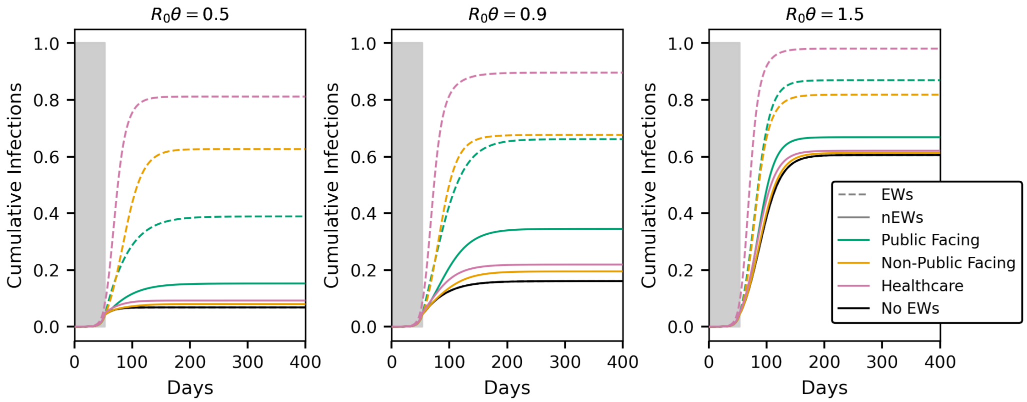

Figure S8. Cumulative infection rates among groups when increasing the incubation time to four days.

[21]:
```
increase_time_infectious = 5
set_parameters = {'t_AR':5 + increase_time_infectious,
                  't_IR':5 + increase_time_infectious,
                  't_IH':5 + increase_time_infectious}
plot_grid(theta_values = desired_Reff/R_0,
          max_days = 800,
```

```
          set_parameters = set_parameters)
```

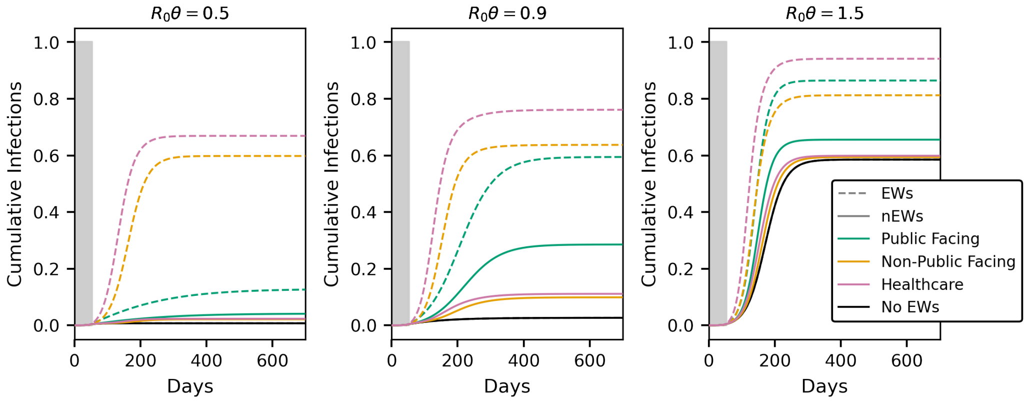

Figure S9. Cumulative infection rates among groups when doubling the average time of infectiousness. This delays the pandemic as a longer time of infectiousness but constant R_0 forces a reduction in the β terms. See Figure 2 for details.

[22]:
```
rho = 0
plot_grid(theta_values = desired_Reff/R_0,
         model_dependent_rho = {'No Structure': rho,
                                'Cashier': rho,
                                'Healthcare': rho,
                                'USPS': rho,
                                'Healthcare_Sigmoid':  rho},
          max_days = 500)
```

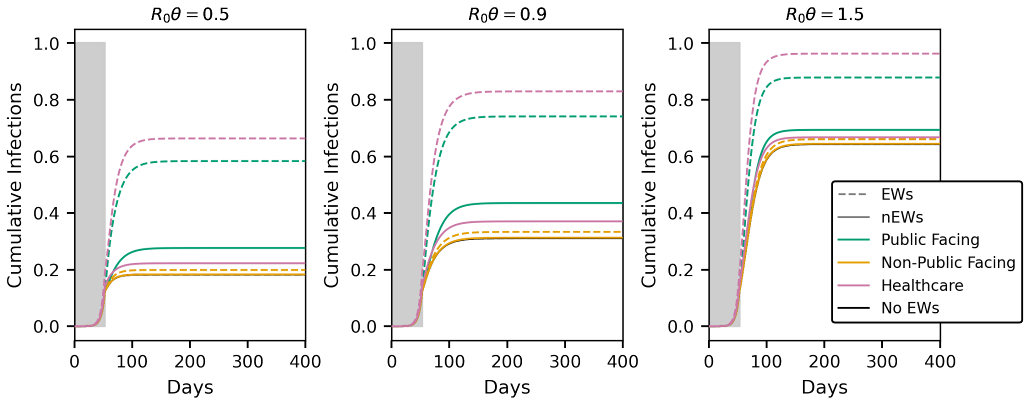

Figure S10. Cumulative infection rates among groups when all contacts are distributed proportionally (i.e., $\rho = 0$). In the non-public facing model, only 2.5% of contacts are not affected by SIP so EWs have little impact on the pandemic. The healthcare EW model remains qualitatively similar;

this is the public-facing model shown in the main. See Figure 2 for details.

[23]:
```
rho = 0.5
plot_grid(theta_values = desired_Reff/R_0,
          model_dependent_rho = {'No Structure': rho,
                                 'Cashier': rho,
                                 'Healthcare': rho,
                                 'USPS': rho,
                                 'Healthcare_Sigmoid':  rho})
```

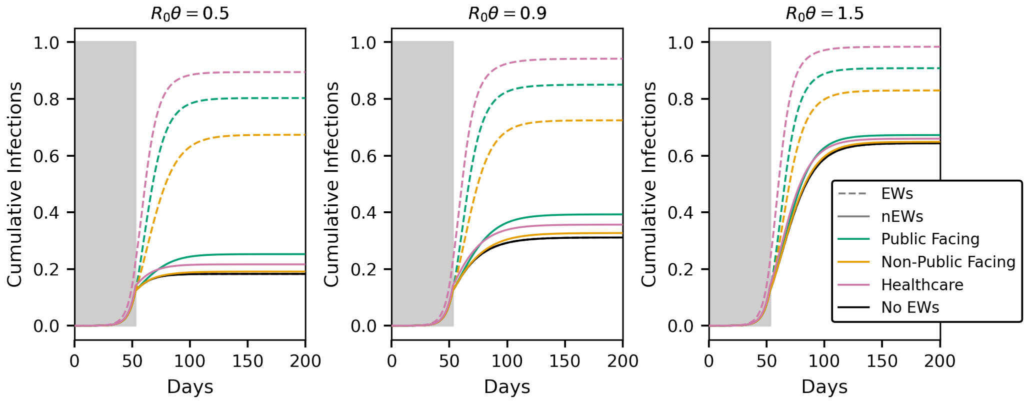

Figure S11. Cumulative infection rates among groups when 50% of EW contacts are with other EWs (i.e., $\rho = 0.5$). The public-facing EWs now have a larger personal risk of infection but low impact on the broader pandemic. This is the non-public-facing and healthcare EW models shown in the main. See Figure 2 for details.

[24]:
```
change_in_percent_hospitalized = 0.5
set_parameters = {'p_IH':0.066*change_in_percent_hospitalized}
plot_grid(theta_values = desired_Reff/R_0,
          set_parameters = set_parameters)
```

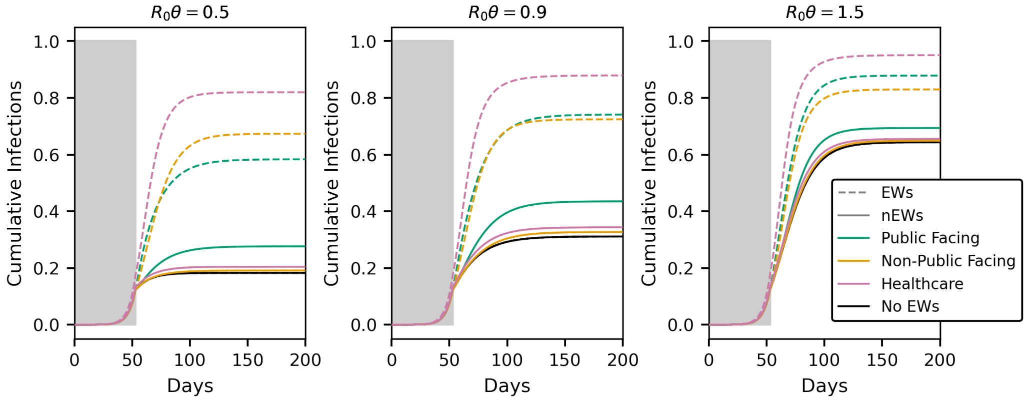

Figure S12. Cumulative infection rates among groups when halving the number of infections requiring hospitalization. This only affects the healthcare EW model. See Figure 2 for details.

[25]:
```
change_in_percent_hospitalized = 2
set_parameters = {'p_IH':0.066*change_in_percent_hospitalized}
plot_grid(theta_values = desired_Reff/R_0,
          set_parameters = set_parameters)
```

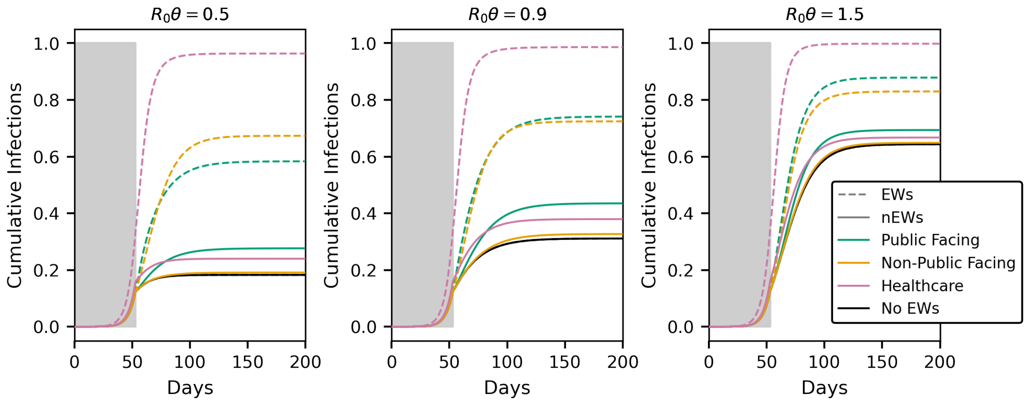

Figure S13. Cumulative infection rates among groups when doubling the number of infections requiring hospitalization. This only affects the healthcare EW model. See Figure 2 for details.

[26]:
```
change_time_hospitalized = 0.5
set_parameters = {'t_HR':8*change_time_hospitalized,
                  't_HC':6*change_time_hospitalized}
plot_grid(theta_values = desired_Reff/R_0,
          set_parameters = set_parameters)
```

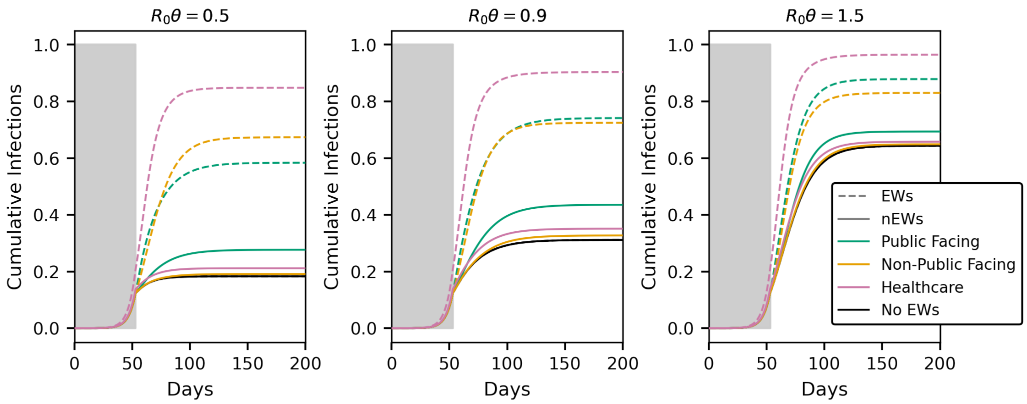

Figure S14. Cumulative infection rates among groups when halving the time spent in the initial hospital compartments. This only affects the healthcare EW model. See Figure 2 for details.

[27]:
```
change_time_hospitalized = 2
set_parameters = {'t_HR':8*change_time_hospitalized,
                  't_HC':6*change_time_hospitalized}
plot_grid(theta_values = desired_Reff/R_0,
          set_parameters = set_parameters)
```

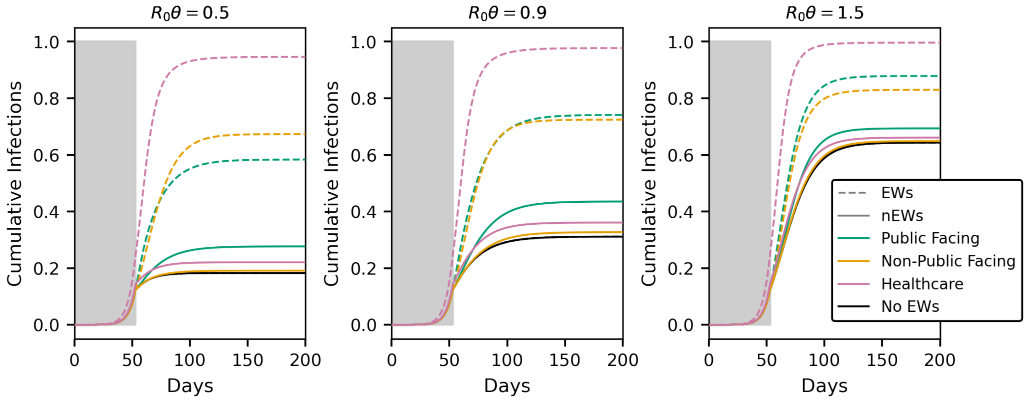

Figure S15. Cumulative infection rates among groups when doubling the time spent in the initial hospital compartments. This only affects the healthcare EW model. See Figure 2 for details.

[28]:
```
include_critical_care = False
plot_grid(theta_values = desired_Reff/R_0)
include_critical_care = True
```

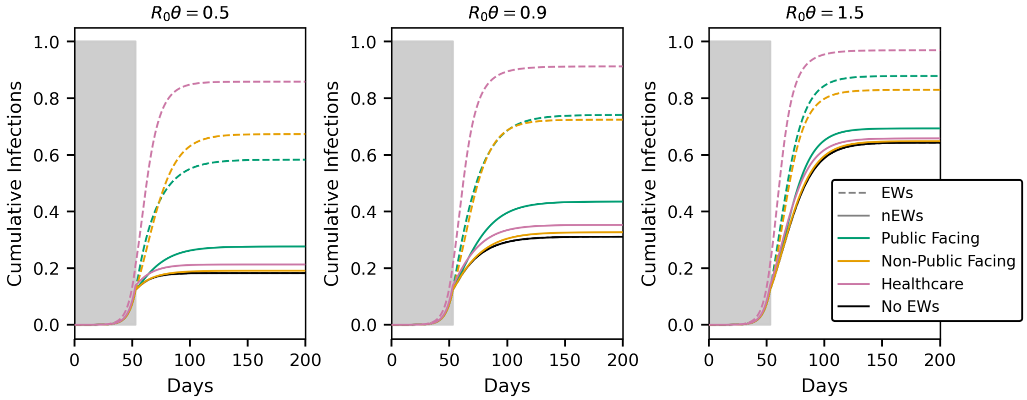

Figure S16. Cumulative infection rates among groups when assuming patients in critical care are not infectious. See Figure 2 for details.

0.1.3 S3 - Heatmaps of cumulative infections

Next, we can plot the cumulative infections after a year in the total population and in each class of worker as a heatmap for combinations of θ and f. To do so, we need to iterate over a grid of θ and f values and solve the model for each combination. The functions below run these iterations and set up the plotting area for the heatmaps:

[29]:
```
# Run several models over different values of theta and f
# Here, we look at theta between 0 and 1, and f between 0 and 0.25
def run_iterations(model_names = ['Cashier','USPS','Healthcare'],
                   rho_values = {'Cashier':0,
                                 'USPS':0.5,
                                 'Healthcare':0.5},
                   theta_values = np.linspace(0,1,21),
                   f_values = np.linspace(0,0.25,26),
                   R_0 = args_default.R_0,
                   t_lockdown = args_default.social_distancing_time,
                   n_infected = 20,
                   n_exposed = 0,
                   max_days = 500,
                   change_in_p_IH = 1,
                   change_in_p_IA = 1,
                   doubling_time = None,
                   adjust_beta_hosp = adjust_beta_hosp,
                   set_parameters = {}):

    results_dict = {}
    # For each of the specified models
    for model in model_names:

        results_dict[model] = {}

        if model == 'USPS' or model == 'Healthcare':
            rho = 0.5
        else:
            rho = 0
        if model in rho_values.keys():
            rho = rho_values[model]

        # For each of the given f values (f = fraction essential)
        for f in f_values:

            if f == 0: continue
            #print('\t',f)
            results_dict[model][f] = {}

            # for each of the given theta values (theta = effectiveness of␣
↪social distancing)
```

```
            for theta in theta_values:

                results_dict[model][f][theta] = run_model(theta = theta,
                                                            R_0 = R_0,
                                                           ␣
 ↪t_lockdown=t_lockdown,
                                                            model=model,
                                                           ␣
 ↪n_infected=n_infected,
                                                            n_exposed=n_exposed,
                                                            prop_essential=f,
                                                            max_days = max_days,
                                                            rho = rho,
                                                            change_in_p_IH =␣
 ↪change_in_p_IH,
                                                            change_in_p_IA =␣
 ↪change_in_p_IA,
                                                            adjust_beta_hosp =␣
 ↪f*adjust_beta_hosp,
                                                            set_parameters =␣
 ↪set_parameters)

    return results_dict

def assign_row_label_figure3(ax,model, pad = 3, xy = (0,0.5), model_labels =␣
 ↪model_labels,font_size = 12):

    # Adds the text to left of the subplot
    ax.annotate(model_labels[model], xy=xy, xytext=(-ax.yaxis.labelpad - pad,␣
 ↪0),
                xycoords=ax.yaxis.label, textcoords='offset points',
                size=font_size, ha='right', va='center',rotation=90)

def assign_column_label_figure3(ax,text,index, pad = 5, xy = (0.
 ↪5,1),font_size=12):
    letter_maps = {0: 'A',
                   1: 'B',
                   2: 'C'}

    label = letter_maps[index] + '. ' + text
    pad = 5 # in points
    ax.annotate(label+'\n', xy=xy, xytext=(0, pad),
                xycoords='axes fraction', textcoords='offset points',
                size=font_size, ha='center', va='baseline')
```

```
def plot_heatmap(sol_dict,
                 Yname=r'$f$',
                 Xname=r'$R_0\theta$',
                 model_names = ['Cashier','USPS','Healthcare'],
                 font_size = 9,
                 center_point = None,
                 figure_title = '',
                 R_0 = args_default.R_0):

    update_font_size(font_size)
    # The models we are plotting heatmaps for
    model_keys = list(sol_dict.keys())
    #
    variable_1_keys = np.sort([i for i in sol_dict[model_keys[0]].keys()])
    variable_2_keys  = np.sort([i for i in␣
↪sol_dict[model_keys[0]][variable_1_keys[0]].keys()])

    fig, axes_all = plt.subplots(len(model_names),3,figsize=[7.5,6],dpi = 300)
    cbar_ax = fig.add_axes([0.85, 0.15, 0.02, 0.7])
    # Only considering 2 metrics
    for metric in range(3):

        # Name of the metrics and levels to plot contour curves for
        metric_names = {0:('Total infections after one year',[center_point]),
                        1:('Infections among nEWs',[center_point]),
                        2:('Infections among EWs',[center_point])}

        metric_name,levels = metric_names[metric]

        # Get the axis for this model
        axes = [row_axes[metric] for row_axes in axes_all]

        # Create a matrix to build the heatmaps from
        sol_array = np.
↪zeros([len(model_keys),len(variable_1_keys),len(variable_2_keys)])

        for i,model in enumerate(model_names):

            # Reformat keys to look nice
            axis_1_keys = []
            axis_2_keys = []


            for index,key1 in enumerate(variable_1_keys):
                axis_1_keys.append(round(R_0*(key1),2))

            for index,key2 in enumerate(variable_2_keys):
```

```
            axis_2_keys.append(key2)

        # Fill in the matrix
        model_dict = sol_dict[model]
        for j,(var1,var1_dict) in enumerate(model_dict.items()):
            for k,(var2,solutions) in enumerate(var1_dict.items()):
                sol_array[i,j,k] = solutions[metric]

        plt.sca(axes[i])
        # Get the relevant part of the matrix and orientate the matrix␣
↪correctly (tranpose then flip vertically)
        data = np.flip(np.transpose(sol_array[i,:,:]))
        data = pd.DataFrame(data)
        data.index = np.flip(axis_2_keys)
        data.columns = np.flip(axis_1_keys)
        # Make a heatmap from it
        HM = heatmap(data=data,
                     yticklabels=5,xticklabels=5,
                     cmap = 'Spectral',
                     center = center_point,
                     vmin = 0,
                     vmax = 1,
                     cbar_ax=cbar_ax)

        CS = plt.contour(data,levels=levels,colors='r',linewidths=2)
        plt.yticks(rotation=0)
        axes[i].tick_params(width = 1,length = 2)

        # Label heatmaps with model names
        model_labels = {'Cashier'               :'Public Facing',
                        'USPS'                  :'Non-Public Facing',
                        'Healthcare'            :'Healthcare',
                        'No Structure'          :'No essential workers',
                        'Healthcare_Sigmoid'    :'Healthcare'}

        if i == 0:
            assign_column_label_figure3(ax=axes[i],text = metric_name,␣
↪index = metric,font_size=font_size)
        if metric == 0:
            assign_row_label_figure3(ax=axes[i],model =␣
↪model,font_size=font_size)

        # Set variable names on the axis
        if Yname:
            axes[i].set_ylabel(Yname,labelpad=0)
        if Xname:
            axes[i].set_xlabel(Xname)
```

```
    cbar_ax.tick_params(width = 1,length = 2)
    fig.subplots_adjust(hspace=0.35,wspace=0.4,right = 0.825)
    fig.suptitle(figure_title)
    plt.show()
```

The following block of code reproduces the heatmaps depicted in Figure 3 of the main text. Here, we are assuming a lockdown time of 47 days.

[30]:
```
R_0_values = np.linspace(2,0,21)
theta_values=np.sort(R_0_values/R_0)
all_results_phaseDiagram2 = run_iterations(theta_values=theta_values)
solved_results = get_metrics(all_results_phaseDiagram2)

# This plots the metrics we want as a heatmap.
sol, pp = run_model(t_lockdown=args_default.social_distancing_time,
                    theta = (1/R_0),
                    R_0 = R_0,
                    n_exposed = 0,
                    n_infected = 20,
                    rho = 0,
                    model = 'No Structure',
                    prop_essential=0.05)
center_point = get_infection_rate(solution=sol,p=pp, time = 365)
```

[31]:
```
plot_heatmap(solved_results,
             center_point = center_point)
```

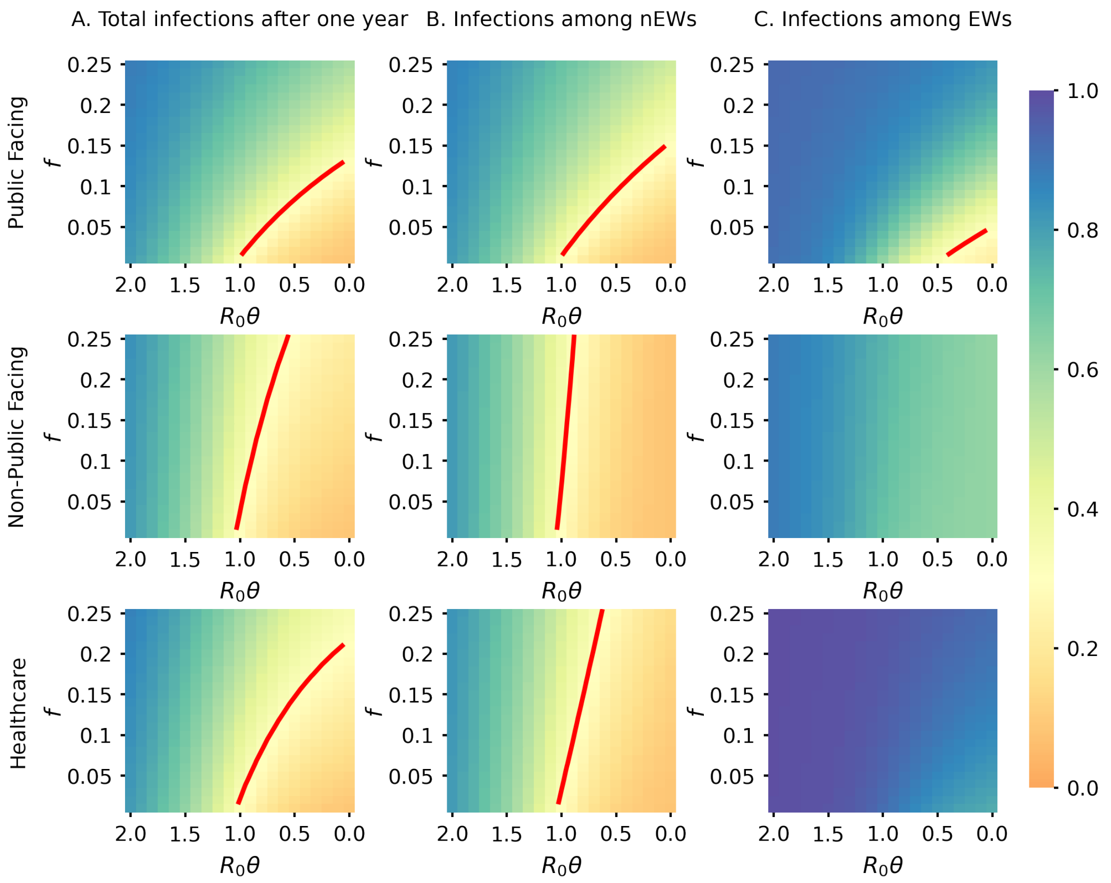

Alternative versions of figure 3 with different parameter values We reproduce figure 3 while varying individual parameters or relaxing model assumptions.

[32]:
```
R_0_values = np.linspace(2,0,21)
theta_values=np.sort(R_0_values/R_0)
all_results_phaseDiagram2 = run_iterations(theta_values=theta_values,
                                           t_lockdown = args_default.
 ↪social_distancing_time - 10)
solved_results = get_metrics(all_results_phaseDiagram2)

# This plots the metrics we want as a heatmap.
sol, pp = run_model(t_lockdown=args_default.social_distancing_time-10,theta =␣
 ↪(1/R_0))
center_point = get_infection_rate(solution=sol,p=pp, time = 365)

plot_heatmap(solved_results,
             center_point = center_point)
```

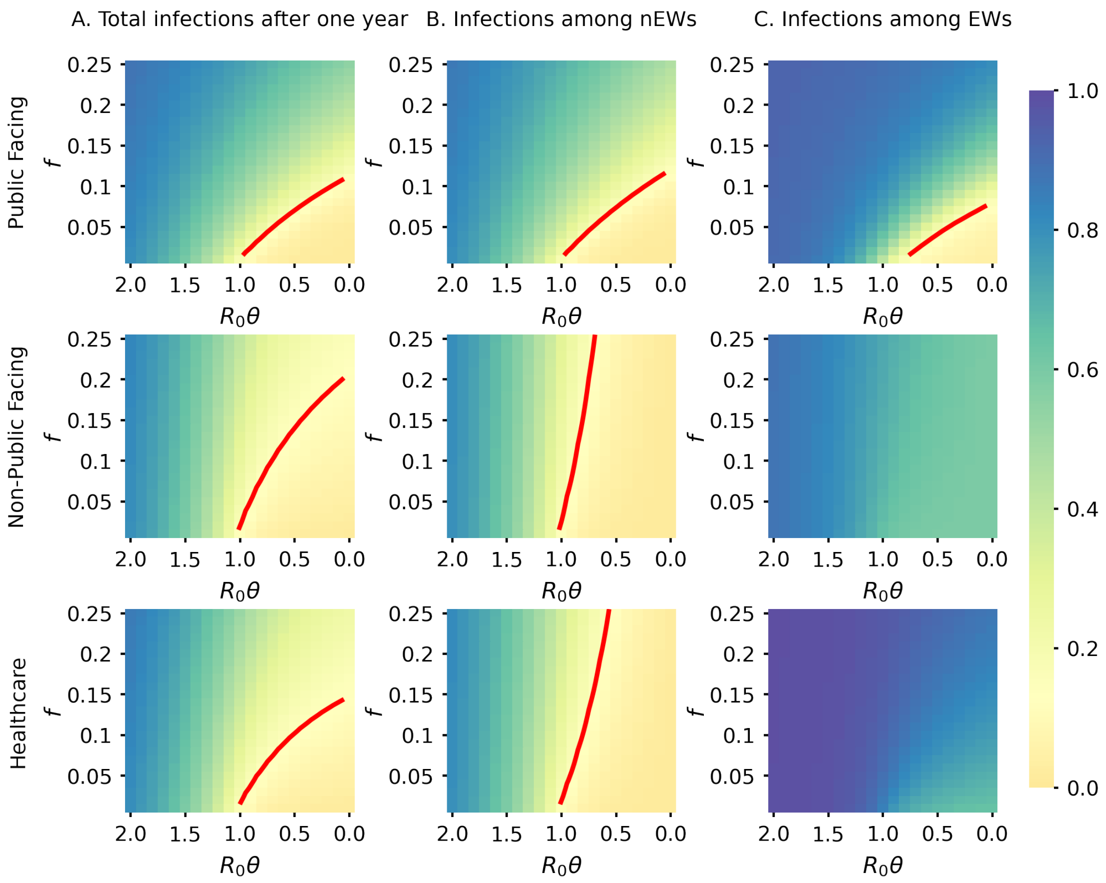

Figure S17. Heatmaps of cumulative infections when lockdown is ten days earlier. See Figure 3 for details.

[33]:
```
R_0 = 2.5

R_0_values = np.linspace(2,0,21)
theta_values=np.sort(R_0_values/R_0)
all_results_phaseDiagram2 = run_iterations(theta_values=theta_values,
                                          R_0 = R_0)
solved_results = get_metrics(all_results_phaseDiagram2)

# This plots the metrics we want as a heatmap.
sol, pp = run_model(R_0 = R_0,theta = (1/R_0))
center_point = get_infection_rate(solution=sol,p=pp, time = 365)

plot_heatmap(solved_results,
             center_point = center_point,
             R_0 = R_0)
```

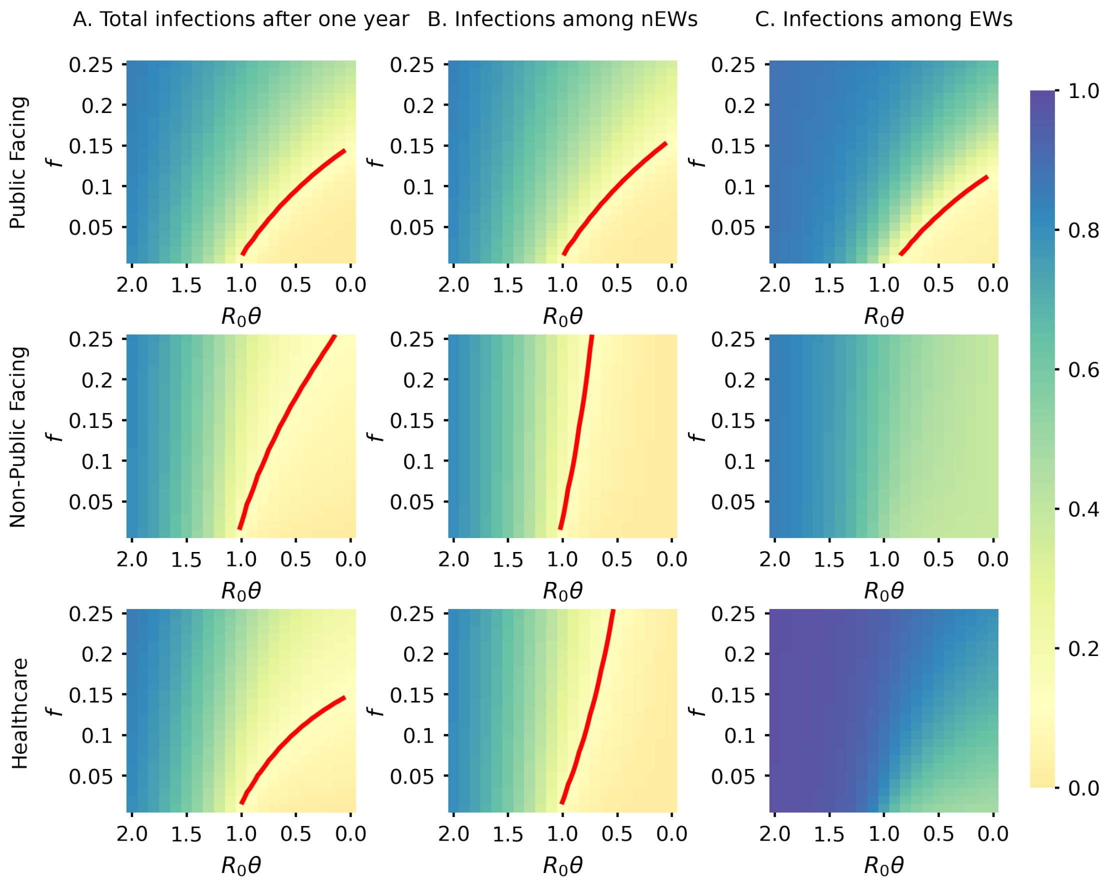

Figure S18. Heatmaps of cumulative infections when $R_0 = 2.5$. See Figure 3 for details.

[34]:
```
R_0 = 3.5

R_0_values = np.linspace(2,0,21)
theta_values=np.sort(R_0_values/R_0)
all_results_phaseDiagram2 = run_iterations(theta_values=theta_values,
                                           R_0 = R_0)
solved_results = get_metrics(all_results_phaseDiagram2)

# This plots the metrics we want as a heatmap.
sol, pp = run_model(R_0 = R_0,theta = (1/R_0))
center_point = get_infection_rate(solution=sol,p=pp, time = 365)

plot_heatmap(solved_results,
             center_point = center_point,
             R_0 = R_0)

R_0 = args_default.R_0
```

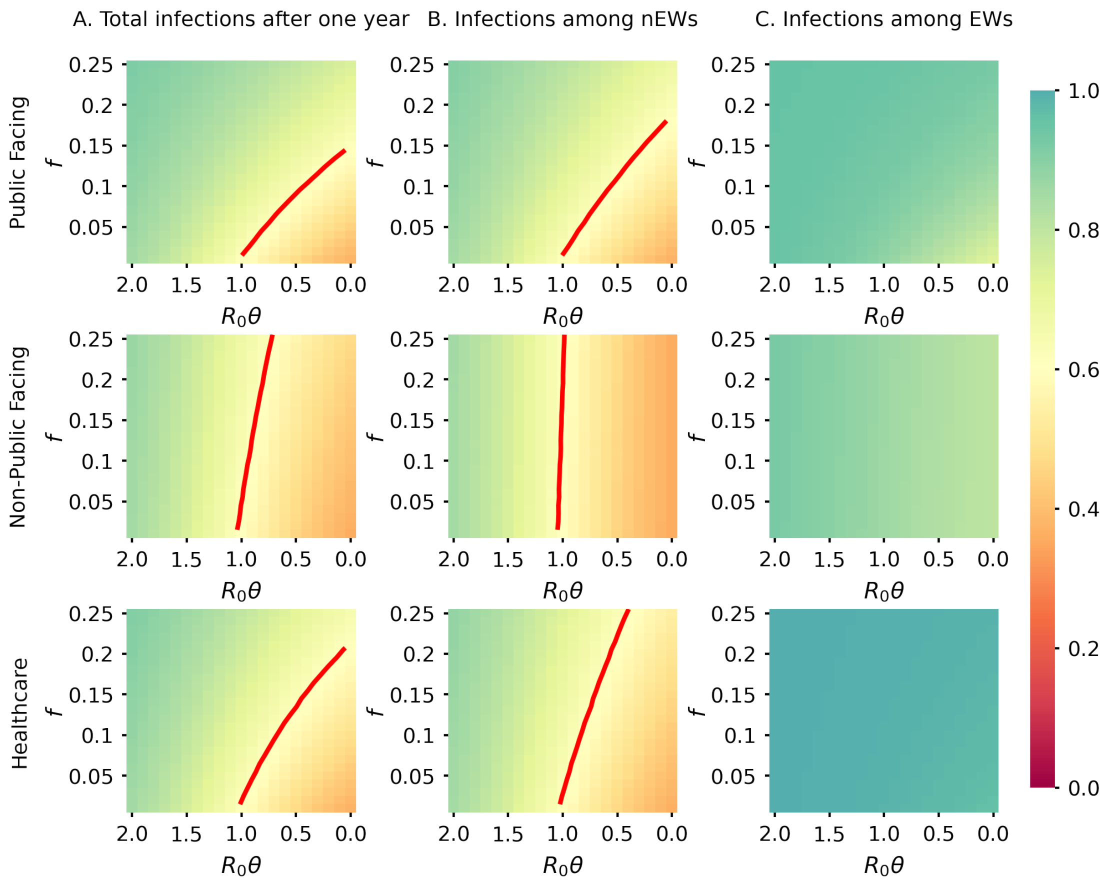

Figure S19. Heatmaps of cumulative infections when $R_0 = 3.5$. See Figure 3 for details.

[35]:
```
change_in_incubation_time = -1
set_parameters = {'t_inc':3+change_in_incubation_time}

R_0_values = np.linspace(2,0,21)
theta_values=np.sort(R_0_values/R_0)
all_results_phaseDiagram2 = run_iterations(theta_values=theta_values,
                                           set_parameters = set_parameters)
solved_results = get_metrics(all_results_phaseDiagram2)

# This plots the metrics we want as a heatmap.
sol, pp = run_model(set_parameters = set_parameters,theta = (1/R_0))
center_point = get_infection_rate(solution=sol,p=pp, time = 365)

plot_heatmap(solved_results,
             center_point = center_point)
```

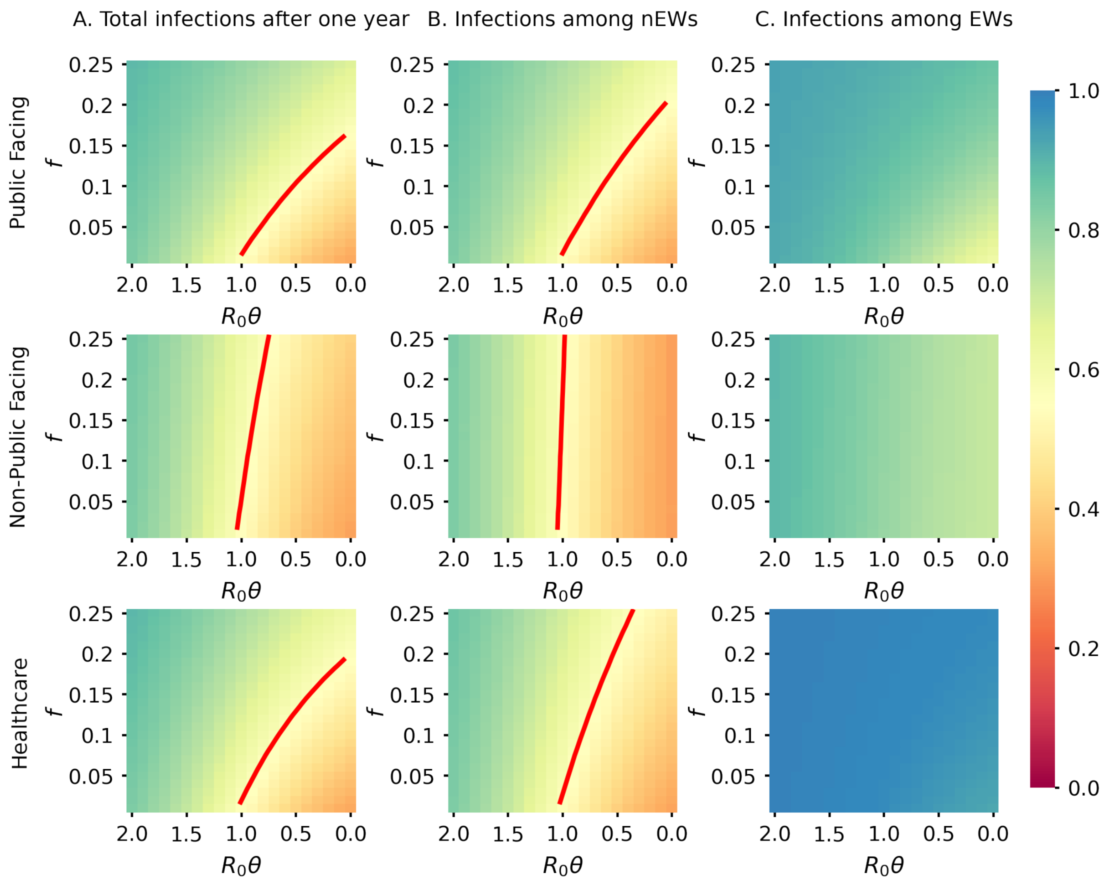

Figure S20. Heatmaps of cumulative infections when $t_E = 2$. See Figure 3 for details.

[36]:
```
change_in_incubation_time = 1
set_parameters = {'t_inc':3+change_in_incubation_time}

R_0_values = np.linspace(2,0,21)
theta_values=np.sort(R_0_values/R_0)
all_results_phaseDiagram2 = run_iterations(theta_values=theta_values,
                                           set_parameters = set_parameters)
solved_results = get_metrics(all_results_phaseDiagram2)

# This plots the metrics we want as a heatmap.
sol, pp = run_model(set_parameters = set_parameters,theta = (1/R_0))
center_point = get_infection_rate(solution=sol,p=pp, time = 365)

plot_heatmap(solved_results,
             center_point = center_point)
```

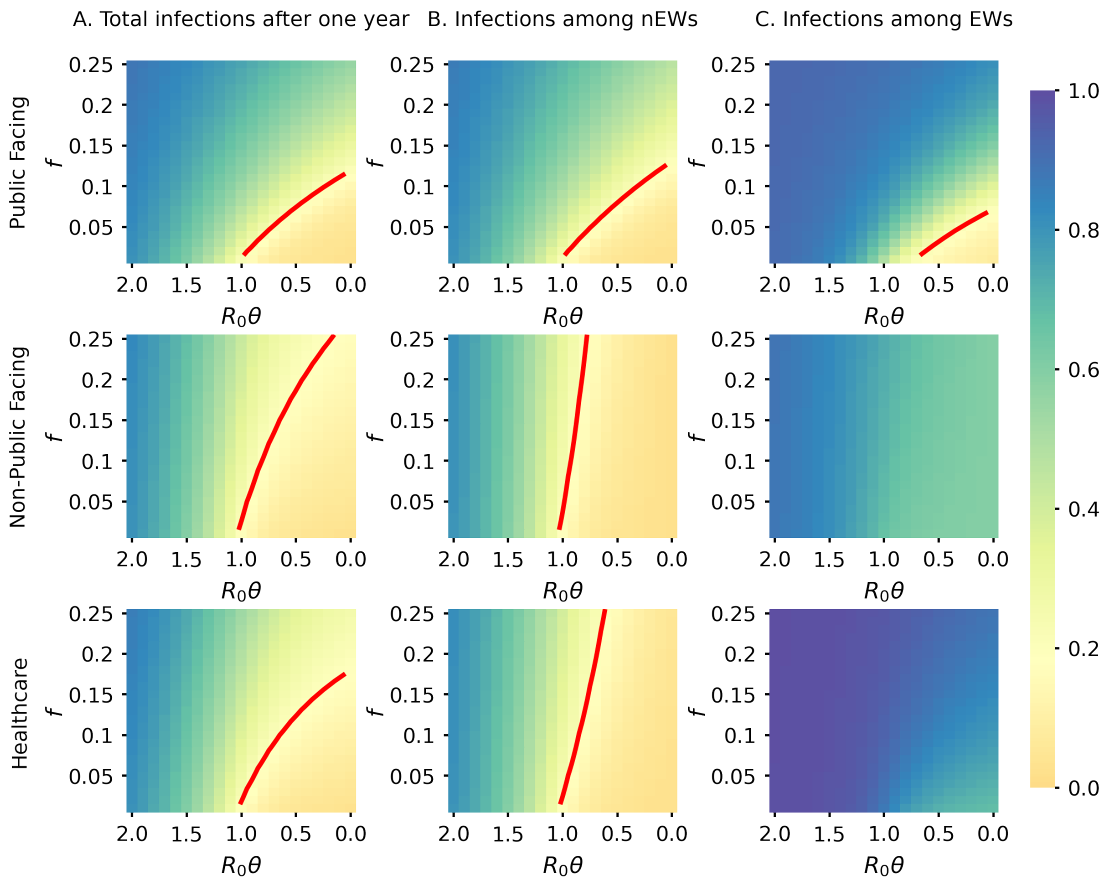

Figure S21. Heatmaps of cumulative infections when $t_E = 4$. See Figure 3 for details.

[37]:
```
increase_time_infectious = 5
set_parameters = {'t_AR':5 + increase_time_infectious,
                  't_IR':5 + increase_time_infectious,
                  't_IH':5 + increase_time_infectious}

R_0_values = np.linspace(2,0,21)
theta_values=np.sort(R_0_values/R_0)
all_results_phaseDiagram2 = run_iterations(theta_values=theta_values,
                                           set_parameters = set_parameters)
solved_results = get_metrics(all_results_phaseDiagram2)

# This plots the metrics we want as a heatmap.
sol, pp = run_model(set_parameters = set_parameters,theta = (1/R_0))
center_point = get_infection_rate(solution=sol,p=pp, time = 365)

plot_heatmap(solved_results,
             center_point = center_point)
```

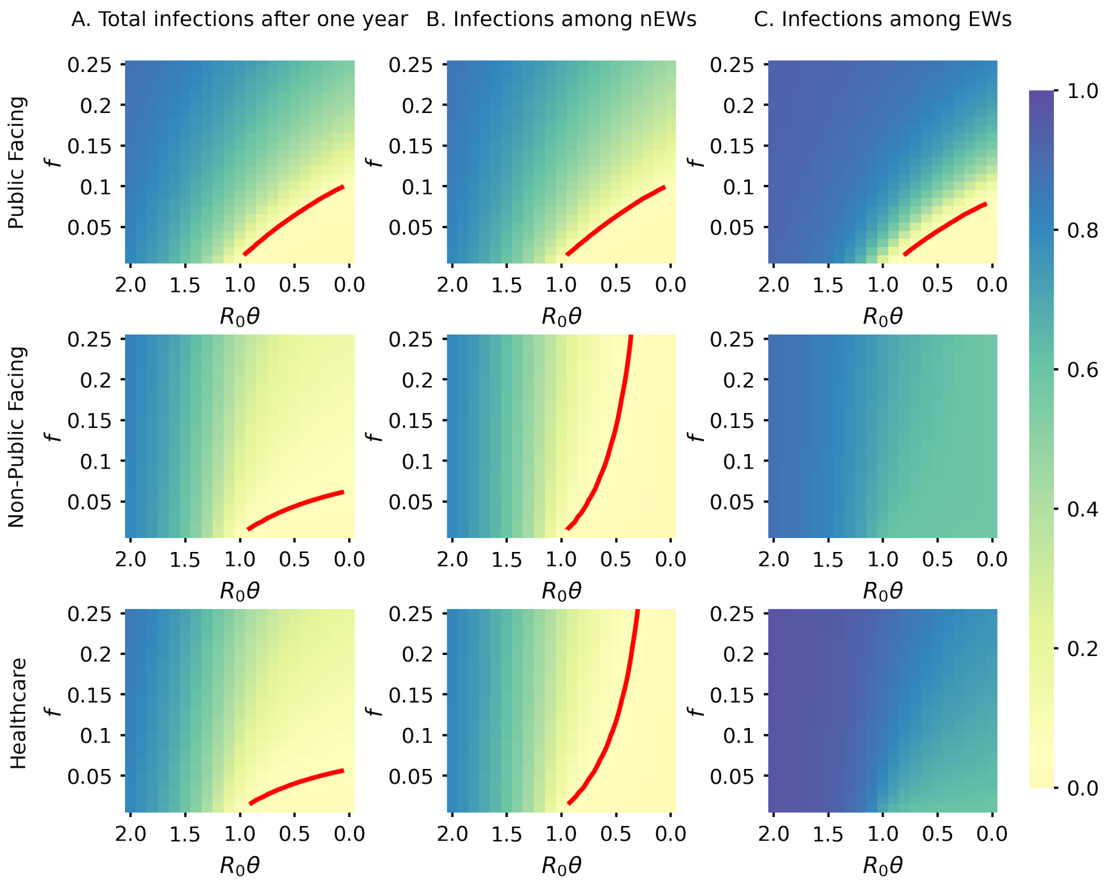

Figure S22. Heatmaps of cumulative infections when the infectious period is five days longer (i.e., $t_{IA} = t_{IR} = t_{IH} = 10$). See Figure 3 for details.

[38]:
```
rho = 0
R_0_values = np.linspace(2,0,21)
theta_values=np.sort(R_0_values/R_0)
all_results_phaseDiagram2 = run_iterations(theta_values=theta_values,
                                           rho_values =  {'Cashier':rho,
                                                          'Healthcare':rho,
                                                          'USPS': rho})
solved_results = get_metrics(all_results_phaseDiagram2)

# This plots the metrics we want as a heatmap.
sol, pp = run_model(rho=rho,theta = (1/R_0))
center_point = get_infection_rate(solution=sol,p=pp, time = 365)

plot_heatmap(solved_results,
             center_point = center_point)
```

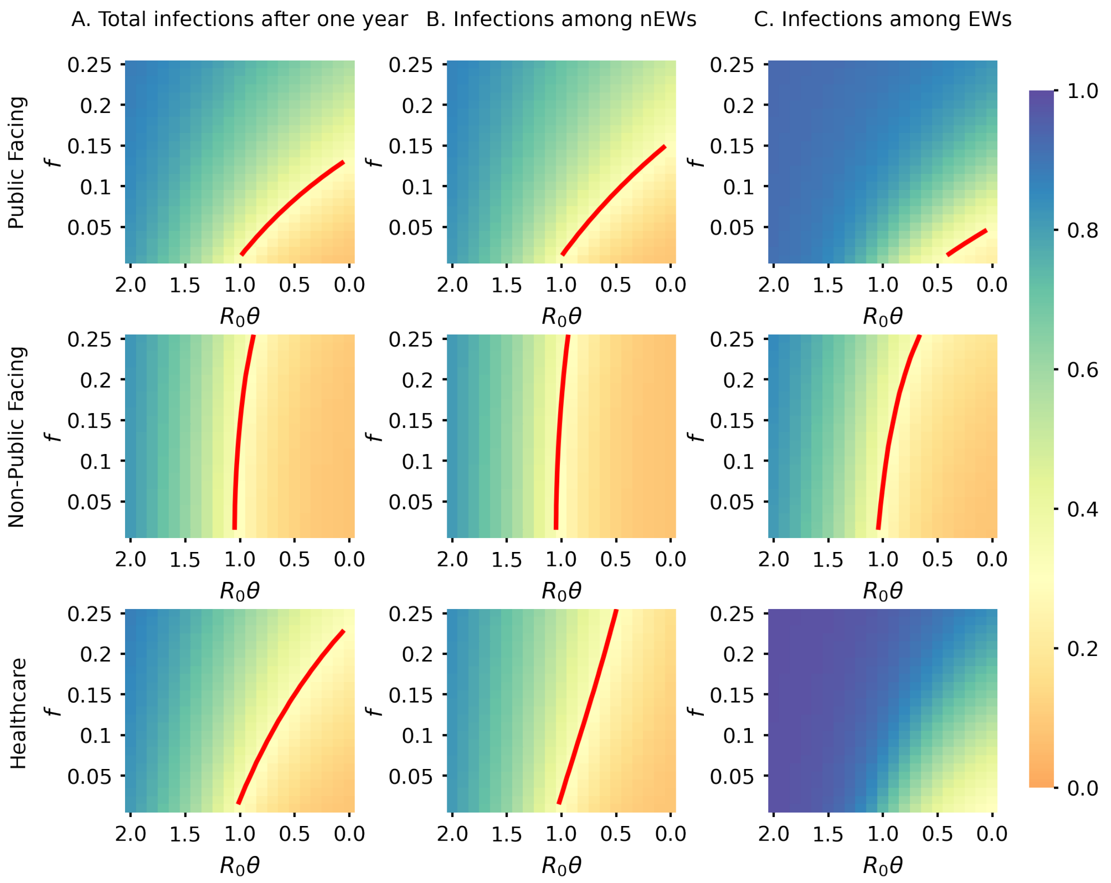

Figure S23. Heatmaps of cumulative infections when all contacts are distributed proportionally (i.e., $\rho = 0$). In the non-public facing model, only 2.5% of contacts are not affected by SIP so EWs have little impact on the broader pandemic. The healthcare EW model remains qualitatively similar. The public-facing EW model shown here is the same as that shown in the main. See Figure 3 for details.

[39]:
```
rho = 0.5
R_0_values = np.linspace(2,0,21)
theta_values=np.sort(R_0_values/R_0)
all_results_phaseDiagram2 = run_iterations(theta_values=theta_values,
                                           rho_values =  {'Cashier':rho,
                                                          'Healthcare':rho,
                                                          'USPS': rho})
solved_results = get_metrics(all_results_phaseDiagram2)

# This plots the metrics we want as a heatmap.
sol, pp = run_model(rho=rho,theta = (1/R_0))
center_point = get_infection_rate(solution=sol,p=pp, time = 365)
```

```
plot_heatmap(solved_results,
             center_point = center_point)
```

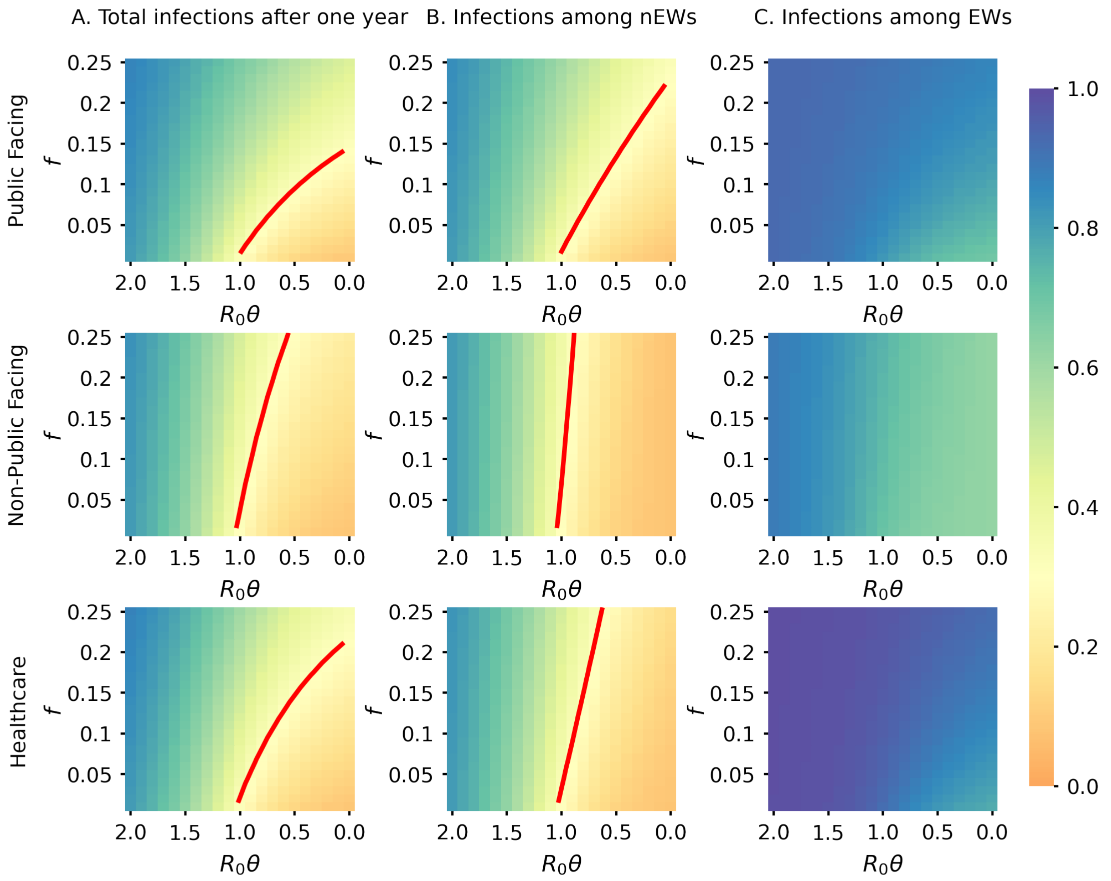

Figure S24. Heatmaps of cumulative infections when 50% of EW contacts are with other EWs (i.e., $\rho = 0.5$). The public-facing EWs now have a larger personal risk of infection but low impact on the broader pandemic. The non-public-facing EW and healthcare EW model shown here are unchanged from those shown in the main. See Figure 3 for details.

[40]:
```
change_in_percent_hospitalized = 2
set_parameters = {'p_IH':0.066*change_in_percent_hospitalized}

R_0_values = np.linspace(2,0,21)
theta_values=np.sort(R_0_values/R_0)
all_results_phaseDiagram2 = run_iterations(theta_values=theta_values,
                                            set_parameters = set_parameters)
solved_results = get_metrics(all_results_phaseDiagram2)

# This plots the metrics we want as a heatmap.
sol, pp = run_model(set_parameters = set_parameters,theta = (1/R_0))
```

```
center_point = get_infection_rate(solution=sol,p=pp, time = 365)

plot_heatmap(solved_results,
             center_point = center_point)
```

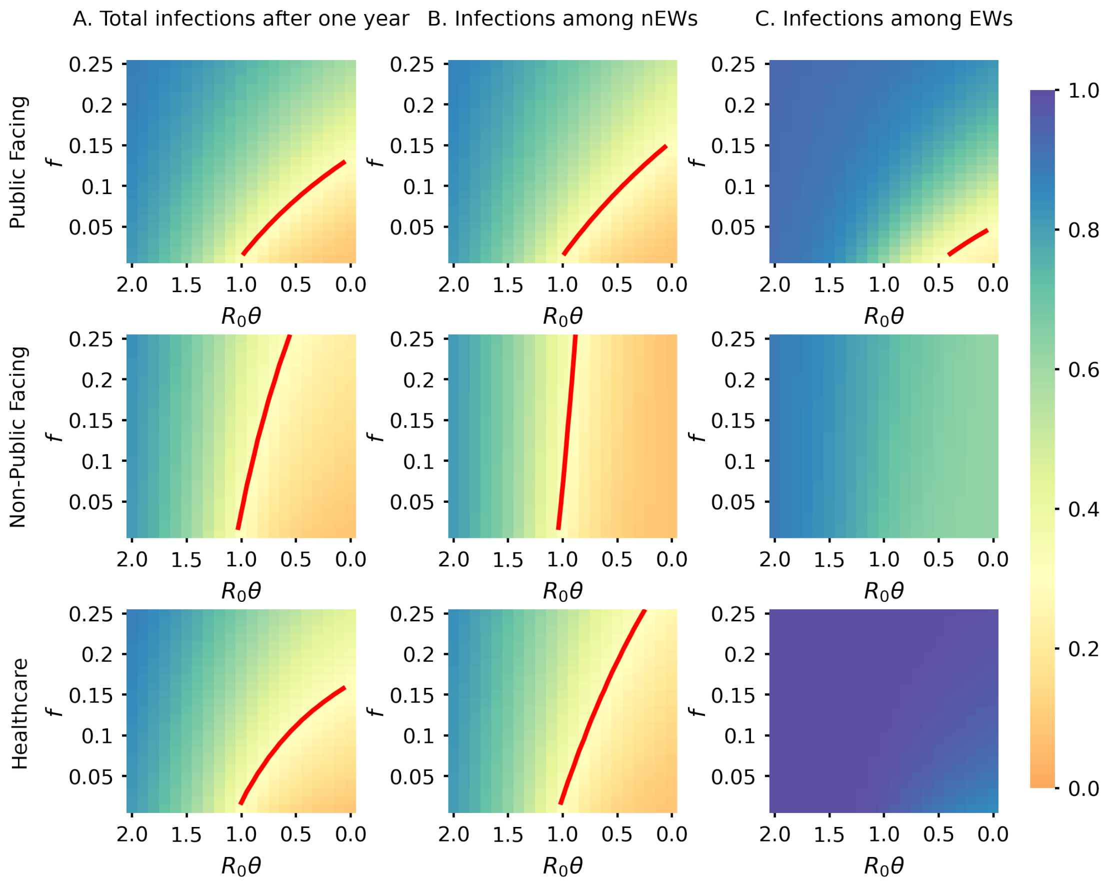

Figure S25. Heatmaps of cumulative infections when percent of infections requiring hospitalization doubles. This parameter only affects the healthcare EW model. See Figure 3 for details.

[41]:
```
change_in_percent_hospitalized = 0.5
set_parameters = {'p_IH':0.066*change_in_percent_hospitalized}

R_0_values = np.linspace(2,0,21)
theta_values=np.sort(R_0_values/R_0)
all_results_phaseDiagram2 = run_iterations(theta_values=theta_values,
                                            set_parameters = set_parameters)
solved_results = get_metrics(all_results_phaseDiagram2)

# This plots the metrics we want as a heatmap.
sol, pp = run_model(set_parameters = set_parameters,theta = (1/R_0))
```

```
center_point = get_infection_rate(solution=sol,p=pp, time = 365)

plot_heatmap(solved_results,
             center_point = center_point)
```

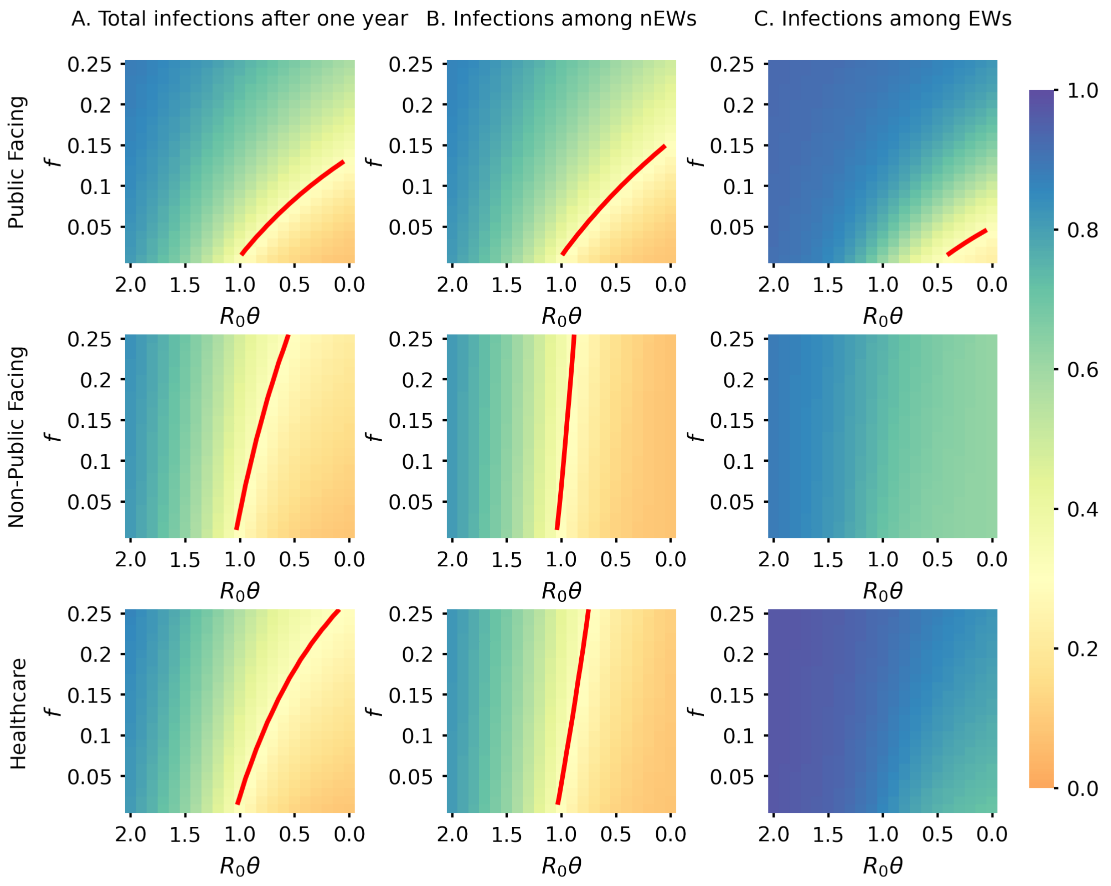

Figure S26. Heatmaps of cumulative infections when percent of infections requiring hospitalization is halved. This parameter only affects the healthcare EW model. See Figure 3 for details.

[42]:
```
change_time_hospitalized = 2
set_parameters = {'t_HR':8*change_time_hospitalized,
                  't_HC':6*change_time_hospitalized}

R_0_values = np.linspace(2,0,21)
theta_values=np.sort(R_0_values/R_0)
all_results_phaseDiagram2 = run_iterations(theta_values=theta_values,
                                           set_parameters = set_parameters)
solved_results = get_metrics(all_results_phaseDiagram2)

# This plots the metrics we want as a heatmap.
```

```
sol, pp = run_model(set_parameters = set_parameters,theta = (1/R_0))
center_point = get_infection_rate(solution=sol,p=pp, time = 365)

plot_heatmap(solved_results,
             center_point = center_point)
```

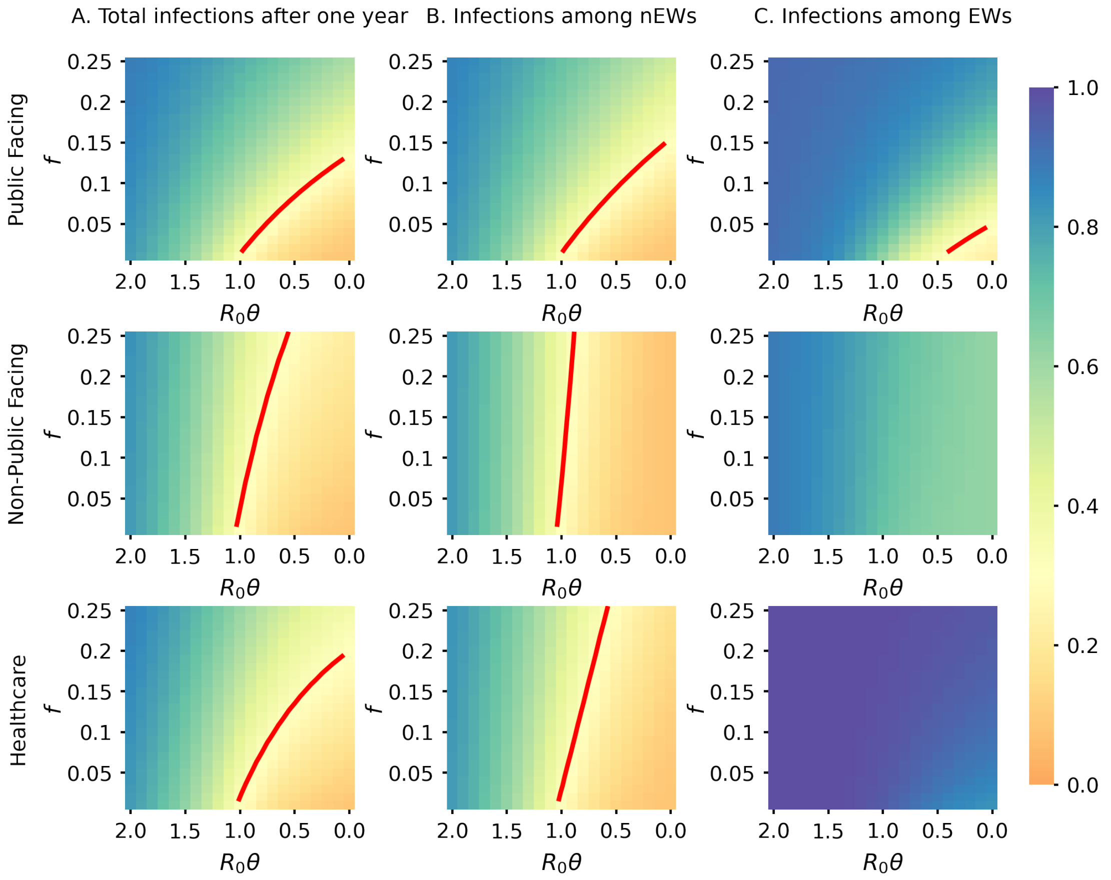

Figure S27. Heatmaps of cumulative infections when time spent in initial hospital compartment is doubled. This parameter only affects the healthcare EW model. See Figure 3 for details.

[43]:
```
change_time_hospitalized = 0.5
set_parameters = {'t_HR':8*change_time_hospitalized,
                  't_HC':6*change_time_hospitalized}

R_0_values = np.linspace(2,0,21)
theta_values=np.sort(R_0_values/R_0)
all_results_phaseDiagram2 = run_iterations(theta_values=theta_values,
                                           set_parameters = set_parameters)
solved_results = get_metrics(all_results_phaseDiagram2)
```

```
# This plots the metrics we want as a heatmap.
sol, pp = run_model(set_parameters = set_parameters,theta = (1/R_0))
center_point = get_infection_rate(solution=sol,p=pp, time = 365)

plot_heatmap(solved_results,
             center_point = center_point)
```

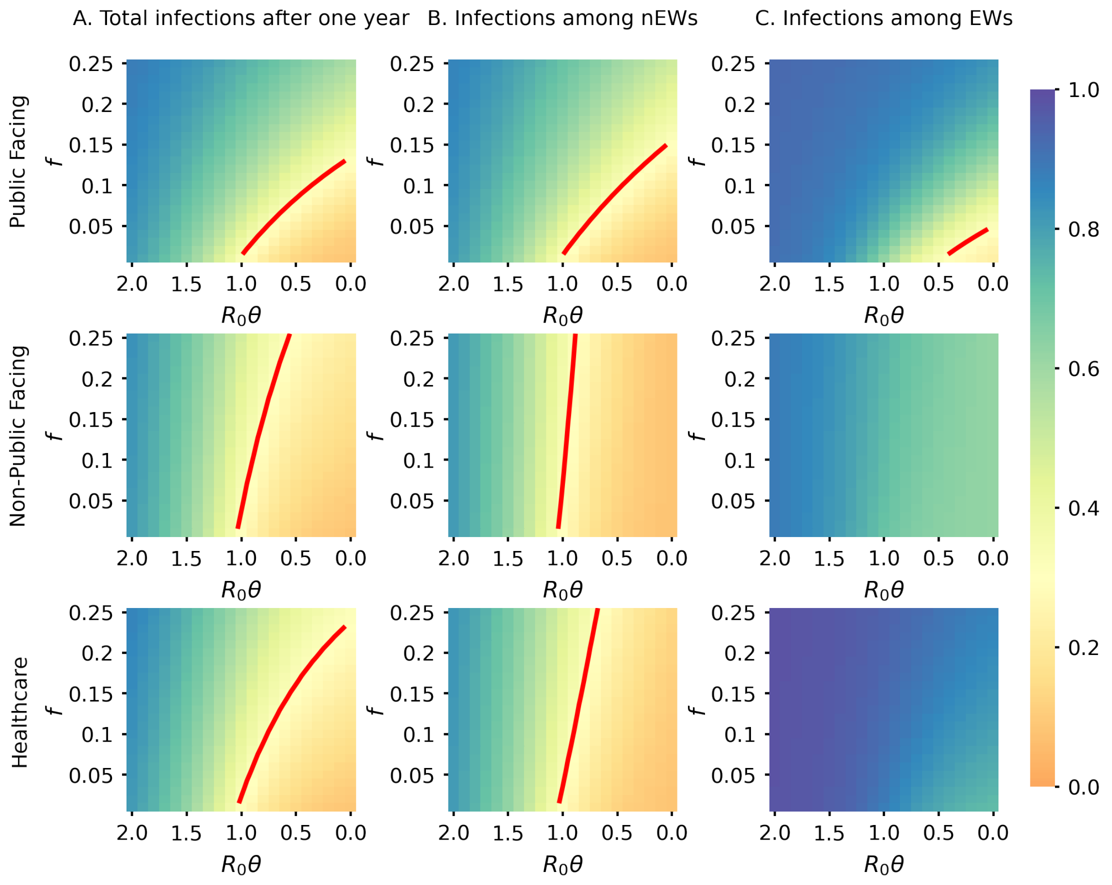

Figure S28. Heatmaps of cumulative infection when time spend in the initial hospital compartments is halved. This parameter only affects the healthcare EW model. See Figure 3 for details.

[44]:
```
include_critical_care = False
R_0_values = np.linspace(2,0,21)
theta_values=np.sort(R_0_values/R_0)
all_results_phaseDiagram2 = run_iterations(theta_values=theta_values)
solved_results = get_metrics(all_results_phaseDiagram2)

# This plots the metrics we want as a heatmap.
sol, pp = run_model(theta = (1/R_0))
center_point = get_infection_rate(solution=sol,p=pp, time = 365)
```

```
plot_heatmap(solved_results,
             center_point = center_point)
```

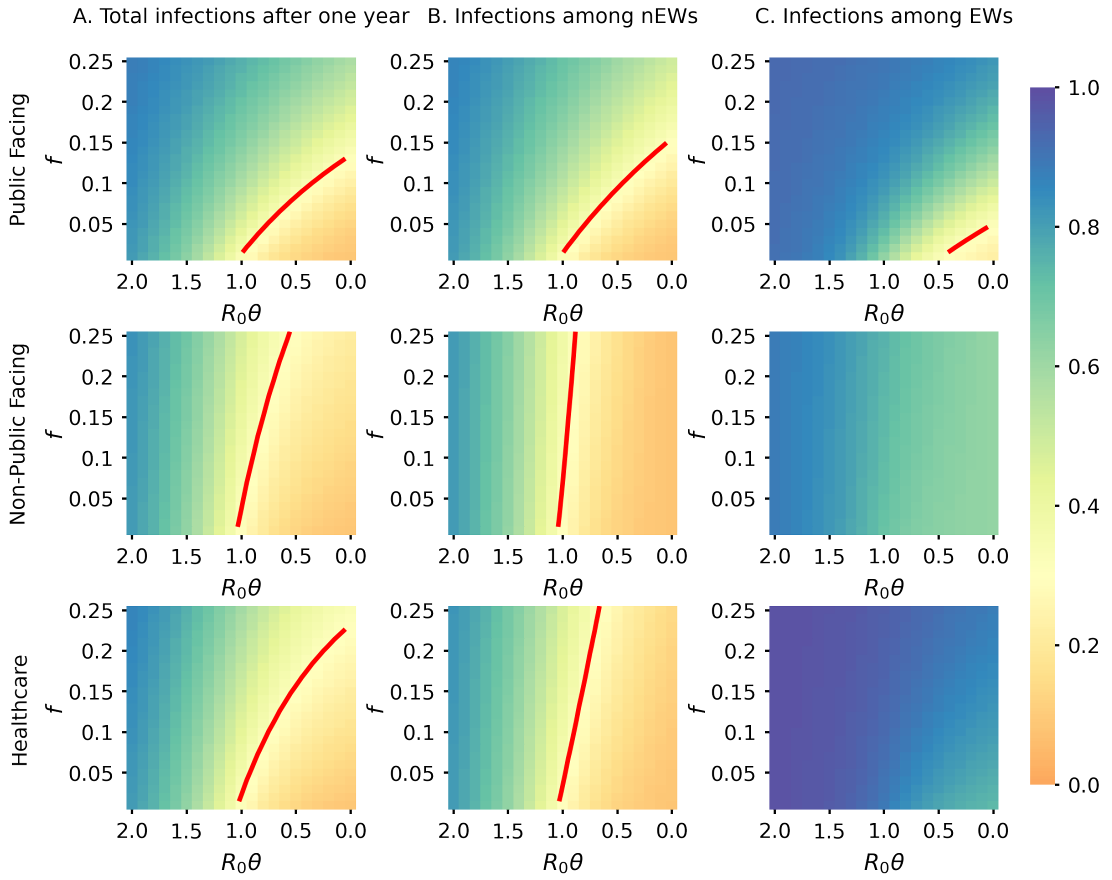

Figure S29. Heatmaps of cumulative infections when patients in critical care are not considered infectious. This parameter only affects the healthcare EW model. See Figure 3 for details.

0.1.4 S4 - Time-resolved dynamics of infections

In Figure 4 of the main text, we show the time-resolved dynamics of infections for $f = 0.05$ and $R_0\theta = 0.5$. The following code defines functions to calculate the time-resolved proportion of essential and non-essentials that are infected, the fraction of infected individuals that are essential workers, and the proportion of new infections for different routes of transmission.

[45]:
```
# Assign row and column labels for the plots of the effective R over time
def assign_row_label_figure4(ax,model,letter,pad = 0, xy = (0,0.5),letter_pad =␣
↪0):
    if not letter_pad:
        letter_pad = pad*20
    ax.annotate(letter, xy=xy, xytext=(-ax.yaxis.labelpad - letter_pad, 0),
```

```
                xycoords=ax.yaxis.label, textcoords='offset points',
                size='large', ha='center', va='center',rotation=0)

    ax.annotate(model+'\n', xy=xy, xytext=(-ax.yaxis.labelpad - pad, 0),
                xycoords=ax.yaxis.label, textcoords='offset points',
                size='large', ha='center', va='center',rotation=90)

# Label is the value of R_effective
def assign_column_label_figure4(ax,model, pad = 5, xy = (0.5,1)):
    text = model_labels[model]
    pad = 5 # in points
    ax.annotate(text+'\n', xy=xy, xytext=(0, pad),
                xycoords='axes fraction', textcoords='offset points',
                size='large', ha='center', va='baseline')

# Get the three metrics to plot in Figure 4
def get_figure4_metrics(solution_matrix,p):

    # Fraction of population an essential worker
    f     = p.proportion_essential

    # Proportion infected in each group
    I_t_0   = sum(solution_matrix[0,2:5,:])
    I_t_1   = sum(solution_matrix[1,2:5,:])

    # G(t): proportion of infected that are essential workers
    G_t = (I_t_1)/(I_t_0+I_t_1)

    # Find the effects of social distancing. Second variable is from a␣
↪different implementation
    social_distancing_effect = get_effect_of_social_distancing(p,None)

    # beta is R_0 * mean infectious time
    # This is the same beta across all infectious compartments and groups
    nointervention_beta = get_infectious_beta(p)

    # Get the within hospital beta and calculate number of within hospital␣
↪infections. 0 if not a healthcare model
    if 'Healthcare' in p.model:
        hosp_beta = get_hosp_beta(t='None',p=p,H='None')
    else:
        hosp_beta = 0
    within_hosp_infections = hosp_beta * sum(sum(solution_matrix[:,5:10,:])) *␣
↪solution_matrix[1,0,:] / (p.N*f)

    t = np.arange(p.max_days)
    new_infections = np.zeros([2,2,p.max_days])
```

```
    people_distribution = [1-f,f]
    for j in t:

        # Calculate betas. This is elementwise multiplication not matrix␣
↪multiplication
        betas = nointervention_beta*(p.C)
        if j>p.social_distancing_time:
            betas = betas*(social_distancing_effect)

        # Include extra effect from within group mixing
        betas[1,1] += get_extra_rho_effect(j,p,1) * nointervention_beta

        for i in [0,1]:
            for k in [0,1]:
                new_infections[i,k,j] = betas[k,i] * sum(solution_matrix[i,2:
↪5,j]) * (solution_matrix[k,0,j]) / (p.N*people_distribution[k])

        total_infections = sum(sum(new_infections[:,:
↪,j]))+within_hosp_infections[j]
        new_infections[:,:,j] = new_infections[:,:,j]/total_infections
        within_hosp_infections[j] = within_hosp_infections[j]/total_infections
    return G_t, (I_t_0/(p.N*(1-f)), I_t_1/(p.N*f)),␣
↪new_infections,within_hosp_infections

# Make model labels a global so we can access anywhere
model_labels = {'Cashier'         :'Public Facing',
                'USPS'        :'Non-Public Facing',
                'Healthcare'  :'Healthcare',
                'No Structure':'No essential workers',
                'Healthcare_Sigmoid'  :'Healthcare'}

# Function to plot the metrics
def create_figure4(desired_Reff = 0.5,
                   models = ['Cashier','USPS','Healthcare'],
                   t_lockdown = args_default.social_distancing_time,
                   max_days = 500,
                   R_0=args_default.R_0,
                   I0=20,
                   adjust_beta_hosp = adjust_beta_hosp,
                   linewidth = 1,
                   xlim = 250,
                   legend_keywords = {'framealpha': 1,
                                      'edgecolor':'k'},
                   figure_title = '',
                   set_parameters = {},
                   f = 0.05,
                   model_dependent_rho = {'No Structure': 0,
```

```
                'Cashier': 0,
                'Healthcare': 0.5,
                'USPS': 0.5,
                'Healthcare_Sigmoid':  0}):

theta = desired_Reff/R_0
model_dependent_f = {'No Structure':       f,
                     'Cashier':            f,
                     'USPS':               f,
                     'Healthcare':         f,
                     'Healthcare_Sigmoid': f}

fig,  axes_all = plt.subplots(3, len(models),figsize=(7.5,7.5),dpi=300)
(axes_I,axes_G,axes_B) = axes_all
# Plotting LS and color for each model

ls_styles = {'No Structure':('-',[0,0,0,0.5]),
             'Cashier':(':',[0,0,1,1]),
             'Healthcare':('--','r'),
             'USPS':('-.','g'),
             'Healthcare_Sigmoid':('-.','purple')}

legend_entries = {}
max_values_r = ddict(float)
max_values_g = ddict(float)
for model_index,model in enumerate(models):

        # If doing multiple theta values, unpack axes
        if len(models) > 1:
            ax_G = axes_G[model_index]
            ax_I = axes_I[model_index]
            ax_B = axes_B[model_index]
        else:
            ax_G = axes_G
            ax_I = axes_I
            ax_B = axes_B

        # Get f and rho for each model
        f1 = model_dependent_f[model]
        rho1 = model_dependent_rho[model]

        # run model and reshape output
        solution,p = run_model(theta = theta,
                               R_0 = R_0,
                               t_lockdown = t_lockdown,
                               model = model,
                               n_infected = I0,
```

```
                                     n_exposed  = 0,
                                     prop_essential = f1,
                                     max_days = max_days,
                                     rho = rho1,
                                     adjust_beta_hosp = adjust_beta_hosp*f1,
                                     set_parameters=set_parameters)

            solution_matrix = np.reshape(solution.y,[p.num_groups,len(p.
↪compartment_names),p.max_days])

            # Get the effective R, betas, and g(t)
            G_t, I_t, new_infections, within_hosp_infections =␣
↪get_figure4_metrics(solution_matrix,p)

            # Get color for each model but don't use LSs
            ls, color = ls_styles[model]
            ls = '-'

            # Plot g(t)
            plt.sca(ax_G)
            plt.hlines([f1],xmin = t_lockdown, xmax = p.max_days,color = [0.5,0.
↪5,0.5,0.5],ls = '-',linewidth = linewidth,label='No difference')
            plt.plot(np.arange(0,max_days),G_t,ls = '-',color='k',linewidth =␣
↪linewidth)
            plt.xlabel("Days")
            plt.fill_between(y1=(0,0),y2=(1,1),x=(0,t_lockdown),color=[0.8,0.
↪8,0.8,0.5])
            plt.ylim([0,1.0])
            plt.xlim([0,xlim])
            if model_index == len(models) - 1:
                plt.legend(loc='upper left',
                           bbox_to_anchor = (0.36, 0.95),
                            **legend_keywords)

            # Plot I(t)
            I_styles = {0:('nEWs','-'),1:('EWs','--')}
            plt.sca(ax_I)

            for groupID in range(2):
                label,ls = I_styles[groupID]
                ax_I.plot(np.arange(0,max_days),I_t[groupID],color =␣
↪'k',linewidth = linewidth,ls=ls,label=label)

            ax_I.set_xlabel("Days")
            ax_I.set_xlim([0,xlim])
            ax_I.set_yscale('log')
```

```
            plt.fill_between(y1=(0,0),y2=(1,1),x=(0,t_lockdown),color=[0.8,0.
↪8,0.8,0.5])
            plt.ylim([1e-6,2e-1])
            if model_index == len(models) - 1:
                plt.legend(**legend_keywords,
                           loc='upper left',
                           bbox_to_anchor = (0.56, 0.95))

            # Plot where new infections come from
            plt.sca(ax_B)
            B_styles = {0:{0:([0/255,158/255,115/255,1],'-','NN'),
                           1:([0/255,158/255,115/255,1],'--','NE')},
                        1:{0:([204/255,121/255,167/255,1],'-','EN'),
                           1:([204/255,121/255,167/255,1],'--','EE')}}

            for infectious_groupID in [0,1]:
                for susceptible_groupID in [0,1]:
                    color,ls,label =␣
↪B_styles[infectious_groupID][susceptible_groupID]
                    plt.plot(np.
↪arange(0,max_days),new_infections[infectious_groupID,susceptible_groupID,:
↪],color = color,linewidth = linewidth,ls=ls,label=label)
                    if infectious_groupID == 0 and susceptible_groupID == 1:
                        plt.plot([],[],color=[0,0,0,0],label=' ')
            plt.plot(np.arange(0,max_days),within_hosp_infections,color = [230/
↪255,159/255,0/255,1],linewidth = linewidth,ls=':',label='WH')
            plt.xlabel("Days")
            plt.xlim([0,xlim])
            plt.ylim([1e-3,1.1])
            plt.yscale('log')
            plt.fill_between(y1=(0,0),y2=(2,2),x=(0,t_lockdown),color=[0.8,0.
↪8,0.8,0.5])
            if model_index == len(models) - 1:
                plt.legend(loc = 'upper left',
                           bbox_to_anchor = (0.36, 0.65),
                           **legend_keywords,ncol = 2)

            # Assign a column label
            assign_column_label_figure4(axes_I[model_index],p.model)
    assign_row_label_figure4(axes_I[0],'Prevalence','A',letter_pad = 30)
    assign_row_label_figure4(axes_G[0],'EW Fraction of Infected','B',letter_pad␣
↪= 30)
    assign_row_label_figure4(axes_B[0],'Proportion of\nNew␣
↪Infections','C',letter_pad = 30)
    for ax in axes_all.flat:
        ax.tick_params(width = 1, length = 3)
```

```
        ax.tick_params(width = 0.5, length = 1.5,which='minor')
    plt.subplots_adjust(wspace=0.25,hspace=0,right = 0.8)
    plt.tight_layout()
    fig.suptitle(figure_title,y = 1.02)
    plt.show()
```

The code below reproduces Figure 4 from the main text:

[46]:
```
update_font_size(10)
create_figure4()
```

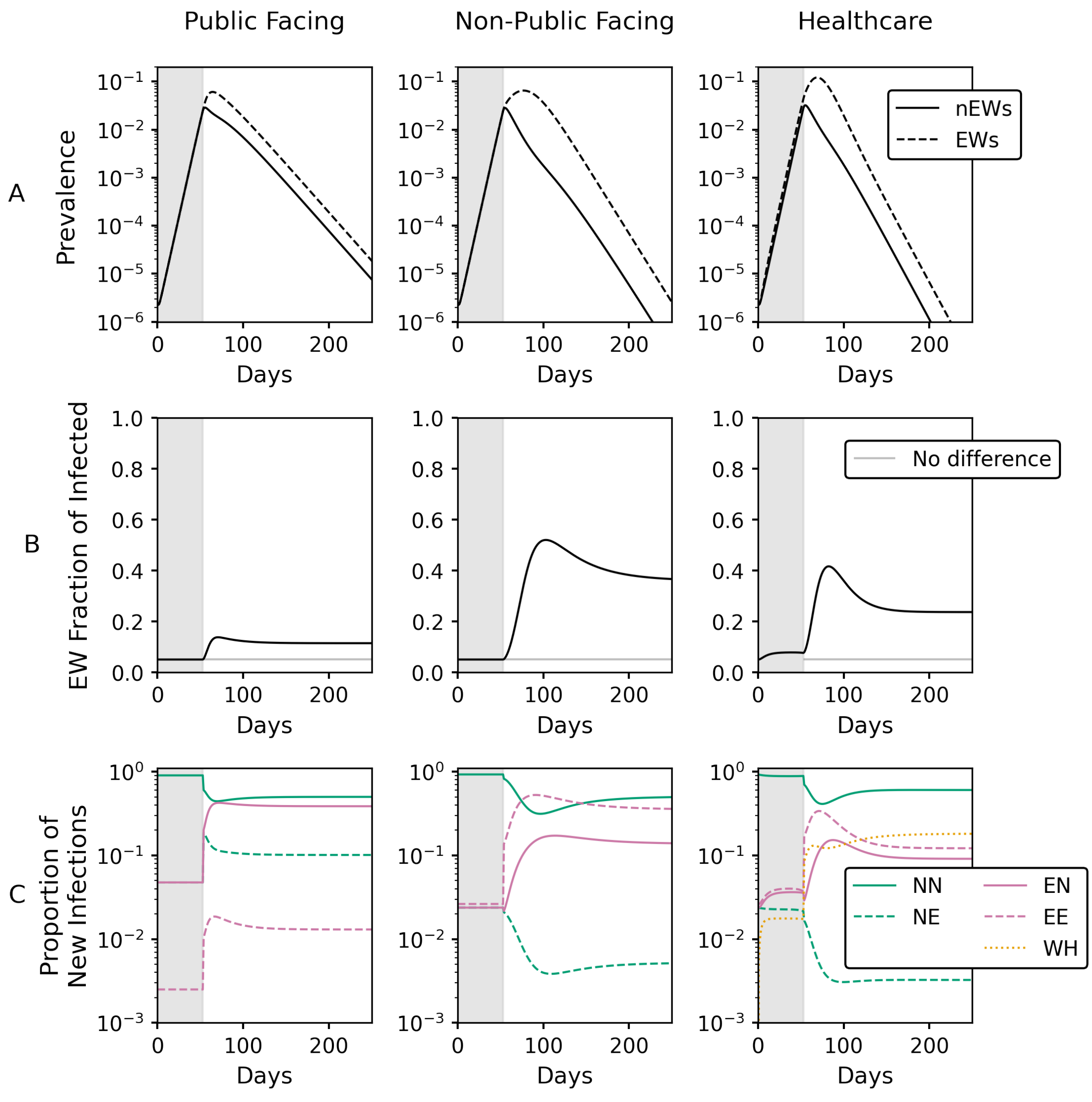

Figure 4. Time-resolved dynamics of infections

Alternative versions of Figure 4

[47]:
```
create_figure4(desired_Reff = 0.9)
```

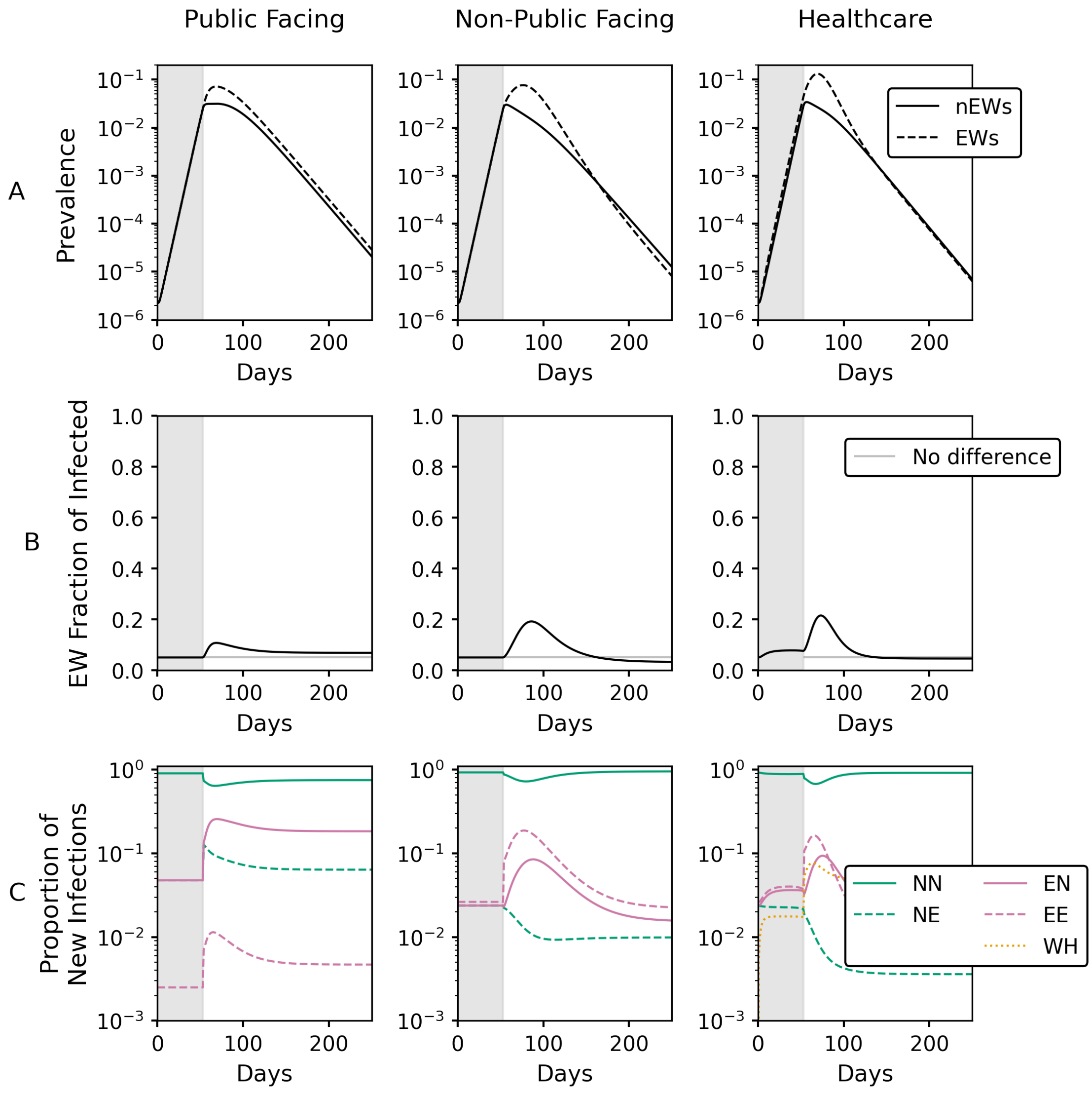

Figure S30. Time-resolved dynamics of infections when $R_0\theta = 0.9$. See Figure 4 for details.

[48]:
```
create_figure4(desired_Reff = 1.5)
```

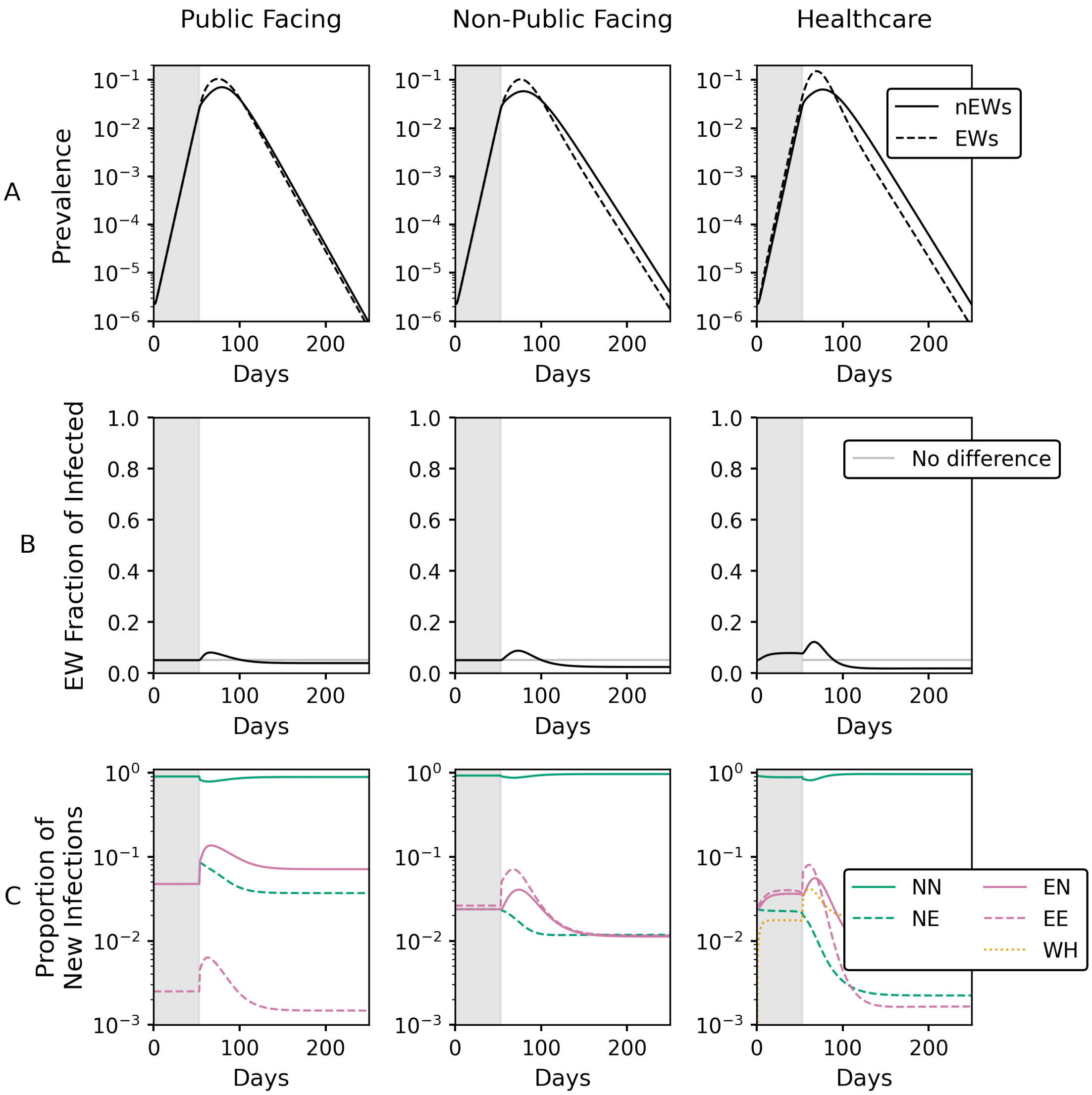

Figure S31. Time-resolved dynamics of infections when $R_0\theta = 1.5$. See Figure 4 for details.

[49]:
```
create_figure4(f = 0.1)
```

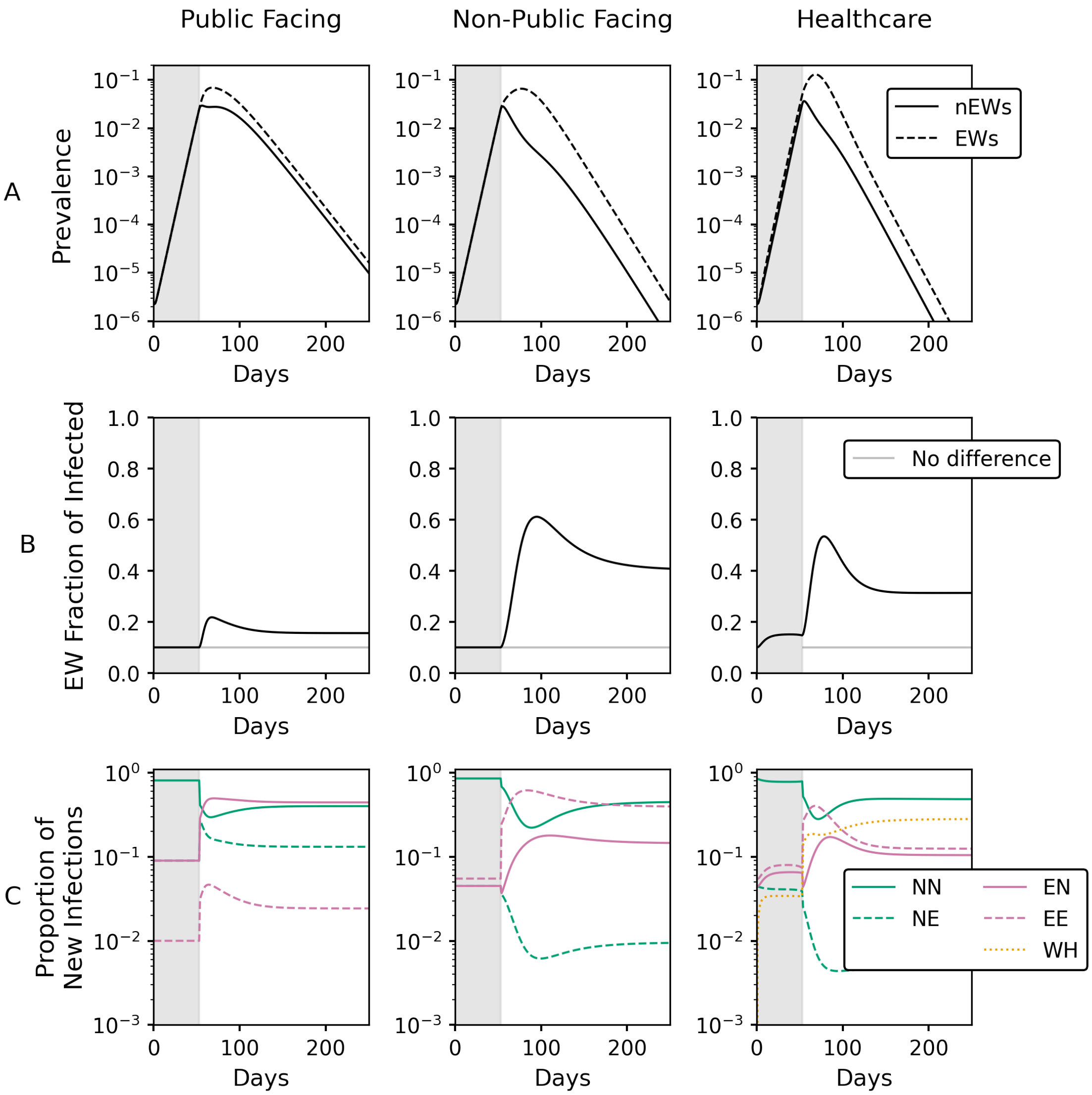

Figure S32. Time-resolved dynamics of infections when $f = 0.1$. See Figure 4 for details.

[50]:
```
create_figure4(t_lockdown = args_default.social_distancing_time - 10)
```

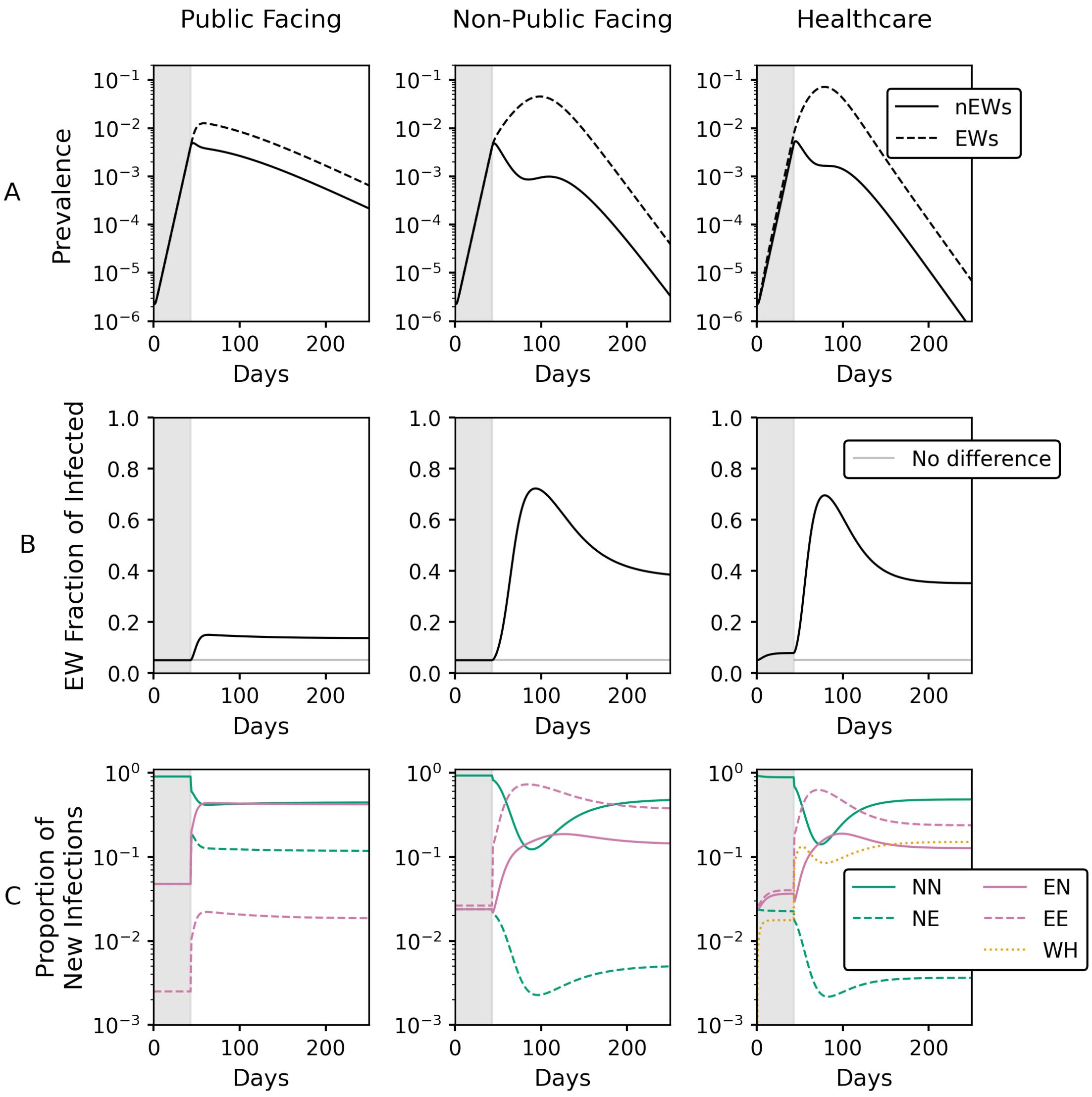

Figure S32. Time-resolved dynamics of infections when the time of lockdown is moved ten days earlier. See Figure 4 for details.

[51]:
```
create_figure4(I0=10)
```

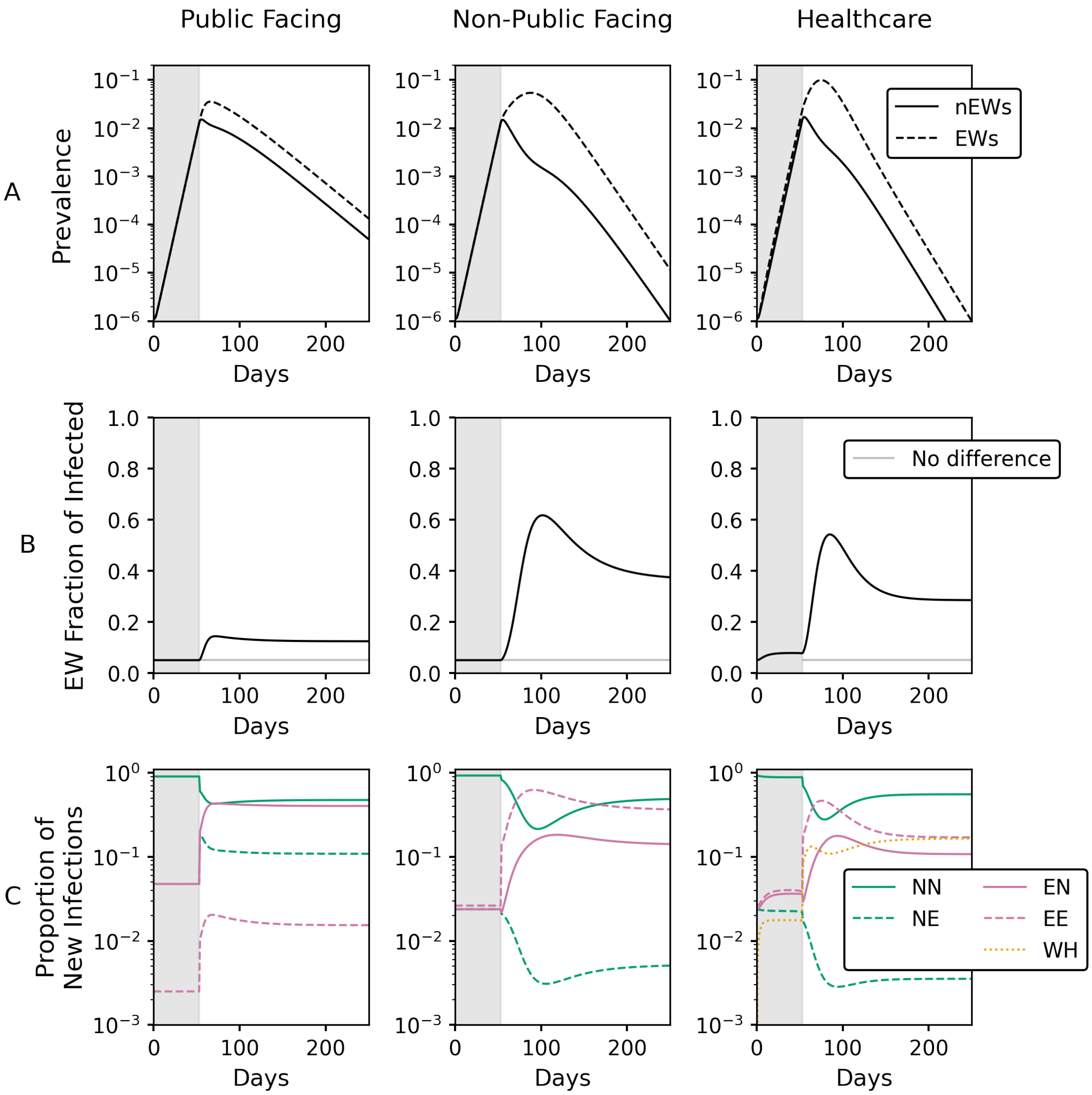

Figure S33. Time-resolved dynamics of infections when $I_0 = 10$. See Figure 4 for details.

[52]:
```
create_figure4(I0=40)
```

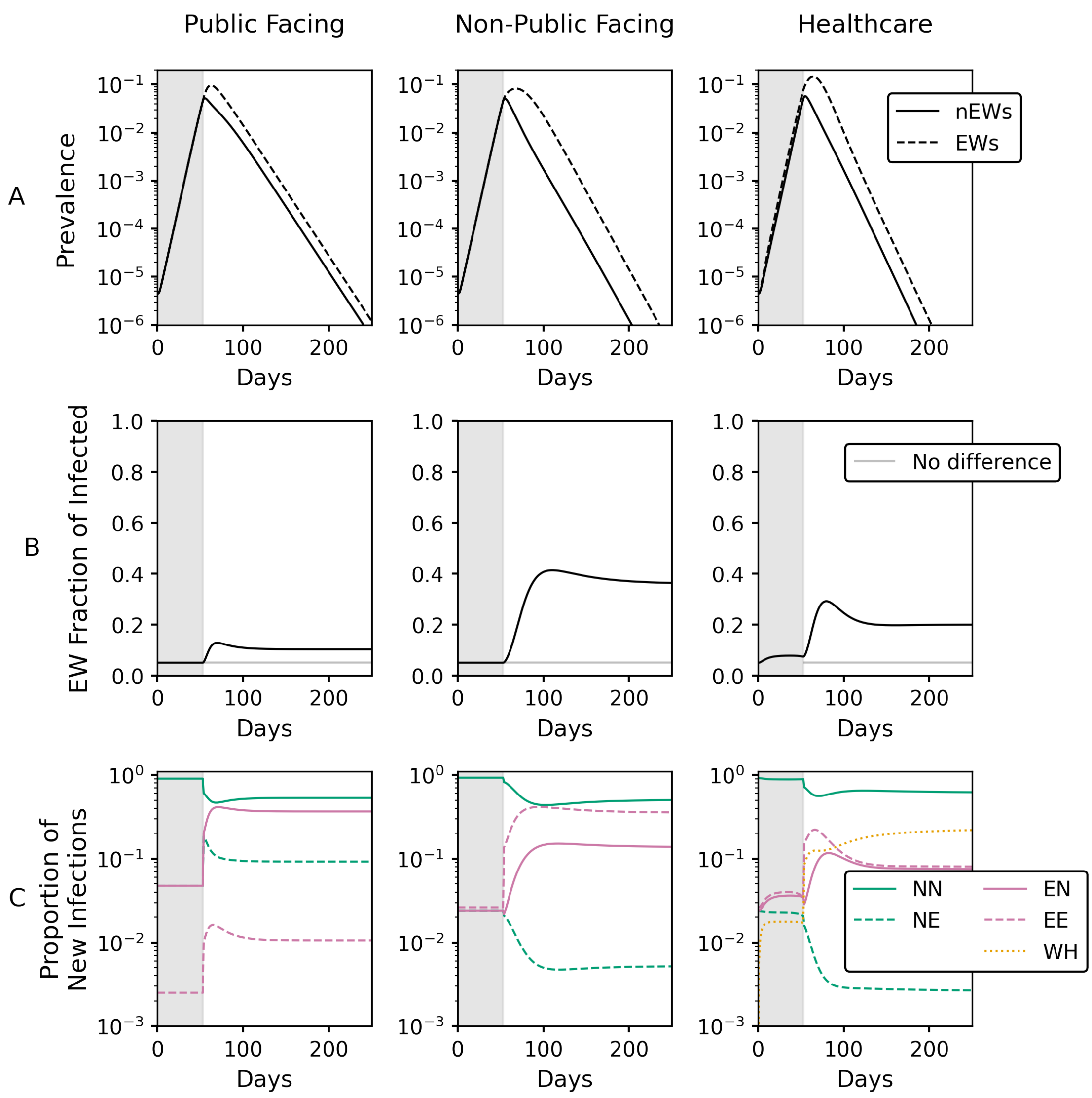

Figure S34. Time-resolved dynamics of infections when $I_0 = 40$. See Figure 4 for details.

[53]:
```
create_figure4(R_0=2.5)
```

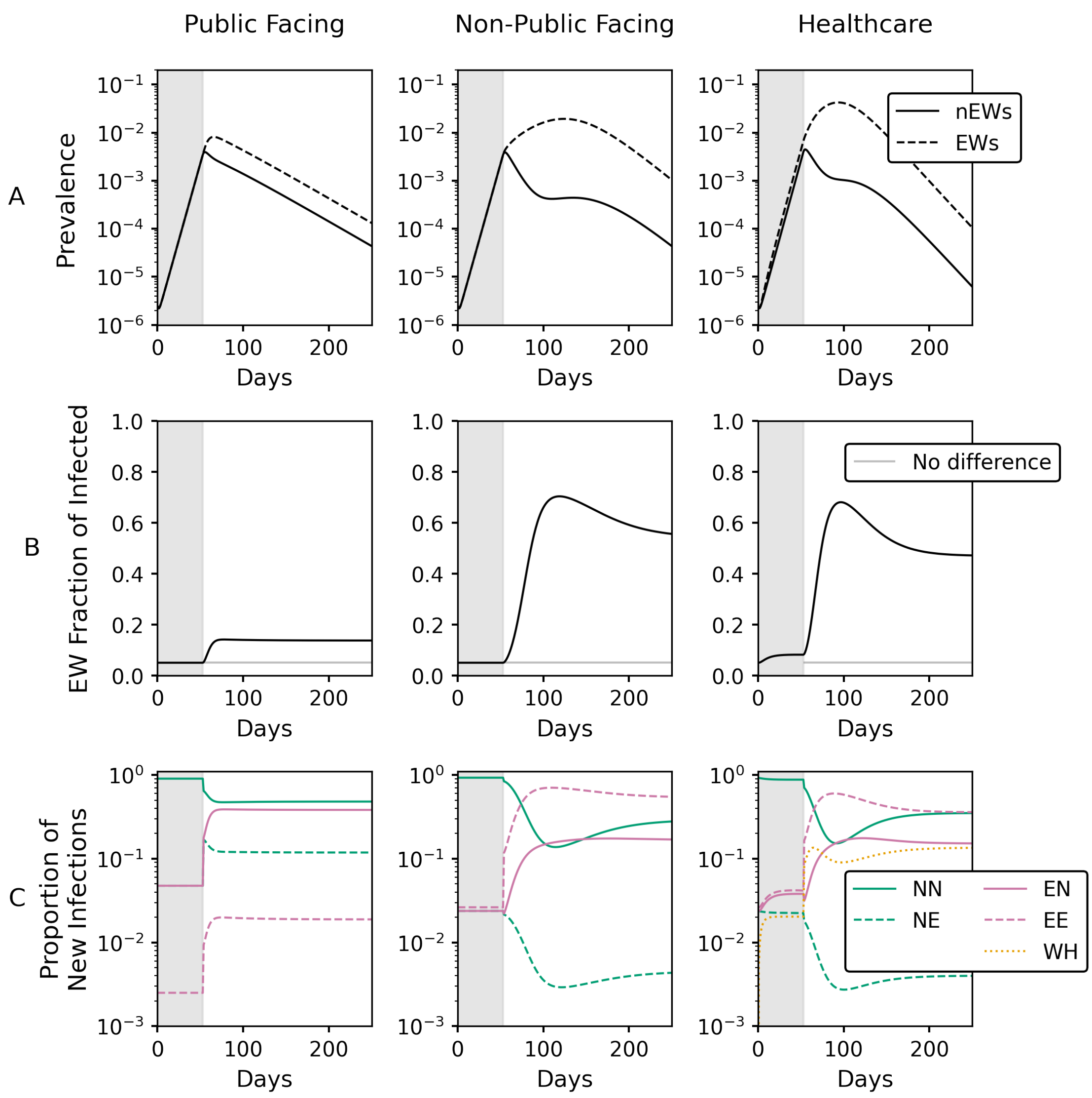

Figure S35. Time-resolved dynamics of infections when $R_0 = 2.5$. See Figure 4 for details.

[54]:
```
create_figure4(R_0=3.5)
```

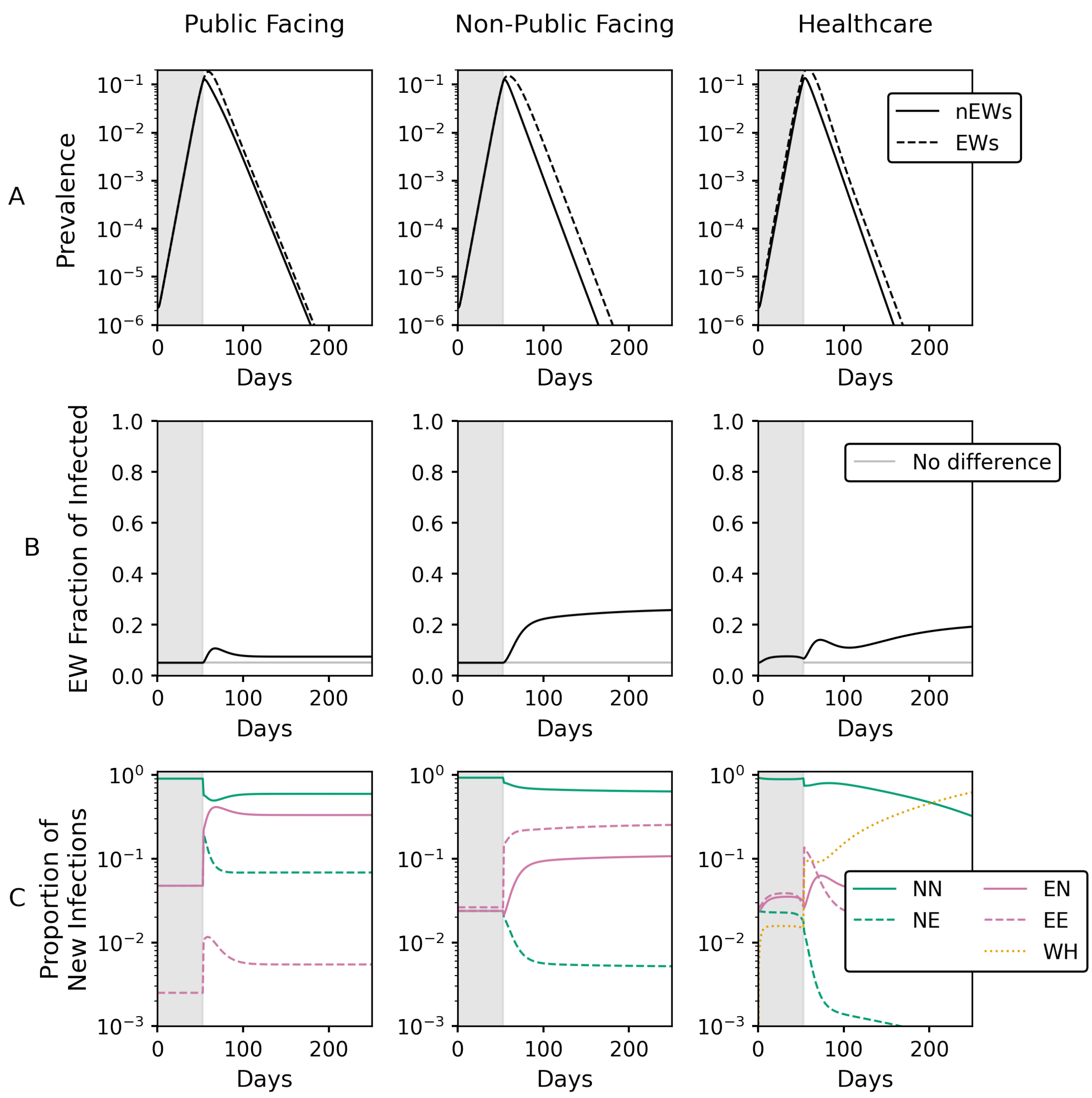

Figure S36. Time-resolved dynamics of infections when $R_0 = 3.5$. See Figure 4 for details.

[55]:

```
change_in_incubation_time = -1
set_parameters = {'t_inc':3+change_in_incubation_time}
create_figure4(set_parameters = set_parameters)
```

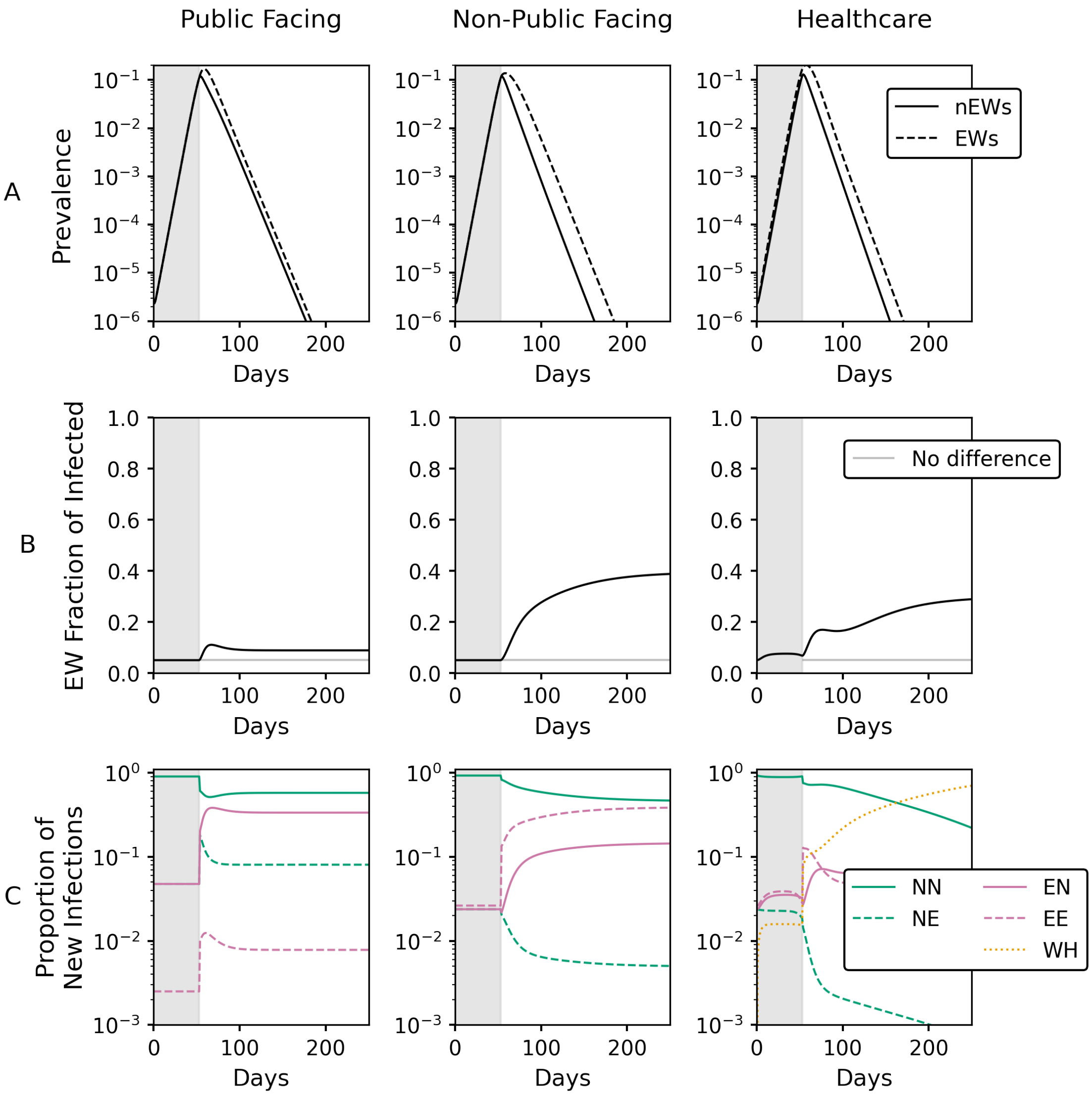

Figure S37. Time-resolved dynamics of infections when $t_E = 2$ days. See Figure 4 for details.

[56]:
```
change_in_incubation_time = 1
set_parameters = {'t_inc':3+change_in_incubation_time}
create_figure4(set_parameters = set_parameters)
```

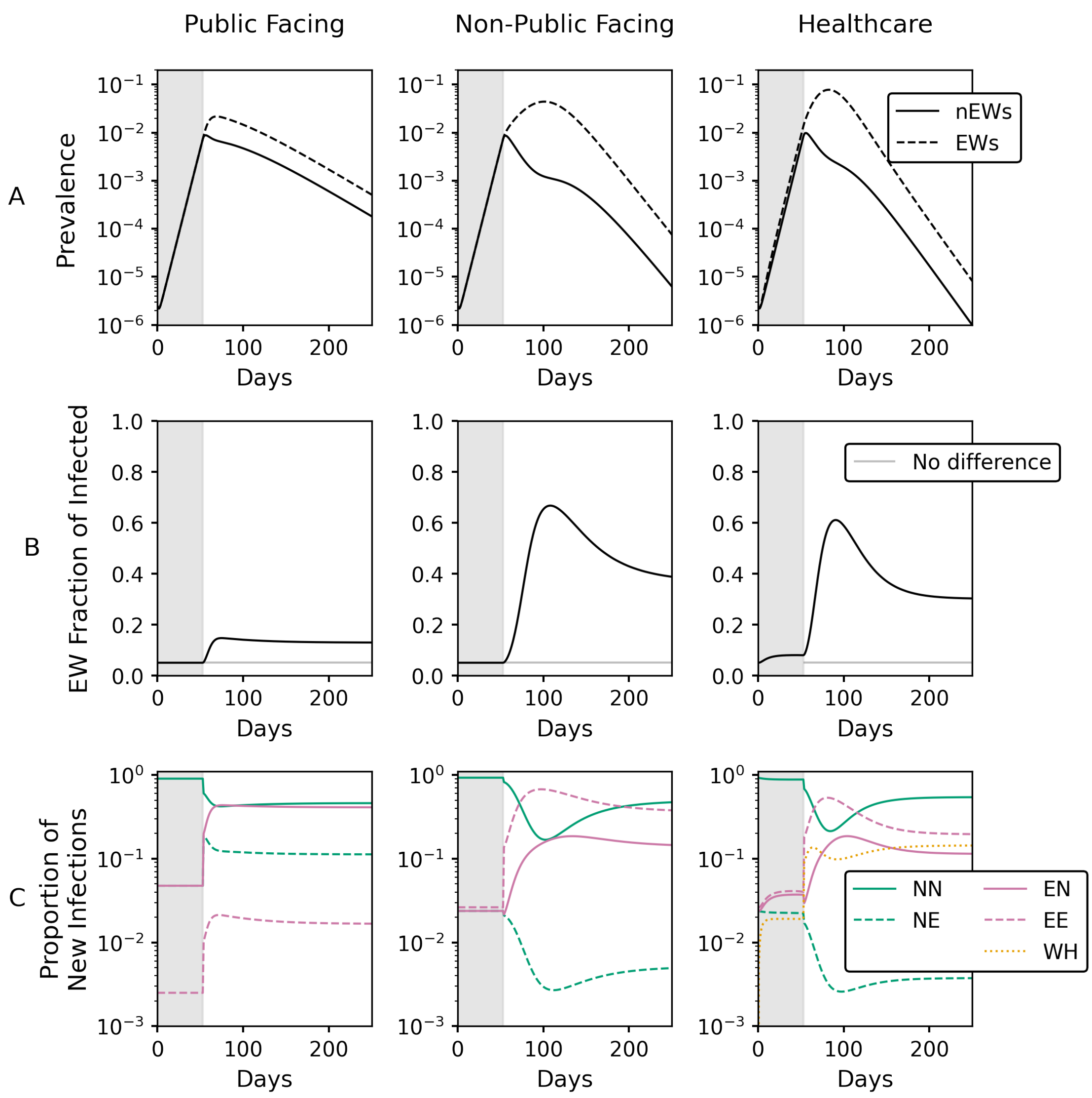

Figure S38. Time-resolved dynamics of infections when $t_E = 4$ days. See Figure 4 for details.

[57]:
```
increase_time_infectious = 5
set_parameters = {'t_AR':5 + increase_time_infectious,
                  't_IR':5 + increase_time_infectious,
                  't_IH':5 + increase_time_infectious}
create_figure4(set_parameters = set_parameters)
```

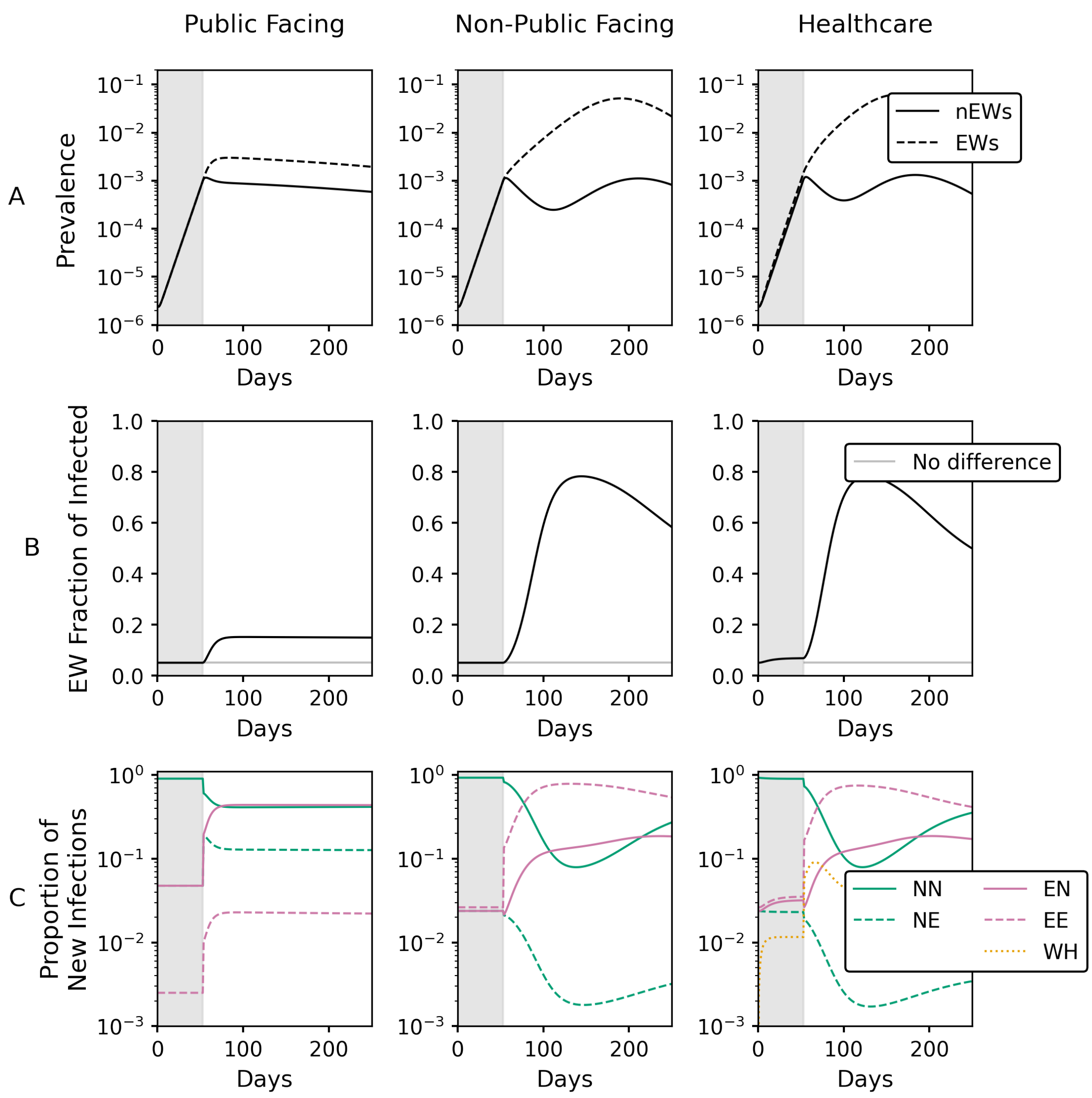

Figure S39. Time-resolved dynamics of infections when the infectious period is increased by five days (i.e., $t_{IA} = t_{IR} = t_{IH} = 10$ days). See Figure 4 for details.

[58]:
```
create_figure4(model_dependent_rho = {'No Structure': 0,
                      'Cashier': 0,
                      'Healthcare': 0,
                      'USPS': 0,
                      'Healthcare_Sigmoid':  0})
```

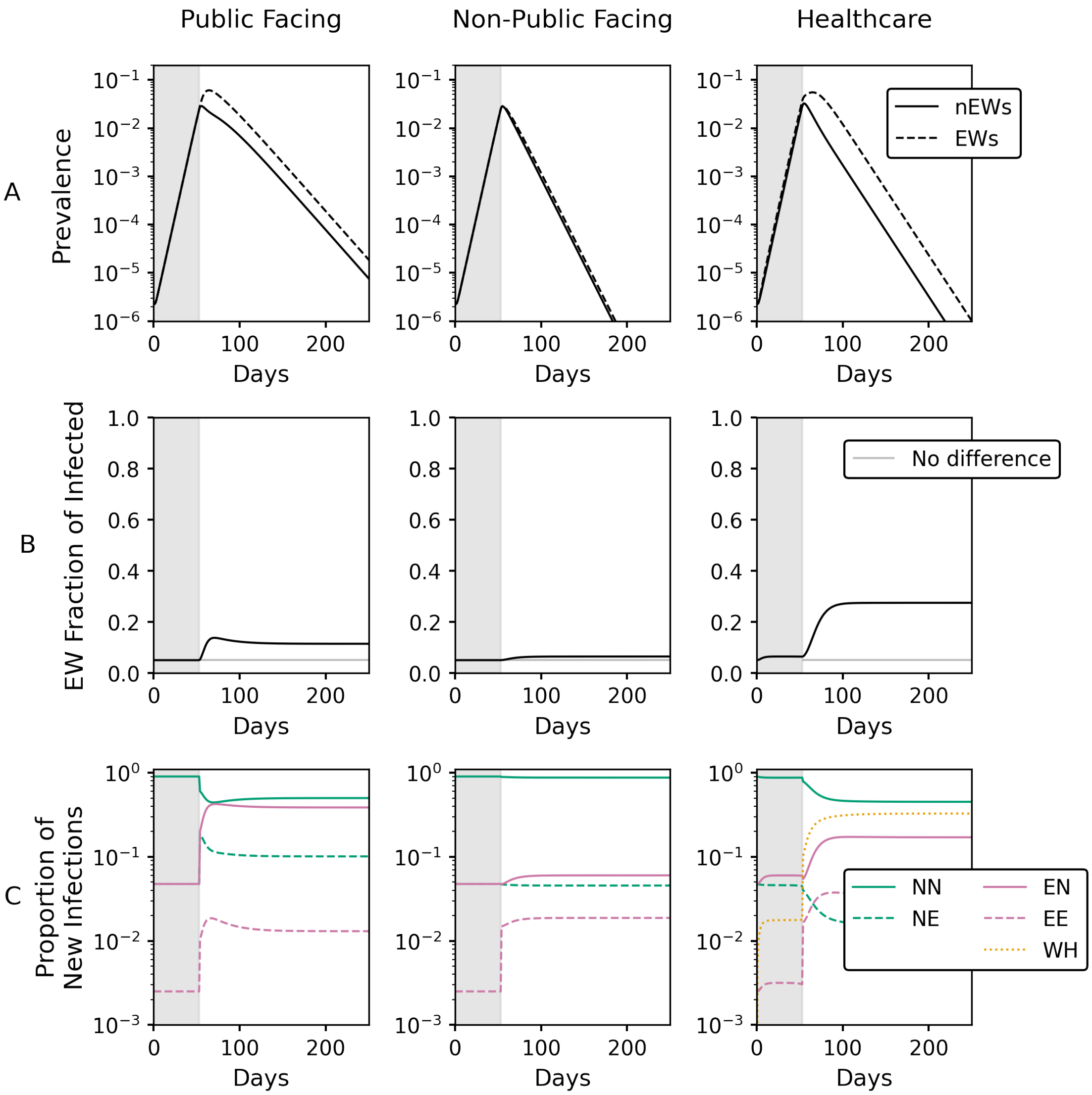

Figure S40. Time-resolved dynamics of infections when all contacts are proportionately distributed (i.e., $\rho = 0$). Reducing ρ changes the non-public-facing and healthcare EW models. The public-facing EW model shown here is unchanged from that in the main. When $\rho = 0$, the number of contacts unaffected by SIP under the non-public-facing EW model is too low to cause substantial differences compared to a model without EWs. See Figure 4 for details.

[59]:
```
create_figure4(model_dependent_rho = {'No Structure': 0.5,
                                      'Cashier': 0.5,
                                      'Healthcare': 0.5,
                                      'USPS': 0.5,
                                      'Healthcare_Sigmoid':  0.5})
```

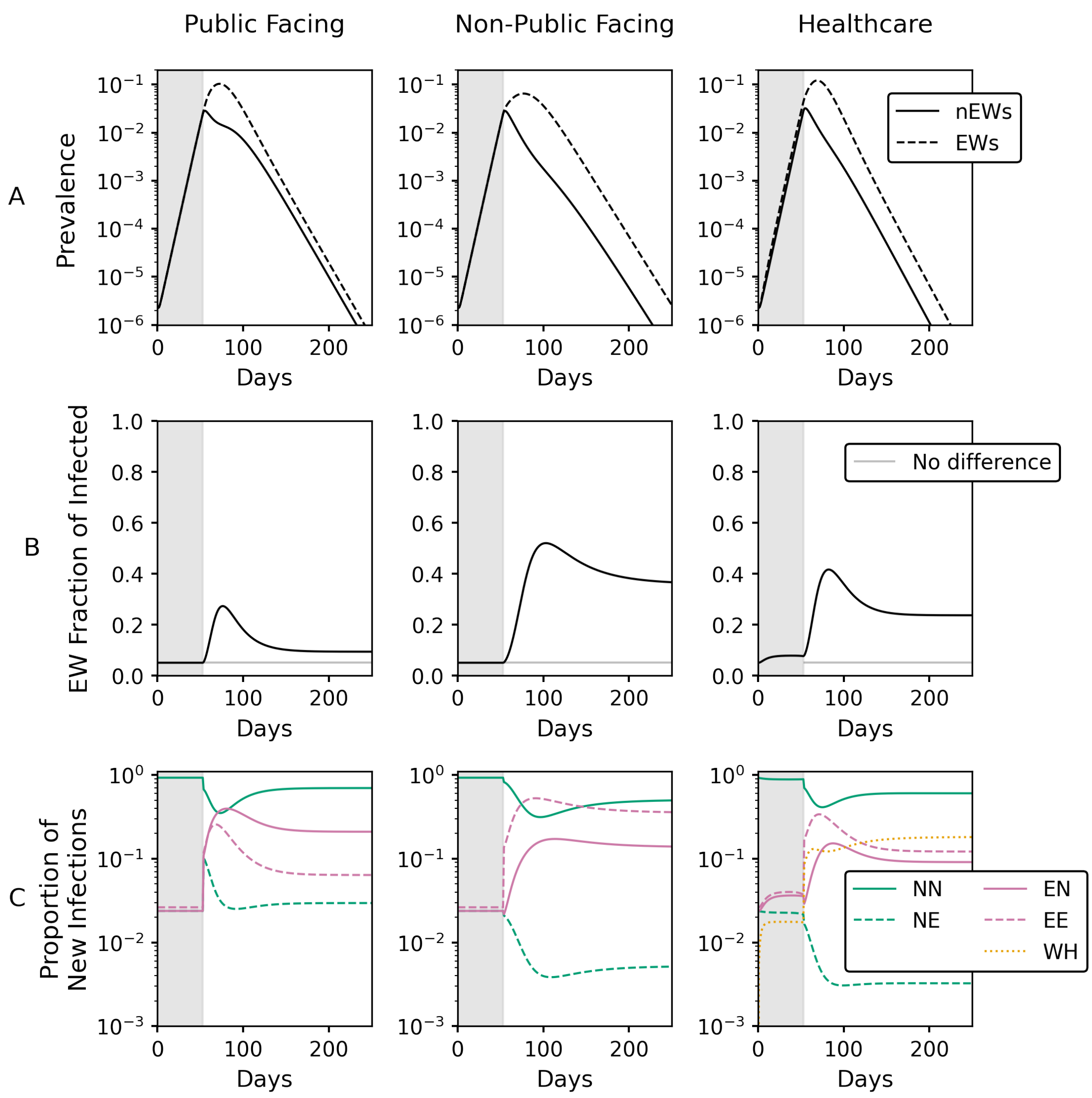

Figure S41. Time-resolved dynamics of infections when all contacts are proportionately distributed (i.e., $\rho = 0.5$). Increasing ρ changes the public-facing EW model. The non-public-facing EW and healthcare EW models shown here are unchanged from Figure 4. See Figure 4 for details.

[60]:
```
change_in_percent_hospitalized = 0.5
set_parameters = {'p_IH':0.066*change_in_percent_hospitalized}
create_figure4(set_parameters = set_parameters)
```

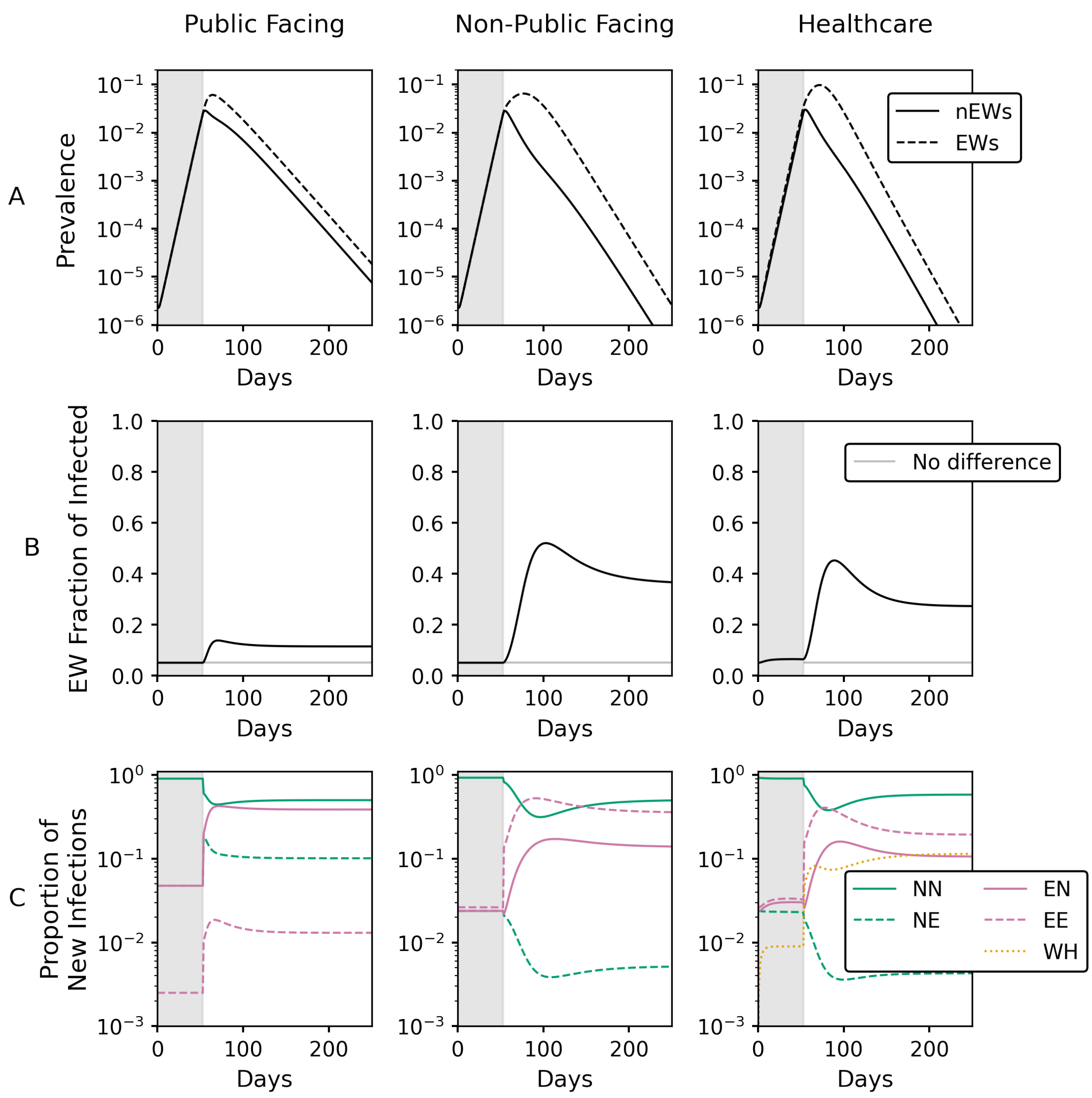

Figure S42. Time-resolved dynamics of infections when the proportion of infections that require hospitalization is halved. This parameter only affects the healthcare EW model. See Figure 4 for more details.

[61]:
```
change_in_percent_hospitalized = 2
set_parameters = {'p_IH':0.066*change_in_percent_hospitalized}
create_figure4(set_parameters = set_parameters)
```

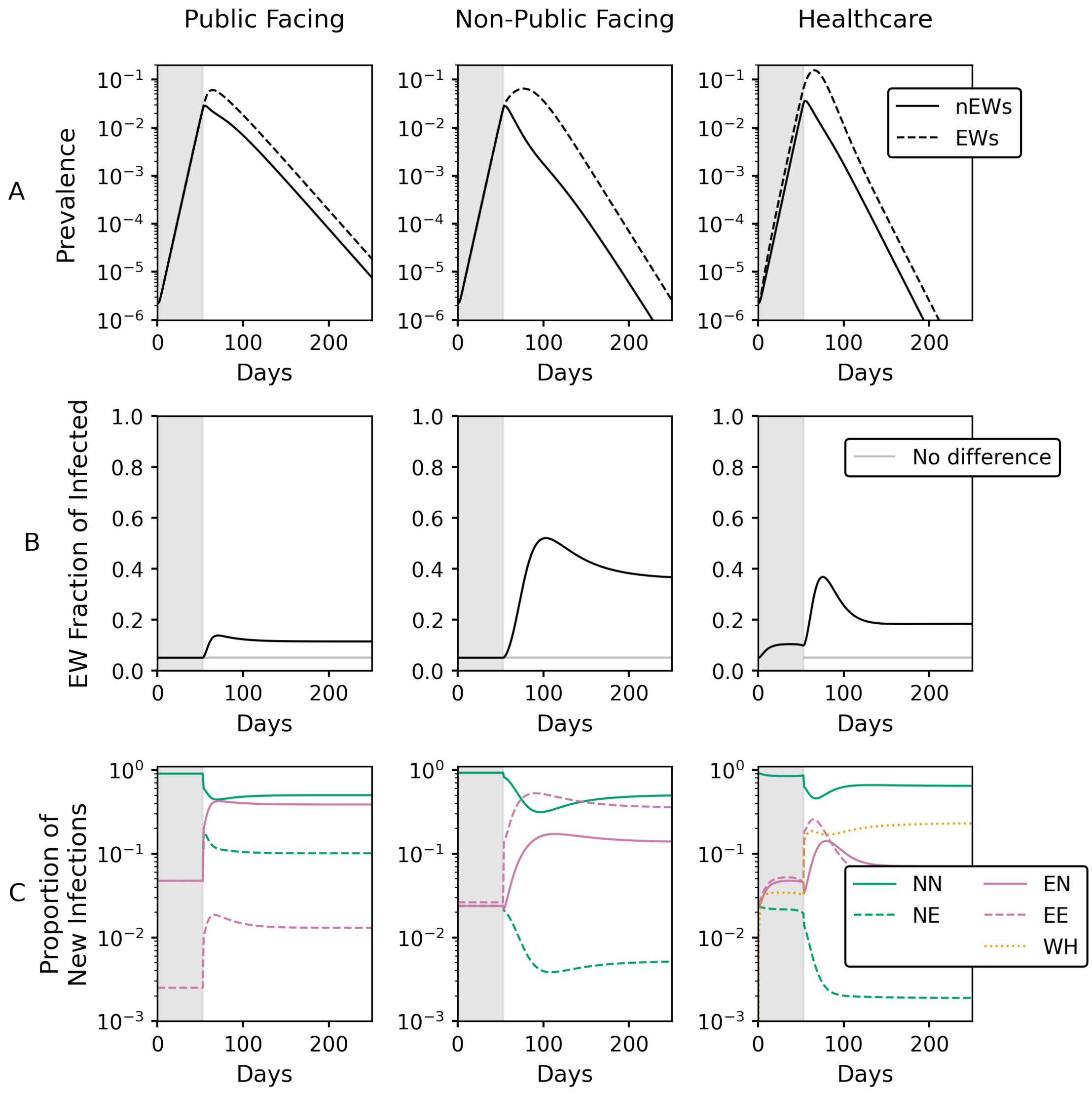

Figure S43. Time-resolved dynamics of infections when the proportion of infections that require hospitalization is doubled. This parameter only affects the healthcare EW model. See Figure 4 for more details.

[62]:
```
change_time_hospitalized = 0.5
set_parameters = {'t_HR':8*change_time_hospitalized,
                  't_HC':6*change_time_hospitalized}
create_figure4(set_parameters = set_parameters)
```

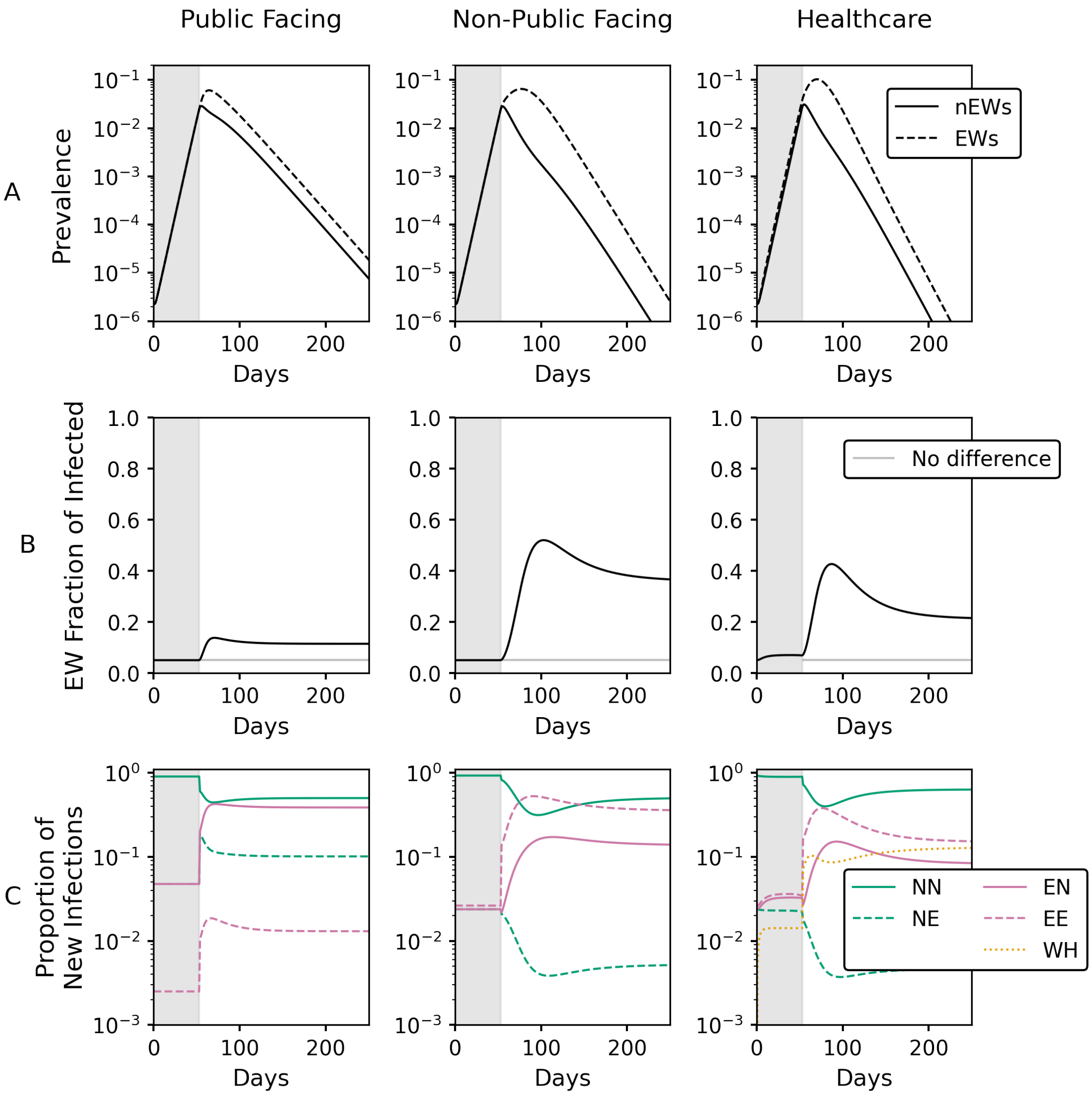

Figure S44. Time-resolved dynamics of infections when the time spent in the initial hospital compartment is halved. This parameter only affects the healthcare EW model. See Figure 4 for more details.

[63]:
```
change_time_hospitalized = 2
set_parameters = {'t_HR':8*change_time_hospitalized,
                  't_HC':6*change_time_hospitalized}
create_figure4(set_parameters = set_parameters)
```

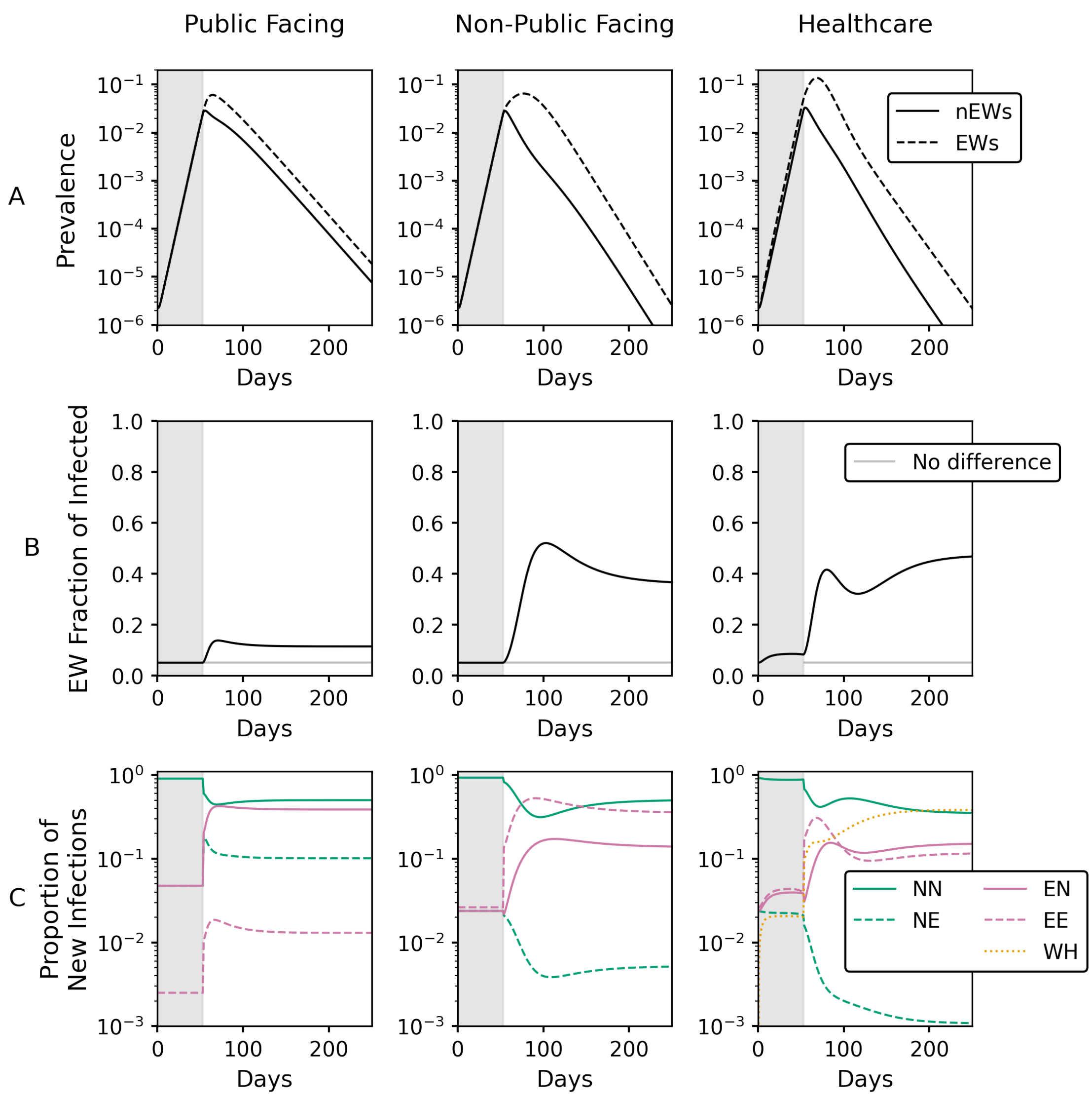

Figure S45. Time-resolved dynamics of infections when the time spent in the initial hospital compartment is doubled. This parameter only affects the healthcare EW model. See Figure 4 for more details.

[64]:
```
include_critical_care = False
create_figure4()
include_critical_care = True
```

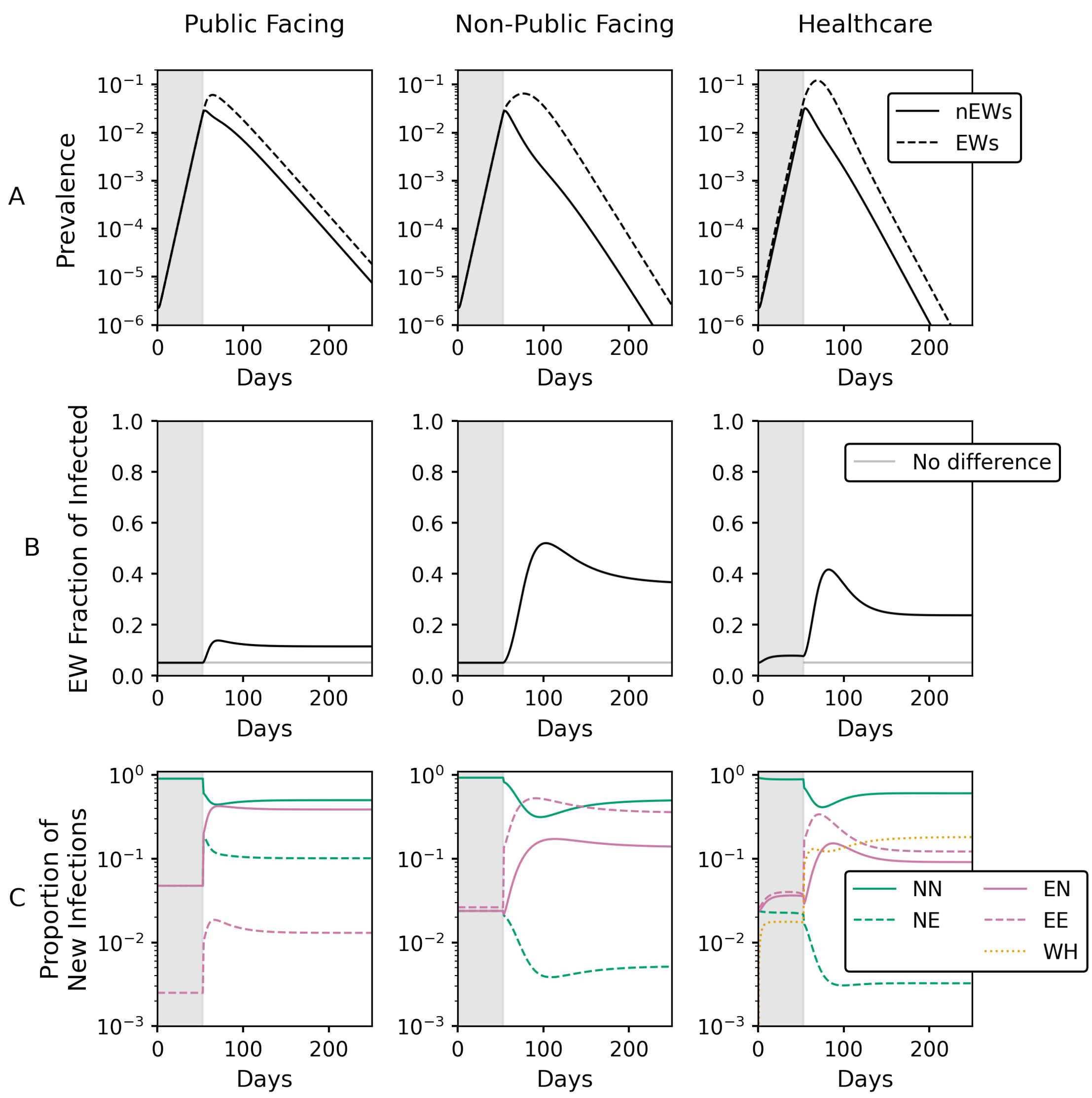

Figure S46. Time-resolved dynamics of infections when patients in critical care are not considered infectious. This parameter only affects the healthcare EW model. See Figure 4 for more details.

0.1.5 S5 - Fitting to empirical data

Although the goal of this work is not to model the outbreak in a particular city/region or use it to make quantitative predictions, we can assess how well the parameter choices and the overall model match the progression of the ongoing pandemic by fitting to empirical data. Following others, we focus on the number of deaths rather than of cases, because (although not without problems) these numbers are reliable. Here, we use publically available cumulative deaths for three U.S. cities: Seattle, Chicago, and New York City. To focus on a more reliable subset of the data, we remove the most recent 2 days and all dates before the 10th reported death.

For New York City, we note that while both confirmed and probable deaths due to Covid-19 are

reported, probable deaths were not added until March 11th, and over 4500 were added retroactively in mid-April. Even these are likely an underestimate as more than 4200 deaths remain unexplained.

In the code block below, we read in and format publically available data on Covid-19 deaths (and hospitalizations for NYC).

[65]:
```
# The fits are using data that is current on 5/3/2020
# Read in Seattle data
# Source for all US counties: https://raw.githubusercontent.com/nytimes/
↪covid-19-data/master/us-counties.csv
counties_cases = pd.read_csv("./us-counties.csv")
counties_cases.rename(columns={'date':'DATE_OF_INTEREST'}, inplace=True)
counties_cases['DATE_OF_INTEREST'] = pd.
↪to_datetime(counties_cases['DATE_OF_INTEREST'])
seattle_cases = counties_cases[counties_cases['county']=='King']
seattle_cases = seattle_cases[seattle_cases['state']=='Washington']
seattle_cases.reset_index(drop=True, inplace=True)
nrows = seattle_cases.shape[0]
seattle_cases = seattle_cases.drop([nrows-1,nrows-2])
seattle_cases["cumulative_cases"] = seattle_cases["cases"]
seattle_cases["cumulative_deaths"] = seattle_cases["deaths"]
first_date = seattle_cases['DATE_OF_INTEREST'][0]
seattle_cases["day_num"] = (seattle_cases['DATE_OF_INTEREST'] - first_date).dt.
↪days.astype(int)
day_of_tenth_death = seattle_cases[seattle_cases["cumulative_deaths"]>10].
↪DATE_OF_INTEREST.iloc[0]
seattle_cases =␣
↪seattle_cases[seattle_cases["DATE_OF_INTEREST"]>=(day_of_tenth_death)]
seattle_cases.reset_index(drop=True, inplace=True)
#print(seattle_cases.head())

# Create Chicago data
chicago_cases = counties_cases[counties_cases['county']=='Cook']
chicago_cases = chicago_cases[chicago_cases['state']=='Illinois']
chicago_cases.reset_index(drop=True, inplace=True)
nrows = chicago_cases.shape[0]
chicago_cases = chicago_cases.drop([nrows-1,nrows-2])
chicago_cases["cumulative_cases"] = chicago_cases["cases"]
chicago_cases["cumulative_deaths"] = chicago_cases["deaths"]
first_date = chicago_cases['DATE_OF_INTEREST'][0]
chicago_cases["day_num"] = (chicago_cases['DATE_OF_INTEREST'] - first_date).dt.
↪days.astype(int)
day_of_tenth_death = chicago_cases[chicago_cases["cumulative_deaths"]>10].
↪DATE_OF_INTEREST.iloc[0]
chicago_cases =␣
↪chicago_cases[chicago_cases["DATE_OF_INTEREST"]>=(day_of_tenth_death)]
chicago_cases.reset_index(drop=True, inplace=True)
```

```
#print(chicago_cases.head())

# Read in NYC data
# Cases, hospitalization data: https://raw.githubusercontent.com/nychealth/
↪coronavirus-data/master/case-hosp-death.csv
# Probable deaths: https://raw.githubusercontent.com/nychealth/coronavirus-data/
↪master/probable-confirmed-dod.csv
# Read in NYC data
nyc_cases = pd.read_csv("./case-hosp-death.csv")
nrows = nyc_cases.shape[0]
nyc_cases = nyc_cases.drop([nrows-1,nrows-2])
# Replace NA with 0
nyc_cases = nyc_cases.fillna(0)
nyc_cases['DATE_OF_INTEREST'] = pd.to_datetime(nyc_cases['DATE_OF_INTEREST'])
first_date = nyc_cases['DATE_OF_INTEREST'][0]
nyc_cases["day_num"] = (nyc_cases['DATE_OF_INTEREST'] - first_date).dt.days.
↪astype(int)
nyc_cases["cumulative_cases"] = nyc_cases["CASE_COUNT"].sort_index().cumsum()
nyc_cases["cumulative_hospitalized"] = nyc_cases["HOSPITALIZED_COUNT"].
↪sort_index().cumsum()

nyc_prob_deaths = pd.read_csv("./probable-confirmed-dod.csv") #Read in probable␣
↪deaths
nyc_prob_deaths = nyc_prob_deaths.fillna(0)
nyc_prob_deaths['DATE_OF_INTEREST'] = pd.
↪to_datetime(nyc_prob_deaths['date_of_death'])
nyc_cases = nyc_cases.merge(nyc_prob_deaths, on='DATE_OF_INTEREST')
nyc_cases["all_deaths"] = nyc_cases["CONFIRMED_COUNT"] +␣
↪nyc_cases["PROBABLE_COUNT"]
nyc_cases["cumulative_deaths"] = nyc_cases["all_deaths"].sort_index().cumsum()
day_of_tenth_death = nyc_cases[nyc_cases["cumulative_deaths"]>10].
↪DATE_OF_INTEREST.iloc[0]
nyc_cases = nyc_cases[nyc_cases["DATE_OF_INTEREST"]>=(day_of_tenth_death)]
#print(nyc_cases.head())

# Make plots showing real data in Seattle
update_font_size(10,rcParams = mpl.rcParams)
plt.plot(seattle_cases.day_num, seattle_cases.cumulative_cases, color='blue',␣
↪linewidth=1, label='Cumulative Cases')
plt.plot(seattle_cases.day_num, seattle_cases.cumulative_deaths, color='red',␣
↪linewidth=1, label='Cumulative Deaths')
plt.xlabel('Days since first case in Seattle')
plt.title('COVID-19 cases and deaths in Seattle')
plt.ylabel('Number of Individuals')
plt.legend(loc='best')
plt.yscale('log')
```

```
plt.show()

# Make plots showing real data in Chicago
plt.plot(chicago_cases.day_num, chicago_cases.cumulative_cases, color='blue',␣
 ↪linewidth=1, label='Cumulative Cases')
plt.plot(chicago_cases.day_num, chicago_cases.cumulative_deaths, color='red',␣
 ↪linewidth=1, label='Cumulative Deaths')
plt.xlabel('Days since first case in Chicago')
plt.title('COVID-19 cases and deaths in Chicago')
plt.ylabel('Number of Individuals')
plt.legend(loc='best')
plt.yscale('log')
plt.show()



# Reset the indexes for all locations
nyc_cases.reset_index(drop=True, inplace=True)
seattle_cases.reset_index(drop=True, inplace=True)
chicago_cases.reset_index(drop=True, inplace=True)
```

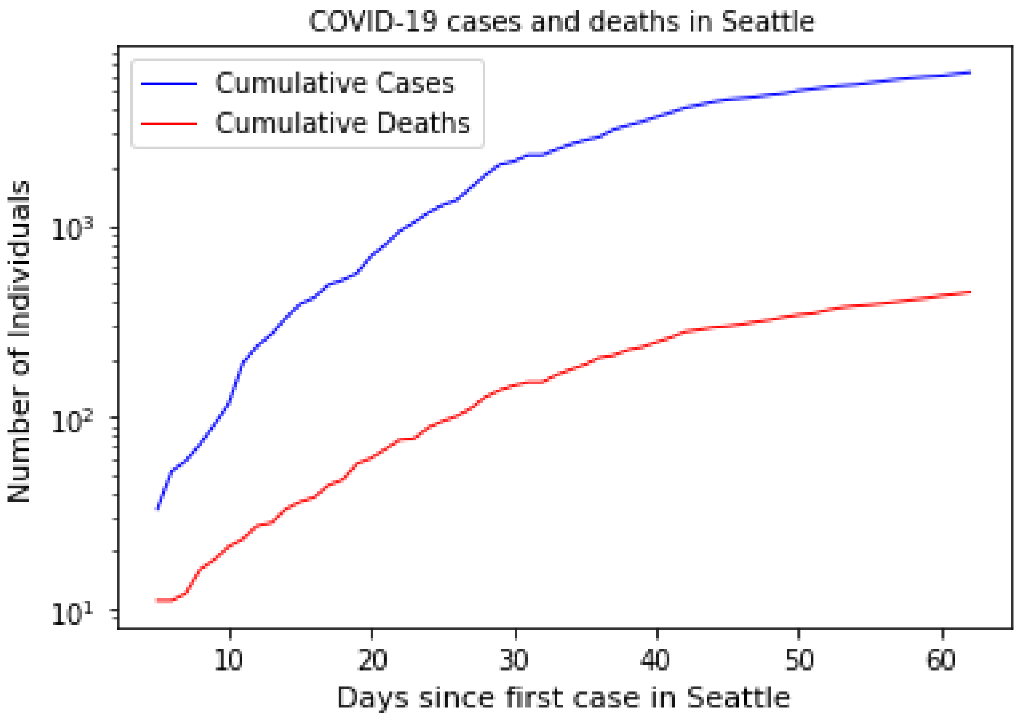

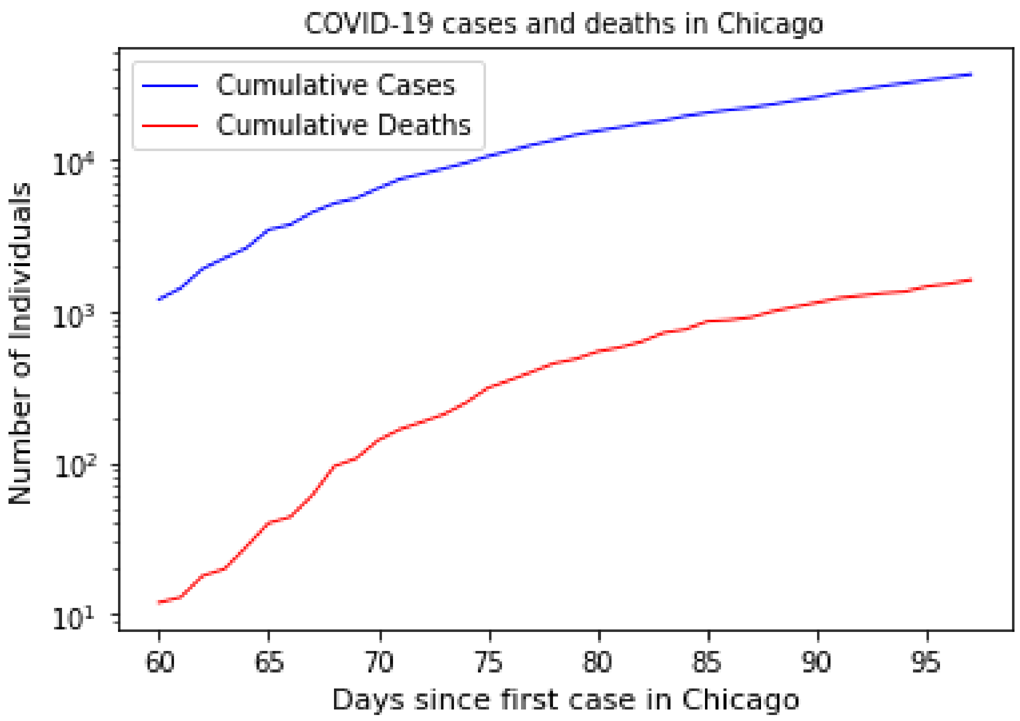

Figure S47. COVID-19 in Chicago and Seattle. The "steps" in the Chicago data correspond to a weekend effect, where reported deaths are decreased for Saturdays and Sundays.

[66]:
```
# Make plots showing real data in New York City
plt.plot(nyc_cases.day_num, nyc_cases.cumulative_cases, color='blue', ls='--',␣
↪linewidth=1, label='Cumulative Cases')
plt.plot(nyc_cases.day_num, nyc_cases.cumulative_hospitalized, color='purple',␣
↪ls=':', linewidth=1, label='Cumulative Hospitalizations')
plt.plot(nyc_cases.day_num, nyc_cases.cumulative_deaths, color='red',␣
↪linewidth=1, label='Cumulative Deaths')
plt.xlabel('Days since first case in NYC')
plt.ylabel('Number of Individuals')
plt.legend(loc='best')
plt.yscale('log')
plt.title(f'Figure S{figure_number()}. COVID Data in New York City (NYC) at␣
↪beginning of pandemic')

plt.show()
```

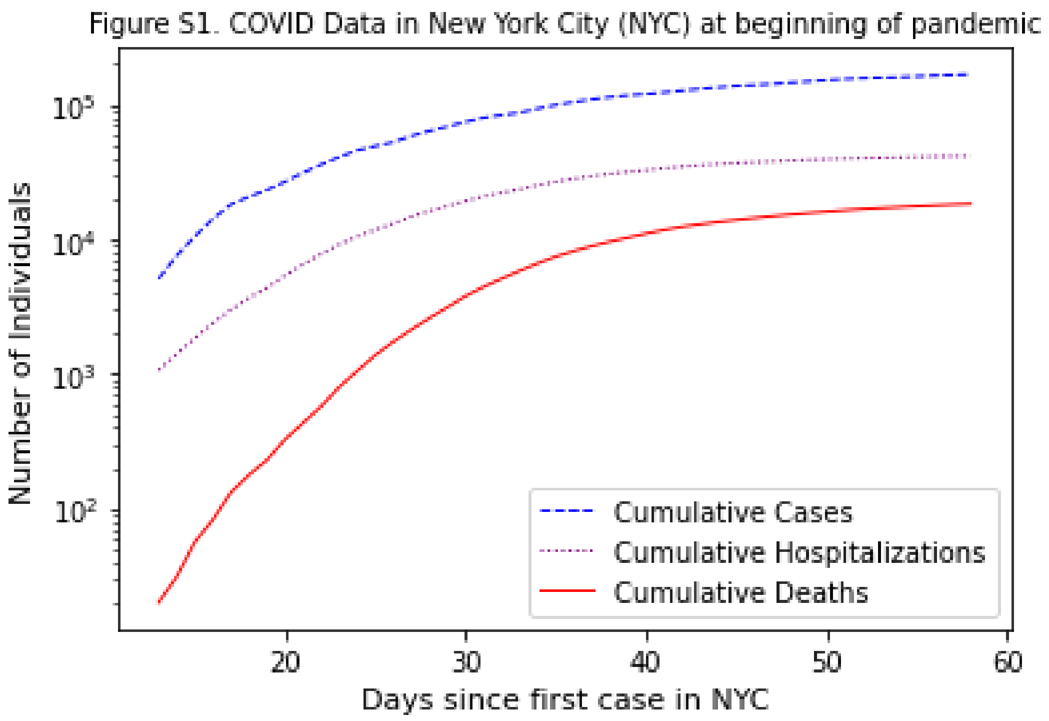

Figure S48. COVID-19 cases, hospitalizations, and deaths in NYC at beginning of pandemic.

The function below is used to compute the error of the model during the optimization procedure. Here, we are minimizing the root-mean-square-error (RMSE) of the per-day change in the death count. In the optimization presented below, we are inferring θ, the starting time of the infection, and the time of lockdown relative to the first case.

[67]:
```
def SEIR_model_loss(params_to_estimate, data, n_forecast, model, lockdown_t,␣
↪start_t, N=8e6, fit_hospital=False, adjust_beta_hosp=1, rho=1,   error=True):

    # Parameters passed from optimization algorithm
    R_0, theta, lockdown_t = params_to_estimate[0:3]
    pred_infected = 20

    if theta < 0 or R_0 < 0 or lockdown_t < 0 or start_t < 0:
        return np.inf

    # Get actual data
    n_infected = data['cumulative_cases']
    n_deaths = data['cumulative_deaths']

    # Number of days that we have data for
    n_train = data.shape[0]
```

```
    # Define how many days to simulate
    max_days = n_forecast

    # Define proportion essential
    prop_essential = 0.1

    # Number of groups
    num_groups = 2

    # Define the compartments
    compartment_names = ["S","E","IA","IR","IH","HR","HC","CR","CD","L","R","D"]
    compartment_formulas =␣
↪[dS_dt,dE_dt,dIA_dt,dIR_dt,dIH_dt,dHR_dt,dHC_dt,dCR_dt,dCD_dt,dL_dt,dR_dt,dD_dt]
    compartments = namedtuple("compartments",compartment_names)

    # Define the initial conditions & population size
    n_exposed  = 0
    N = N

    # Social interaction effects: C
    # C[i][j] = proportion of group I's contacts that are with group
    # rho allows deviations from proportionate mixing
    C = np.array([    (1 - prop_essential) + rho * prop_essential,
                      (1 - rho) * (1 - prop_essential),
                      (1 - rho) * (prop_essential),
                      1 - (1 - rho) * (1 - prop_essential) - rho])
    C = np.reshape(C,[num_groups,num_groups])

    # Convert initial state into 1-dimensional array

    t_span = [0, max_days]
    t_eval = np.arange(max_days)
    social_distancing_time = lockdown_t
    args = parameters(R_0 = R_0,                                 # R_0: used to␣
↪calculate betas
                      N = N,                                     # total population␣
↪size
                      C = C,                                     # Mixing matrix

                      social_distancing_time = social_distancing_time,    # when␣
↪does social distancing start
                      theta = theta,                             # How effective is␣
↪social distancing
                      model = model,                             # Who social␣
↪distances?
                      proportion_essential = prop_essential, #
```

```
                    rho = rho,                         # record changes from␣
↪proportionate mixing

                    max_days = max_days,                   # bookkeeping of␣
↪how many days simulated
                    num_groups = num_groups,               # bookkeeping of␣
↪how many groups
                    compartment_names = compartment_names, # bookkeeping of␣
↪how many compartments

                    t_inc =  3,    # !time from E to I: exposed to infected
                    t_AR =   5,    # !time from IA to R: infected and␣
↪asymptomatic to recovered
                    t_IR =  5,    # !time from IR to R: infected, symptomatic␣
↪and will recover to recovered
                    t_IH =  5,    # !time from IH to H: infected and will need␣
↪hospitalization to hospitalization
                    t_HR =  8,    # !time from HR to R: hospitalized and will␣
↪recover to recovered
                    t_HC =   6,    # !time from HC to C: hospitalization to␣
↪critical care
                    t_CD =   10,# time from CD to D: critical care and will␣
↪die to death
                    t_CL =   7,    # time from CR to L: critical care and will␣
↪recover to post-critical care hospitalization
                    t_LR =  3,    # time from L to R: post-critical care␣
↪hospitalization to recovered

                    p_IA = 1/3, # Proportions of infections that are␣
↪asymptomatic
                    p_IH = 0.066, # Proportion of symptomatic infections that␣
↪require hospitalizations, conditional on not being asymptomatic
                    p_HC = 0.3, # Proportion of hospitalizations that require␣
↪critical care
                    p_CD = 0.5, # Proportion of critical cases that are fatal
                    change_in_p_IH = 1, # How much is the probability of␣
↪hopsitalization increased in OLD
                    change_in_p_IA = 1, # How much is the probability of␣
↪asymptomatic increased in OLD
                    adjust_beta_hosp = adjust_beta_hosp) # change the beta␣
↪for within hospital interactions relative to beta between I and S


    single_population    = np.zeros(len(compartment_names))
    single_population[1] = n_exposed
```

```
    single_population[2] = pred_infected*args.p_IA
    single_population[3] = pred_infected*(1-args.p_IA)*(1-args.p_IH)
    single_population[4] = pred_infected*(1-args.p_IA)*(args.p_IH)
    single_population[0] = N - sum(single_population)
    initial_state        = [(1 - prop_essential) * single_population,
                            prop_essential * single_population]
    # Convert initial state into 1-dimensional array
    initial_state = [params for group in initial_state for params in group]

    sol = solve_ivp(lambda t,y: SEIR_HCD_model(t,y,args), t_span,␣
↪initial_state, t_eval=t_eval,method = 'Radau')
    pred_all = np.maximum(sol.y, 0)
    pred_all = get_compartment_counts(sol, num_groups)

    min_loss = np.inf
    best_start = 0

    for i in range(int(lockdown_t)):
        begin_time = i
        n_train = len(n_deaths)
        # Get Predicted Deaths and Hospitalization from days that we want to␣
↪fit to
        pred_cases, pred_hosp, pred_deaths = pred_all[0], pred_all[1],␣
↪pred_all[2]
        pred_deaths_eval = pred_deaths[begin_time: n_train + begin_time]

        # Use only if we want to also fit to the hospitilization curve (only␣
↪useful for NYC)
        if fit_hospital == True:
            n_hospitilized = data['cumulative_hospitalized']
            pred_hosp_eval = pred_hosp[begin_time: n_train + begin_time]
            n_hospitilized_diffs = np.ediff1d(n_hospitilized,␣
↪to_begin=n_hospitilized[0])[1:]
            pred_hosp_eval_diffs = np.ediff1d(pred_hosp_eval,␣
↪to_begin=pred_hosp_eval[0])[1:]
            hosp_rmse = np.sqrt(mean_squared_error(n_hospitilized_diffs,␣
↪pred_hosp_eval_diffs))

        n_deaths_diffs = np.ediff1d(n_deaths, to_begin=n_deaths[0])[1:]
        pred_deaths_eval_diffs = np.ediff1d(pred_deaths_eval,␣
↪to_begin=pred_deaths_eval[0])[1:]

        deaths_rmse = np.sqrt(mean_squared_error(n_deaths_diffs,␣
↪pred_deaths_eval_diffs))

        if fit_hospital == True:
```

```
            loss = hosp_rmse

        else:
            loss = deaths_rmse

        if loss < min_loss:
            min_loss = loss
            best_start = i
            best_pred_cases, best_pred_hosp, best_pred_deaths = pred_cases,␣
↪pred_hosp, pred_deaths

    if error == True:
        return min_loss

    else:
        return min_loss, best_start, best_pred_cases[best_start:],␣
↪best_pred_hosp[best_start:], best_pred_deaths[best_start:]
```

We use a hierarchical optimization approach. First, we use Latin Hypercube Sampling to generate random starting points for the parameters to infer. Next, we use Nelder-Mead optimization for the intial guesses to obtain a good starting point for a final standard descent optimization with the L-BFGS algorithm. This process is repeated 5 times and the parameters with the minimal loss are taken as the final value.

[68]:
```
def fit_SEIR_model(data, model, N=8.1e6, fit_hospital=False):

    # decide how many days to forcast
    n_forecast = 275

    min_loss = np.inf
    best_parameters = None

    # Model, Initial values, and bounds on parameters
    print(model)

    # R_0, theta, I0
    x0 = [1, 0, 0, 0]

    # Loop over potential times for lockdown
    for i in [50]:#np.arange(25,75):
        for _ in range(5):
            print('\t',i)

            # bounds on R_0, theta
            bounds = ((3,3),(0, 1),(30,80),(0,0))
            mm = len(x0)
```

```
            simplex = lhs(mm,(mm+1),'center')
            for ii,(minv,maxv) in enumerate(bounds):
                simplex[:,ii] = simplex[:,ii]*(maxv-minv)+minv

            print(simplex)

            bounds = ((1, 10),(0, 1),(30,80),(0,0))
            # Find the optimal set of parameters given model and parameter␣
↪values and bounds
            res = minimize(fun = SEIR_model_loss,
                           x0 = x0,
                           method = 'Nelder-Mead',
                           options = {'initial_simplex':simplex},
                           args=(data, n_forecast, model, i, 0, N,␣
↪fit_hospital))

            res = minimize(fun = SEIR_model_loss,
               x0 = res.x,
               bounds = bounds,
               args=(data, n_forecast, model, i, 0, N, fit_hospital))


            R_0, theta, lockdown_t  = res.x[0:3]

            # Simulate data with given parameters and returns outcomes: error,␣
↪predicted cases, predicted deaths, and predicted deaths
            # j in the best start day (how many days since the first infection␣
↪until the first case in NYC data)
            err, j,pred_cases, pred_hosp, pred_deaths = SEIR_model_loss(res.x,␣
↪data=data, n_forecast=n_forecast, model=model,
                                                                                  ␣
↪lockdown_t=i,start_t=0,error=False, N=N, fit_hospital=fit_hospital)

            # Print the best parameters for a given lockdown day
            print('\t\t', j, R_0, theta, err)

            # If this new set of parameters is the best one, save it
            if err < min_loss:
                start_t = j
                lockdown_t = i
                best_parameters = res.x
                min_loss = err

    R_0, theta = best_parameters[0:2]
    estimate_text = '\tR_0 = {}\n\t'.format(round(R_0,2)) + \
```

```
                'theta = {}\n\t'.format(round(theta,3)) + \
                'lockdown_time = {}\n\t'.format(round(lockdown_t,1)) + \
                'start_time = {}'.format(round(start_t,1))
    print(estimate_text)

    err, start_t, pred_cases, pred_hosp, pred_deaths =␣
↪SEIR_model_loss(best_parameters,
                                                                        ␣
↪data=data,
                                                                        ␣
↪n_forecast=n_forecast,
                                                                        ␣
↪model=model,
                                                                        ␣
↪lockdown_t=lockdown_t,
                                                                        ␣
↪start_t=start_t,
                                                                        ␣
↪error=False, N=N,fit_hospital=fit_hospital)
    # Concatenate actual and predicted values into one dataframe
    # Get dates relating actual data to predicted data
    n = len(pred_cases)

    dates_all = [str(datetime.strptime(data['DATE_OF_INTEREST'].astype(str)[0],␣
↪'%Y-%m-%d') + timedelta(days = ii))[0:10] for ii in range(n)]
    predictions = pd.DataFrame({'ConfirmedCases_pred': pred_cases,␣
↪'Fatalities_pred': pred_deaths,'Hospitilized_pred': pred_hosp}, index =␣
↪dates_all)

    actuals = data[['DATE_OF_INTEREST','cumulative_cases','cumulative_deaths']]
    if fit_hospital == True:
        actuals =␣
↪data[['DATE_OF_INTEREST','cumulative_cases','cumulative_deaths','cumulative_hospitalized']]
    actuals.DATE_OF_INTEREST = actuals.DATE_OF_INTEREST.astype(str)
    actuals.set_index('DATE_OF_INTEREST', inplace=True)
    # Merge the two dataframes
    plot_df = pd.merge(predictions, actuals, how='left', left_index=True,␣
↪right_index=True)

    return((R_0,theta,lockdown_t,start_t), plot_df)

# Here we are only fitting the 'No Structure' model. However, other valid␣
↪models can be inserted into the list below
models = ['No Structure']
fitting_results_nyc = {}
fitting_results_seattle = {}
```

```python
fitting_results_chicago = {}

for model in models:
    param_estimate, plot_df = fit_SEIR_model(nyc_cases, model)
    fitting_results_nyc[model] = (param_estimate, plot_df)

for model in models:
    param_estimate, plot_df = fit_SEIR_model(seattle_cases, model, N=2.2e6)
    fitting_results_seattle[model] = (param_estimate, plot_df)

for model in models:
    param_estimate, plot_df = fit_SEIR_model(chicago_cases, model, N=5e6)
    fitting_results_chicago[model] = (param_estimate, plot_df)
```

```
No Structure
         50
[[ 3.   0.9 55.   0. ]
 [ 3.   0.7 75.   0. ]
 [ 3.   0.5 65.   0. ]
 [ 3.   0.3 45.   0. ]
 [ 3.   0.1 35.   0. ]]
                 53 3.0062629140594965 3.107220510567528e-13 48.167979446430465
         50
[[ 3.   0.3 35.   0. ]
 [ 3.   0.1 55.   0. ]
 [ 3.   0.5 65.   0. ]
 [ 3.   0.7 45.   0. ]
 [ 3.   0.9 75.   0. ]]
                 53 3.00730236777992 1.0943125753210585e-09 48.17231124913149
         50
[[ 3.   0.7 55.   0. ]
 [ 3.   0.3 75.   0. ]
 [ 3.   0.5 65.   0. ]
 [ 3.   0.9 35.   0. ]
 [ 3.   0.1 45.   0. ]]
                 53 3.0072349898869213 1.2163278670447288e-13 48.181417323957646
         50
[[ 3.   0.5 75.   0. ]
 [ 3.   0.7 55.   0. ]
 [ 3.   0.3 65.   0. ]
 [ 3.   0.9 35.   0. ]
 [ 3.   0.1 45.   0. ]]
                 53 3.004305300101542 1.092274492476614e-09 48.156405934274744
         50
[[ 3.   0.9 65.   0. ]
 [ 3.   0.1 45.   0. ]
 [ 3.   0.3 35.   0. ]
```

```
 [ 3.   0.7 55.   0. ]
 [ 3.   0.5 75.   0. ]]
                  53 3.00727313554032 1.7838658494355415e-13 48.176257064879486
        R_0 = 3.0
        theta = 0.0
        lockdown_time = 64.2
        start_time = 53
No Structure
         50
[[ 3.   0.3 45.   0. ]
 [ 3.   0.9 65.   0. ]
 [ 3.   0.5 35.   0. ]
 [ 3.   0.7 55.   0. ]
 [ 3.   0.1 75.   0. ]]
                  30 2.9999379354922393 0.29745172867778086 3.294445202937709
         50
[[ 3.   0.3 45.   0. ]
 [ 3.   0.7 65.   0. ]
 [ 3.   0.9 75.   0. ]
 [ 3.   0.1 55.   0. ]
 [ 3.   0.5 35.   0. ]]
                  30 3.000696123516394 0.2973677499019996 3.294456241795243
         50
[[ 3.   0.5 45.   0. ]
 [ 3.   0.3 65.   0. ]
 [ 3.   0.1 75.   0. ]
 [ 3.   0.9 55.   0. ]
 [ 3.   0.7 35.   0. ]]
                  30 2.99999974944433 0.29743572158350956 3.2944458476308847
         50
[[ 3.   0.9 65.   0. ]
 [ 3.   0.7 45.   0. ]
 [ 3.   0.5 35.   0. ]
 [ 3.   0.3 55.   0. ]
 [ 3.   0.1 75.   0. ]]
                  30 3.0013153868629807 0.29719993284217083 3.294465948829463
         50
[[ 3.   0.9 65.   0. ]
 [ 3.   0.3 35.   0. ]
 [ 3.   0.1 75.   0. ]
 [ 3.   0.7 45.   0. ]
 [ 3.   0.5 55.   0. ]]
                  30 2.9996821949949477 0.29750489338234887 3.2944428355804884
        R_0 = 3.0
        theta = 0.298
        lockdown_time = 50
        start_time = 30
No Structure
```

```
      50
[[ 3.   0.5 35.   0. ]
 [ 3.   0.3 65.   0. ]
 [ 3.   0.9 55.   0. ]
 [ 3.   0.7 45.   0. ]
 [ 3.   0.1 75.   0. ]]
                41 3.0007175762868505 0.358223129255972 18.769938615820518
      50
[[ 3.   0.3 75.   0. ]
 [ 3.   0.5 45.   0. ]
 [ 3.   0.7 65.   0. ]
 [ 3.   0.1 35.   0. ]
 [ 3.   0.9 55.   0. ]]
                41 3.000907313246899 0.3581489777456311 18.769937678392353
      50
[[ 3.   0.7 45.   0. ]
 [ 3.   0.9 65.   0. ]
 [ 3.   0.1 35.   0. ]
 [ 3.   0.3 55.   0. ]
 [ 3.   0.5 75.   0. ]]
                41 3.0003005706674664 0.3583500197656431 18.769949428137757
      50
[[ 3.   0.5 65.   0. ]
 [ 3.   0.9 55.   0. ]
 [ 3.   0.3 75.   0. ]
 [ 3.   0.1 45.   0. ]
 [ 3.   0.7 35.   0. ]]
                41 3.000877679876436 0.35816910463517815 18.769937703403603
      50
[[ 3.   0.3 55.   0. ]
 [ 3.   0.9 65.   0. ]
 [ 3.   0.5 35.   0. ]
 [ 3.   0.7 75.   0. ]
 [ 3.   0.1 45.   0. ]]
                41 3.000476080551965 0.35830373666823334 18.769943408291816
        R_0 = 3.0
        theta = 0.358
        lockdown_time = 44.2
        start_time = 41
```

[69]:
```python
# The function below can be used to display the predicted and actual death
 ↪curves for a give city/location
update_font_size(10,rcParams = mpl.rcParams)
import matplotlib.dates as mdates
from datetime import datetime, date

def plot_model_fits(fitting_results: dict, location, fit_hospital = False):
```

```python
    models = list(fitting_results.keys())

    param_estimate = fitting_results['No Structure'][0]
    plot_df = fitting_results['No Structure'][1]

    plot_df['DATE_OF_INTEREST'] = plot_df.index
    plot_df['DATE_OF_INTEREST'] = pd.to_datetime(plot_df['DATE_OF_INTEREST'])

    fig, ax = plt.subplots(figsize=(12, 8))
    plt.gca().xaxis.set_major_formatter(mdates.DateFormatter('%Y-%m-%d'))
    plt.gca().xaxis.set_major_locator(mdates.DayLocator(interval=10))
    if fit_hospital == False:
        plt.plot(plot_df.DATE_OF_INTEREST,plot_df.Fatalities_pred.values,␣
 ↪color='blue',linestyle='--', linewidth=1.5, label='Fatalities (pred)')
        plt.plot(plot_df.DATE_OF_INTEREST,plot_df.cumulative_deaths.values,␣
 ↪color='red', label='Fatalities (actual)',ls='-',linewidth=3)

    else:
        plt.plot(plot_df.DATE_OF_INTEREST,plot_df.Hospitilized_pred.values,␣
 ↪color='blue',linestyle='--', linewidth=1.5, label='Hospitalizations (pred)')
        plt.plot(plot_df.DATE_OF_INTEREST,plot_df.cumulative_hospitalized.
 ↪values, color='orange', label='Hospitalizations (actual)',ls='-',linewidth=3)
    plt.gcf().autofmt_xdate()

    plt.xlabel('Date', fontsize=12)
    plt.ylabel('Number of Individuals', fontsize=12)
    plt.legend(loc='lower right')
    plt.xlim([date(2020, 3, 1), date(2020, 6, 1)])
    plt.yscale('log')

    #print(param_estimate)
    R_0,theta,lockdown_t,start_t = param_estimate
    estimate_text = r'$R_0 = {}, \theta = {}$'.
 ↪format(round(R_0,2),round(theta,3)) + '\n' + r'$start_t = {}, lockdown_t =␣
 ↪{}$'.format(round(start_t,3),round(lockdown_t,3))

    plt.text(0.5,0.
 ↪9,estimate_text,horizontalalignment='center',verticalalignment='center',transform␣
 ↪= ax.transAxes)
    plt.show()

# #Plot the fits for Seattle, Chicago and NYC sequentially
plot_model_fits(fitting_results_seattle, "Seattle")
plot_model_fits(fitting_results_chicago, "Chicago")
plot_model_fits(fitting_results_nyc, "NYC")
```

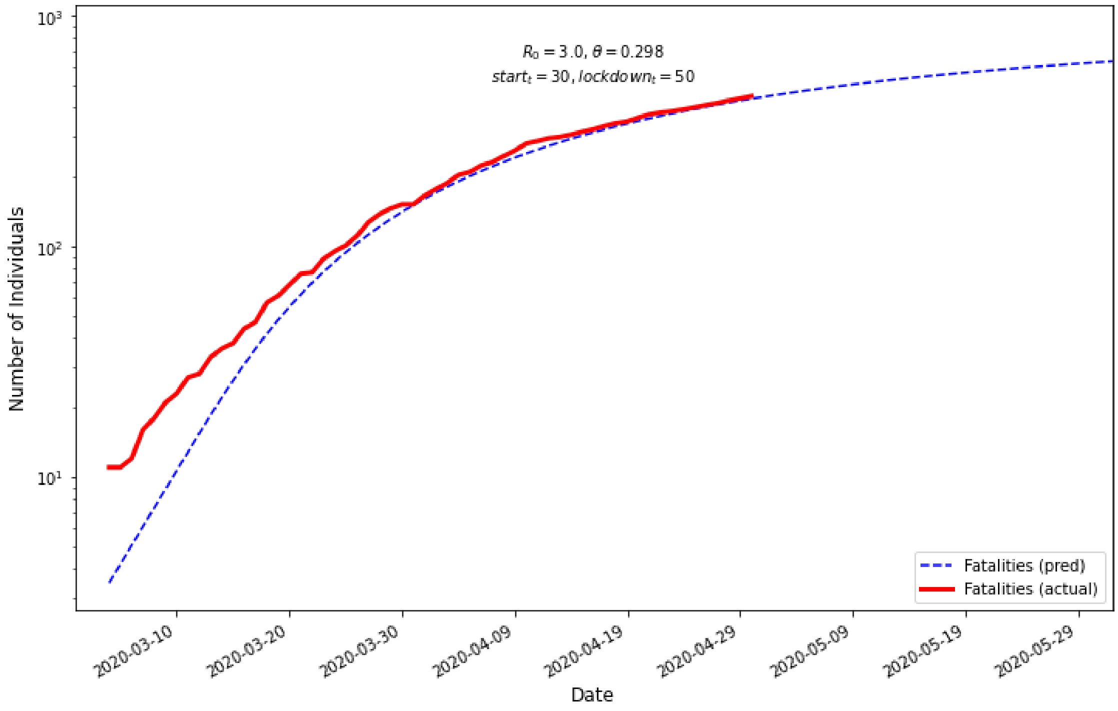
$R_0 = 3.0, \theta = 0.298$
$start_t = 30, lockdown_t = 50$
Number of Individuals
Date
Fatalities (pred)
Fatalities (actual)

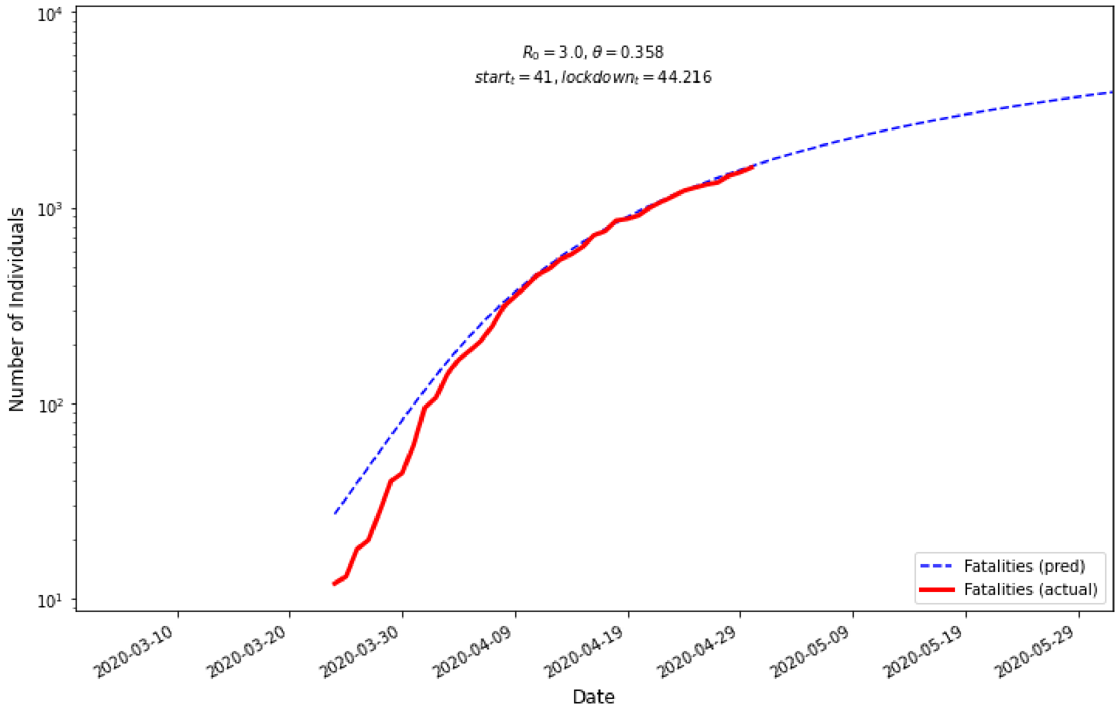
$R_0 = 3.0, \theta = 0.358$
$start_t = 41, lockdown_t = 44.216$
Number of Individuals
Date
Fatalities (pred)
Fatalities (actual)

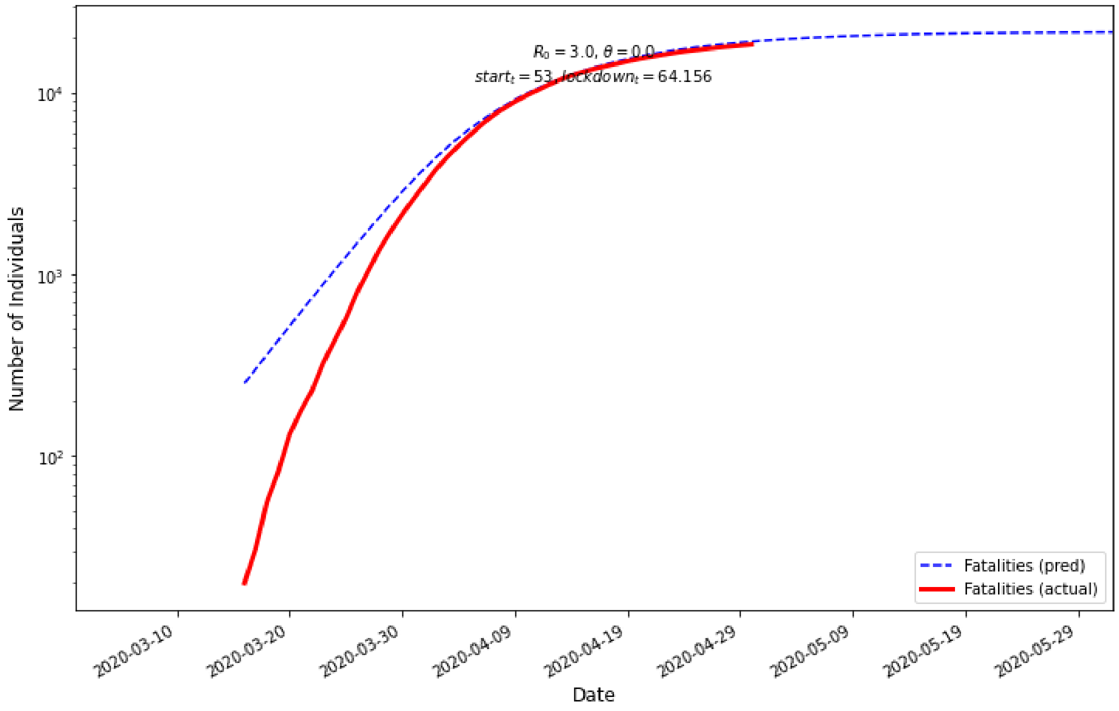

Figure S49. Predicted COVID-19 deaths compared to actual deaths for Seattle (top), Chicago (middle), and New York City (bottom).

In the plots above, the observed number of deaths is shown in red and the predictions from a model with no structure in blue. As can be seen, a good fit and plausible parameter estimates are obtained for data from Chicago and Seattle. A reasonable fit is also seen for New York City, but in this case, parameter estimates are not so sensible (notably θ=0), possibly because the data are less reliable (see above). Given these possible issues, and the fact that New York City also provides hospitalization counts, we instead explored fitting our model to the cumulative hospitalizations for this case.

[70]:
```
models = ['No Structure']
fitting_results_hosp_nyc = {}
#nyc_hosp_cases = nyc_cases[nyc_cases['DATE_OF_INTEREST']> '3/11/2020']
#nyc_hosp_cases.reset_index(drop=True, inplace=True)

for model in models:
    param_estimate, plot_df = fit_SEIR_model(nyc_cases, model,␣
 ↪fit_hospital=True)
    fitting_results_hosp_nyc[model] = (param_estimate, plot_df)
```

```
No Structure
         50
[[ 3.   0.7 55.   0. ]
 [ 3.   0.5 65.   0. ]
```

```
 [ 3.   0.1 45.   0. ]
 [ 3.   0.9 35.   0. ]
 [ 3.   0.3 75.   0. ]]
                45 3.000075939839857 0.5070777862398328 145.29567066566204
        50
[[ 3.   0.3 45.   0. ]
 [ 3.   0.9 35.   0. ]
 [ 3.   0.7 65.   0. ]
 [ 3.   0.1 55.   0. ]
 [ 3.   0.5 75.   0. ]]
                45 3.0001378079898164 0.5070682617170528 145.29498184417673
        50
[[ 3.   0.3 45.   0. ]
 [ 3.   0.1 65.   0. ]
 [ 3.   0.7 55.   0. ]
 [ 3.   0.9 75.   0. ]
 [ 3.   0.5 35.   0. ]]
                45 2.9991141434846047 0.5067176603904661 145.25836165813791
        50
[[ 3.   0.9 35.   0. ]
 [ 3.   0.7 55.   0. ]
 [ 3.   0.5 65.   0. ]
 [ 3.   0.1 45.   0. ]
 [ 3.   0.3 75.   0. ]]
                45 3.000075939839857 0.5070777862398328 145.29567066566204
        50
[[ 3.   0.3 45.   0. ]
 [ 3.   0.5 75.   0. ]
 [ 3.   0.9 65.   0. ]
 [ 3.   0.7 35.   0. ]
 [ 3.   0.1 55.   0. ]]
                45 3.000133764111574 0.5070491554404155 145.2950800218302
        R_0 = 3.0
        theta = 0.507
        lockdown_time = 53.3
        start_time = 45
```

[71]:
```
plot_model_fits(fitting_results_hosp_nyc, "NYC", fit_hospital=True)
```

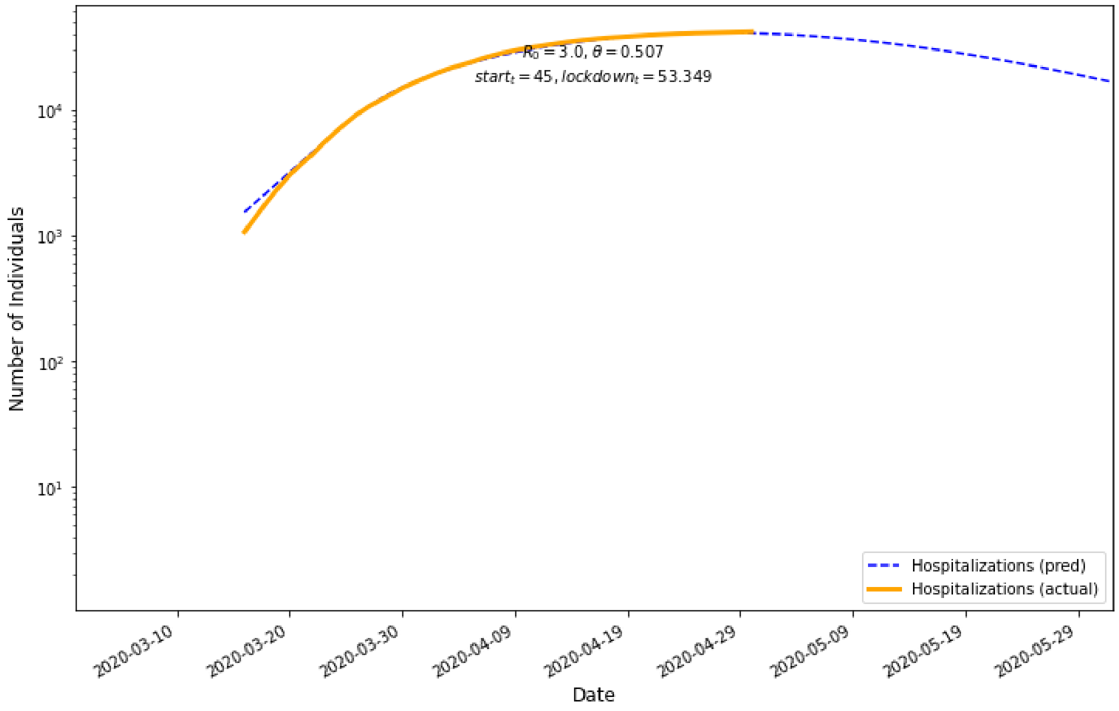

Figure S50. Predicted COVID-19 hospitalizations compared to actual hopsitalizations for New York City, Chicago, and Seattle.

Here, the parameter estimates are perhaps more sensible.

0.1.6 S6 - Parameterizing β_{hosp}

Under the healthcare model, we allow infected hospital patients to infect susceptible healthcare workers. We parameterize the interaction term of this additional route of infections as $\beta_{hosp} = cf\beta_T$ where c is a parameter. We choose the value of c based on κ, the resulting relative risk of a nEW infecting a healthcare worker vs infecting another nEW. The code below calculated the relationship between κ, the relative risk to healthcare workers, and the coefficient c of β_{hosp}.

[72]:
```
fig = plt.figure(figsize=(10,7))

labels = ['Proportionate Mixing','Elevated in-group mixing','No Between Group␣
 ↪Mixing']
colors = ['black',model_colors['Healthcare'],model_colors['USPS']]
update_font_size(15)

prop_essential = 0.05
x_values = np.linspace(0,20,10000)

p = args_default
```

```
avg_time_infectious = (p.p_IA * p.t_AR + (1 - p.p_IA) * ((1-p.p_IH) * p.t_IR +␣
↪p.p_IH * p.t_IH))
avg_time_critical = (1 - p.p_CD) * (p.t_CL + p.t_LR) + p.p_CD * (p.t_CD)
avg_time_hospital = (1 - p.p_HC) * p.t_HR + p.p_HC * (p.t_HC +␣
↪avg_time_critical)
avg_time_hospital =  (1 - p.p_IA) * p.p_IH * avg_time_hospital

for rho in [1,0.5,0]:

    G = ((1 - rho) + x_values * avg_time_hospital/
↪(avg_time_infectious))*(1-prop_essential)
    H = 1 - (1 - rho) * prop_essential
    plt.plot(G/H,x_values,label=labels.pop(),color=colors.pop())
    plt.ylabel(r'$c$')
    plt.xlabel('$\kappa$: Relative risk to healthcare workers')
    plt.legend()

    print(rho,x_values[np.where(np.abs(G/H-1.5) == min(np.abs(G/H-1.5)))])
print(max(G/H))
plt.vlines([1.5],ymin = 0, ymax = max(x_values),color=[0.2]*3+[1],ls=':')
plt.show()
```

```
1 [17.25172517]
0.5 [11.35713571]
0 [5.46254625]
2.8304
```

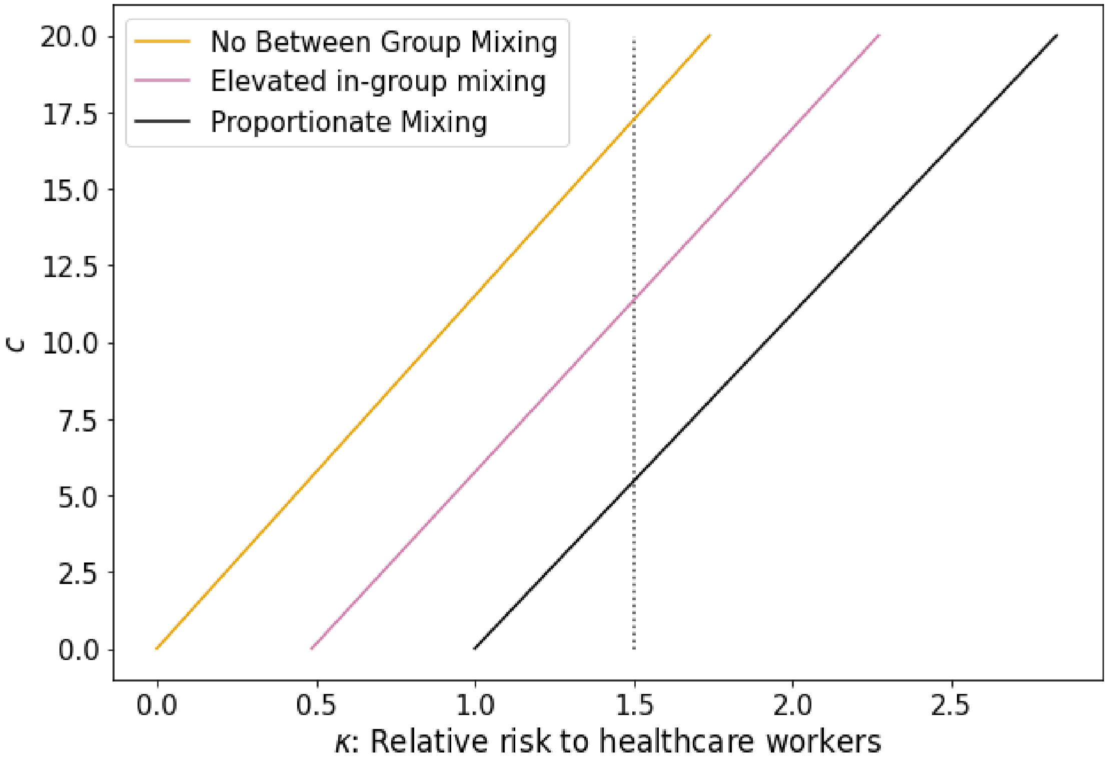

Figure S50. Relationship between κ and c

The above figure shows that the coefficient c increases linearly with κ under different values of ρ. This is because higher rates of infection between patients and healthcare workers cause a greater rate of infection in hospital workers compared to nEWs

Including this additional route of infections also increase R_0 while decreasing the doubling time. However, for the parameters we consider, the figure below demonstrates that this effect is small.

[73]:
```
prop_essential = 0.05


# get new R_0  including within hospital infections
r0 = R_0 + avg_time_hospital * x_values * R_0/avg_time_infectious *␣
 ↪prop_essential

# get kappa from beta_hosp coefficients
rho = 0.5
G = ((1 - rho) + x_values * avg_time_hospital/
 ↪(avg_time_infectious))*(1-prop_essential)
H = 1 - (1 - rho) * prop_essential
GH = G/H

update_font_size(15)
```

```
# plot increased R0
fig = plt.figure(figsize=(10,7))
ax_color = [0.1,0.1,0.8]
plt.plot(G/H,r0,linewidth=2.5,color=ax_color)
plt.ylabel(r'$R_0$',color = ax_color)
plt.xlabel('Relative risk to healthcare workers')
ax = plt.gca()
ax.spines['left'].set_color(ax_color)
ax.tick_params(axis='y', colors=ax_color)

#plot decreased doubling time
ax1 = ax.twinx()
plt.sca(ax1)
ax1_color = [0.0, 0.6196078431372549, 0.45098039215686275, 1]
d = (args_default.t_inc + avg_time_infectious + avg_time_hospital *␣
 ↪prop_essential * x_values) * np.log(2) / (r0 - 1)
plt.plot(G/H,d,linewidth = 2.5,color=ax1_color)
ax1.spines['right'].set_color(ax1_color)
ax1.tick_params(axis='y', colors=ax1_color)
ax1.set_ylabel('Doubling Time',color=ax1_color)
```

[73]: Text(0, 0.5, 'Doubling Time')

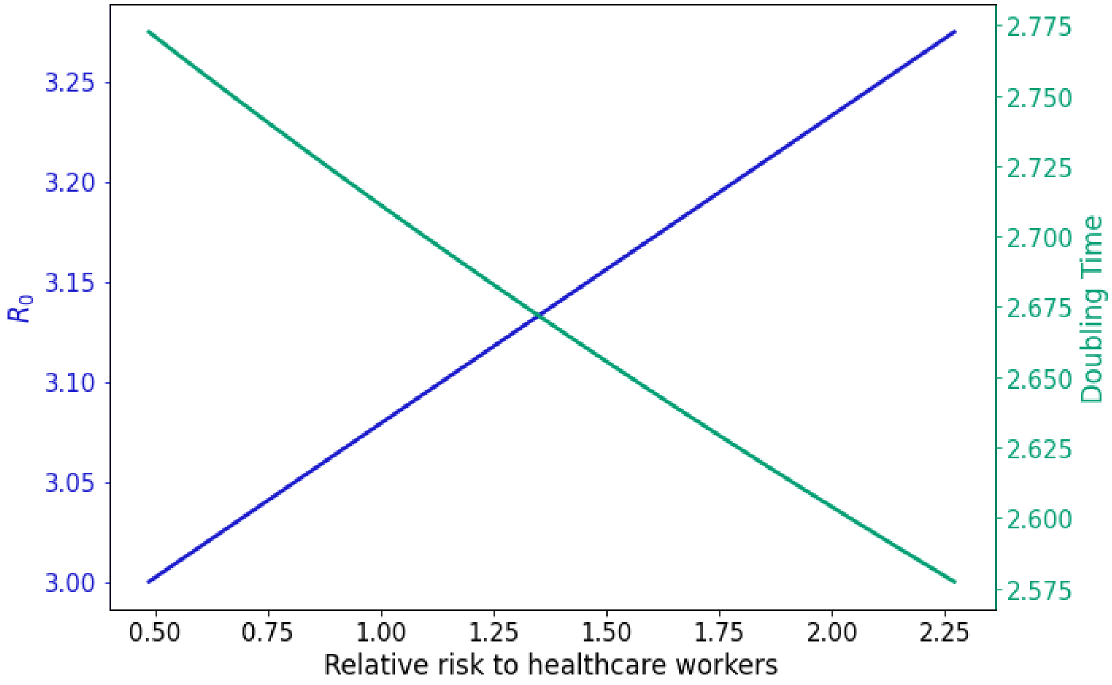

Figure S51. Effect of relative risk to healthcare workers on R_0 and doubling time.

As the risk to healthcare workers increases from 0 to 2.25, the changes to R_0 and the doubling time are only about 10% of their original values. The code below calculates the effects of changing κ on different model outcomes.

[74]:
```
def vary_kappa_hosp(theta_values, healthcare_model,t_measure = 100,
                    models = ['No Structure'],
                    max_days = 500,
                    R_0=3.2,
                    I0=20,
                    f = 0.05,
                    rho = 0,
                    model_dependent_f = {'No Structure': 0.05},
                    model_dependent_rho = {'No Structure': 0},
                    t_lockdown = args_default.social_distancing_time,
                    adjust_beta_hosp = [adjust_beta_hosp]):

    results = lambda s: (sum(s[1,1:,100]),
                         sum(s[1,1:,-1]),
                         sum(s[0,1:,-1]))

    solution_results_all = {}
    comparison_results_all = {}

    for theta in theta_values:
        solution_results = {}
        for index,gamma in enumerate(adjust_beta_hosp):

            solution,p = run_model(theta = theta,
                                   R_0 = R_0,
                                   t_lockdown = t_lockdown,
                                   model = healthcare_model,
                                   n_infected = I0,
                                   n_exposed  = 0,
                                   prop_essential = f,
                                   max_days = max_days,
                                   rho = rho,
                                   adjust_beta_hosp = gamma*f,
                                   doubling_time = 3)

            solution_matrix = np.reshape(solution.y,[p.num_groups,len(p.
↪compartment_names),p.max_days])


            avg_solution = (solution_matrix[0,:,:]+solution_matrix[1,:,:])/8e6

            solution_matrix[0,:,:] = solution_matrix[0,:,:]/(8e6*(1-f))
            solution_matrix[1,:,:] = solution_matrix[1,:,:]/(8e6*f)
```

```
            R_effective, Betas, gg = get_R_effective(solution_matrix,p)
#             print(gamma,get_infectious_beta(p),sum(Betas[:,0,0]),Betas[:,:,0])

            solution_results[gamma] = results(solution_matrix)

        comparison_results = {}
        for index,model in enumerate(models):

            f1 = model_dependent_f[model]
            rho1 = model_dependent_rho[model]

            solution,p = run_model(theta = theta,
                                   R_0 = R_0,
                                   t_lockdown = t_lockdown,
                                   model = model,
                                   n_infected = I0,
                                   n_exposed  = 0,
                                   prop_essential = f1,
                                   max_days = max_days,
                                   rho = rho1,
                                   adjust_beta_hosp = 0,
                                   doubling_time = 3)

            solution_matrix = np.reshape(solution.y,[p.num_groups,len(p.
↪compartment_names),p.max_days])

            avg_solution = (solution_matrix[0,:,:]+solution_matrix[1,:,:])/8e6
            solution_matrix[0,:,:] = solution_matrix[0,:,:]/(8e6*(1-f))
            solution_matrix[1,:,:] = solution_matrix[1,:,:]/(8e6*f)

            comparison_results[model] = results(solution_matrix)

        comparison_results_all[theta] = comparison_results
        solution_results_all[theta]   = solution_results

    return solution_results_all,comparison_results_all

# Assign row and column labels for the plots of the effective R over time
def assign_row_label_varyKappa(ax,text,pad = 5, xy = (0,0.5)):

    ax.annotate(text+'\n', xy=xy, xytext=(-ax.yaxis.labelpad - pad, 0),
                xycoords=ax.yaxis.label, textcoords='offset points',
                size='large', ha='center', va='center',rotation=90)

# Label is the value of R_effective
def assign_column_label_varyKappa(ax,index, pad = 5, xy = (0.5,1)):
    if index == 0:
```

```
        label = 'Prevalence in EWs after 100 days'
    elif index == 1:
        label = 'Prevalence in EWs after max days'
    elif index == 2:
        label = 'Prevalence in nEWs after max days'
    pad = 5 # in points
    ax.annotate(label+'\n', xy=xy, xytext=(0, pad),
                xycoords='axes fraction', textcoords='offset points',
                size='large', ha='center', va='baseline')

def plot_vary_kappa(solution_results_all,comparison_results_all,linewidth = 4,␣
↪font_size = 30,figsize = (40,40)):


    # Plotting LS and color for each model
    ls_styles = {'No Structure':('-',model_colors['No Structure'],1),
                 'Cashier':('-',model_colors['Cashier'],100),
                 'Healthcare':('-',model_colors['Healthcare'],100),
                 'USPS':('-',model_colors['USPS'],100),
                 'Healthcare_Sigmoid':('-',model_colors['Healthcare'],100)}

    G = lambda x_values: ((1 - rho) + x_values * avg_time_hospital/
↪(avg_time_infectious))*(1-prop_essential)
    H = 1 - (1 - rho) * prop_essential
    kappa = lambda x_values: G(x_values)/H

    update_font_size(font_size,rcParams = mpl.rcParams)
    fig, axes_all = plt.subplots(ncols = 3,nrows = len(solution_results_all.
↪keys()),figsize=(50,10*len(solution_results_all.keys())))

    for i,((theta,solution_results),(theta,comparison_results)) in␣
↪enumerate(zip(solution_results_all.items(),comparison_results_all.items())):

        for j,ax in enumerate(axes_all[i]):
            x_values = np.sort(list(solution_results.keys()))
            y_values = np.array([solution_results[key][j] for key in x_values])
            ax.plot(kappa(x_values),y_values,linewidth =␣
↪linewidth,color=model_colors['Healthcare'])


            for model,model_values in comparison_results.items():
                ls,color,zorder = ls_styles[model]
                ax.hlines(model_values[j],xmin = min(kappa(x_values)),xmax =␣
↪max(kappa(x_values)),color= color,label=model,linewidth = linewidth)
                if model == 'No Structure':
                    bar_height = model_values[j]*0.9
```

```
            if i == 0:
                assign_column_label_varyKappa(ax=ax,index = j)
            if j == 0:
                assign_row_label_varyKappa(ax=ax,text=r'$R_0 \theta = $' + '{}'.
 ↪format(theta*R_0))
            if j == 0 and i == 0:
                ax.text(x = 1.05,y = 0.3, s = 'Public␣
 ↪Facing',color=model_colors['Cashier'])
                ax.text(x = 1.05,y = 0.5, s = 'Non-Public␣
 ↪Facing',color=model_colors['USPS'])
                ax.text(x = 1.05,y = 0.65, s =␣
 ↪'Healthcare',color=model_colors['Healthcare'])


            plt.sca(ax)
            plt.xlabel(r'$\kappa$')
            plt.ylabel('Proportion Infected')

            plt.vlines(kappa(adjust_beta_hosp),
                       ymin=bar_height,
                       ymax=max(max(y_values),max(list([jj[j] for jj in␣
 ↪comparison_results.values()]))),
                       color = 'k',
                       ls = ':',
                       linewidth = linewidth)

    fig.tight_layout()
    # Tight_layout doesn't take these labels into account. We'll need
    # to make some room. These numbers are are manually tweaked.
    # You could automatically calculate them, but it's a pain.
    fig.subplots_adjust(left=0.15, top=0.85)
    plt.subplots_adjust(wspace=0.3)
    fig.suptitle(f'Figure S{figure_number()}.' + r'Effect of $\kappa$ on model␣
 ↪dynamics')
    plt.show()
```

[75]:

```
keep_R0_constant = False
global_method='Radau'

desired_Reff = np.array([0.5,0.7,0.9])
s_dict,c_dict = vary_kappa_hosp(theta_values = desired_Reff/R_0 ,
                                adjust_beta_hosp = np.linspace(0,20,20),
                                healthcare_model = 'Healthcare',
                                max_days = 300,
```

```
                                        R_0=R_0,
                                        I0=20,
                                        t_lockdown = args_default.
↪social_distancing_time,

                                        rho = 0.5,
                                        models = ['No Structure','Cashier','USPS'],

                                        model_dependent_f = {'No Structure':       0.05,
                                                             'Cashier':            0.05,
                                                             'USPS':               0.05,
                                                             'Healthcare':         0.05,
                                                             'Healthcare_Sigmoid': 0.
↪05},

                                        model_dependent_rho = {'No Structure': 0,
                                                               'Cashier': 0,
                                                               'Healthcare': 0.5,
                                                               'USPS': 0.5,
                                                               'Healthcare_Sigmoid': ␣
↪0})
```

[76]:

```
plot_vary_kappa(solution_results_all=s_dict,comparison_results_all=c_dict,font_size=40)
```

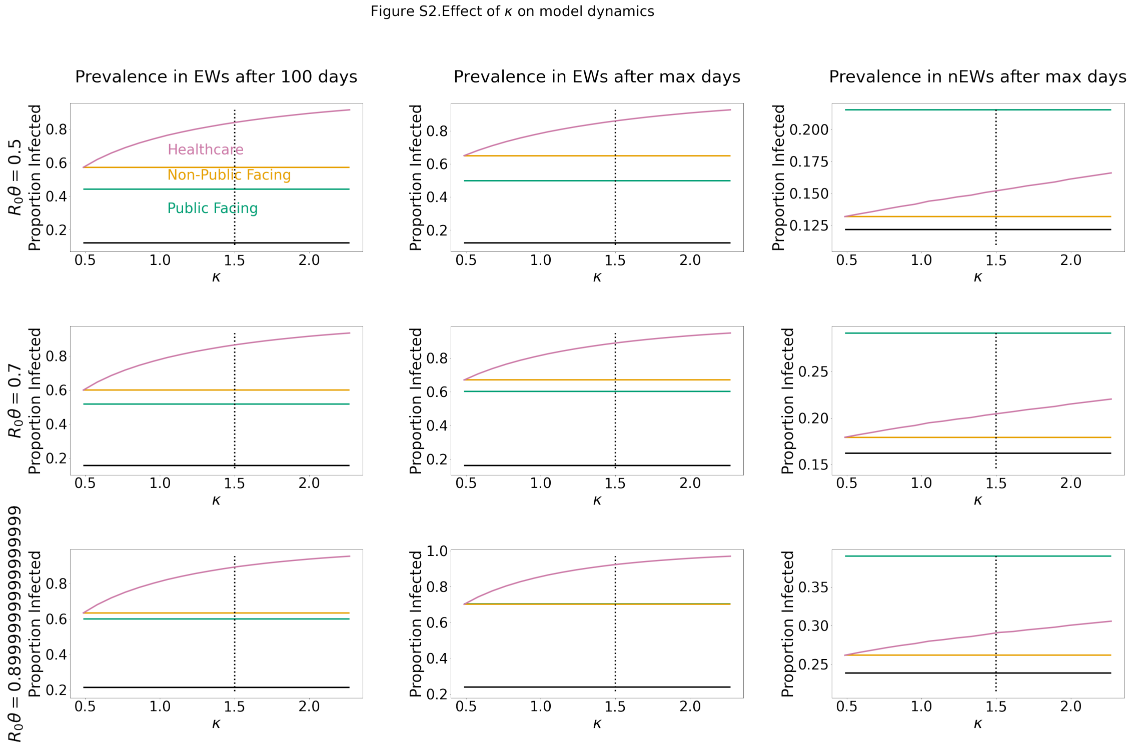

Figure S52. Relationship between κ and infection prevalence.

As κ increases, so does the prevalence in healthcare workers and among nEWs. When $\kappa = 0.5$, the model reduces to the non-public facing worker model.
